# Ancient genomes reveal a deep history of *Treponema pallidum* in the Americas

**DOI:** 10.1038/s41586-024-08515-5

**Published:** 2024-12-18

**Authors:** Rodrigo Barquera, T. Lesley Sitter, Casey L. Kirkpatrick, Darío A. Ramirez, Arthur Kocher, Maria A. Spyrou, Lourdes R. Couoh, Jorge A. Talavera-González, Mario Castro, Tanya von Hunnius, Evelyn K. Guevara, W. Derek Hamilton, Patrick Roberts, Erin Scott, Mariana Fabra, Gabriela V. Da Peña, Aryel Pacheco, Mónica Rodriguez, Eugenio Aspillaga, Anthi Tiliakou, Elizabeth A. Nelson, Karen L. Giffin, Raffaela A. Bianco, Adam B. Rohrlach, María de los Ángeles García Martínez, Fabiola A. Ballesteros Solís, Antti Sajantila, Shelley R. Saunders, Rodrigo Nores, Alexander Herbig, Johannes Krause, Kirsten I. Bos

**Affiliations:** 1 Max Planck Institute for Evolutionary Anthropology, Leipzig, Germany; 2 Western University, London, Ontario Canada; 3 Instituto de Antropología de Córdoba, Consejo Nacional de Investigaciones Científicas y Técnicas, Universidad Nacional de Córdoba, Museo de Antropologías, Córdoba, Argentina; 4 Departamento de Antropología, Facultad de Filosofía y Humanidades, Universidad Nacional de Córdoba, Córdoba, Argentina; 5 Institute for Archaeological Sciences, Eberhard Karls Universität Tübingen, Tübingen, Germany; 6 Dirección de Antropología Física, Instituto Nacional de Antropología e Historia, Mexico City, Mexico; 7 Museo Nacional de Historia Natural, Santiago, Chile; 8 Department of Morphology, Faculty of Medicine, Clínica Alemana–Universidad del Desarrollo, Santiago, Chile; 9 McMaster University, Department of Anthropology, Hamilton, Ontario Canada; 10 Department of Forensic Medicine, University of Helsinki, Helsinki, Finland; 11 Scottish Universities Environmental Research Centre, East Kilbride, UK; 12 Department of Coevolution of Land Use and Urbanisation, Max Planck Institute for Geoanthropology, Jena, Germany; 13 Laboratory Unit, Max Planck Institute of Geoanthropology, Jena, Germany; 14 Consejo Nacional de Investigaciones Científicas y Técnicas, Museo de Ciencias Naturales y Antropológicas Juan C. Moyano, Mendoza, Argentina; 15 Departamento de Arqueología, Facultad de Filosofía y Letras, Universidad Nacional de Cuyo, Mendoza, Argentina; 16 Bioarchaeology Research Group, Durham University, Durham, UK; 17 Independent researcher, Santiago, Chile; 18 Department of Anthropology, University of Chile, Santiago, Chile; 19 School of Computer and Mathematical Sciences, University of Adelaide, Adelaide, South Australia Australia; 20 Adelaide Data Science Centre, University of Adelaide, Adelaide, South Australia Australia; 21 Dirección de Salvamento Arqueológico, Instituto Nacional de Antropología e Historia, Mexico City, Mexico; 22 Forensic Medicine Unit, Finnish Institute of Health and Welfare, Helsinki, Finland; 23 Present Address: Department of Archaeology, Simon Fraser University, Burnaby, British Columbia Canada; 24 Present Address: Department of Anthropology, Dedman College of Humanities and Sciences, Southern Methodist University, Dallas, TX USA

**Keywords:** Archaeology, Bacterial genetics

## Abstract

Human treponemal infections are caused by a family of closely related *Treponema pallidum* that give rise to the diseases yaws, bejel, pinta and, most notably, syphilis^1^. Debates on a common origin for these pathogens and the history of syphilis itself have weighed evidence for the ‘Columbian hypothesis’^2^, which argues for an American origin, against that for the ‘pre-Columbian hypothesis’^3^, which argues for the presence of the disease in Eurasia in the Medieval period and possibly earlier. Although molecular data has provided a genetic basis for distinction of the typed subspecies^4^, deep evolution of the complex has remained unresolved owing to limitations in the conclusions that can be drawn from the sparse palaeogenomic data that are currently available. Here we explore this evolutionary history through analyses of five pre- and peri-contact ancient treponemal genomes from the Americas that represent ancient relatives of the *T. pallidum* subsp. *pallidum* (syphilis), *T. pallidum* subsp. *pertenue* (yaws) and *T. pallidum* subsp. *endemicum* (bejel) lineages. Our data indicate unexplored diversity and an emergence of *T. pallidum* that post-dates human occupation in the Americas. Together, these results support an American origin for all *T. pallidum* characterized at the genomic level, both modern and ancient.

## Abstract (web summary)

Analyses of five treponemal genomes representing ancient relatives of *Treponema pallidum* suggest that this pathogen originated in the Americas.

## Main

In 1494 Charles VIII of France assembled an army from across Western Europe to pursue his claims in Italy. Although he enjoyed brief success, events that followed changed the disease landscape: a disfiguring illness unknown to medical professionals of the time erupted in the army camps and followed the men to their homelands upon their demobilization in 1495. Within five years’ time, the epidemic had spread throughout Europe^5^. A variety of contemporary theories on the origin of the disease circulated, largely reflecting political tensions of the period. In the early sixteenth century, the noted proximity in timing between the onset of the epidemic and Columbus’ maiden voyage across the Atlantic led to the idea that the disease originated on the other side of the ocean, and had been introduced to Europe as a consequence of recent contacts^5^.

The range of symptoms recorded in contemporary sources are thought to describe the first prolific outbreak of syphilis among Europeans, which was observed in both sexually acquired and congenital manifestations^5^. Today we understand this disease to be part of a group of clinically similar infections of bacteria of the genus *Treponema*—namely syphilis (*T. pallidum* subsp. *pallidum* (TPA)), yaws (*T. pallidum* subsp. *pertenue* (TPE)), bejel (*T. pallidum* subsp. *endemicum* (TEN)) and pinta (*Treponema carateum*). Genomic studies reveal that TPA, TPE and TEN share 99% nucleotide sequence identity^4^. The underlying biological factors that contribute to phenotypic variation in disease severity in humans and non-human African primates, their uneven distribution across the globe, and differences in transmission potential have remained poorly understood despite the evaluation of hundreds of genomes^1,6,7^. It is also uncertain whether syphilis, the only *T. pallidum* disease type with a predominantly sexual mode of transmission, is the sole cause of congenital cases^8,9^.

Drawing heavily upon historical documentation, Alfred Crosby^2^ popularized the Columbian hypothesis, which argues that syphilis emerged in the Americas and accompanied Columbus’ crew on one of their return voyages from the Caribbean, possibly as early as January 1493. Additional support for this theory comes from morphological analyses of lesions in archaeological bone associated with chronic treponemal infection that indicate its long history in the Americas (see Supplementary Information, section 1). Others have since revised this hypothesis to accommodate the existence of an ancestral variety in the Americas that rapidly evolved into modern syphilis in response to different selection pressures in terms of host biology, behaviour and climate^10^.

Since its inception, this narrative has raised controversy. Contrary views have looked beyond syphilis and considered the evolutionary history of the disease family as a whole. Early theories were presented before the availability of genetic data—for example, Hudson’s unitarian hypothesis^11^, which proposed that phenotypic differences within the treponemal subspecies are merely environmentally determined expressions of what is functionally an identical organism. Support for the subspecies cladal structure in whole-genome phylogenies of TPA, TPE and TEN refutes this hypothesis in the opinion of some discussants^12^; however, others have cautioned that their degree of similarity could indicate the theory was dismissed prematurely^1^. On the basis of early ideas that treponemal disease was globally distributed before the fifteenth century^13^, models that accommodated hypothetical evolutionary chronologies of the subspecies^3,14^ set the stage for the pre-Columbian hypothesis, which, in simple models, supports a broad and historically deep distribution of infectious treponema in Europe^3^ or Africa^15^, and in more specific models supports the presence of sexually transmitted and congenitally acquired syphilis in Europe^16^ long before Columbus’ first trans-Atlantic expedition. A large body of literature, including two edited volumes, has weighed the skeletal evidence in Europe^17^ and North America^18^, but debates continue about the diagnostic specificity of skeletal pathology associated with chronic or congenital treponemal infection^19,20^ and the accuracy of radiocarbon dates from archaeological bone that span the fifteenth and sixteenth centuries^12,21^. Antibiotic treatments have reduced the frequency of skeletal involvement in treponemal infection today, which further compromises our ability to establish morphologically diagnostic criteria in archaeological tissues.

Ancient DNA is a valuable tool to integrate into the ongoing debates. Although initial expectations were dampened by indications that treponemal DNA would either not preserve^22^ or would be recovered only in rare cases^23^, the number of historic individuals yielding treponemal DNA at the genome level is increasing^9,24–26^. Genetic studies agree in their conclusion that yaws is much younger than previously thought^25,26^, and further indicate that both yaws and syphilis underwent a substantial increase in genetic diversity approximately 500 years ago^25–27^. The small number of ancient genomes yielded from these studies, however, are insufficient to explore the more pressing questions related to the global distribution of these pathogens prior to 1492, in particular those controversies surrounding the origin of syphilis.

Genome-level information from pre-1492 treponemal infections—whether from Europe, Africa or the Americas, where treponemal disease circulated in the early colonial period—are of high value for refining the evolutionary history of *T. pallidum*. Here we offer a substantial contribution to the debate through presentation of five treponemal genomes from pre-contact and peri-contact American contexts. Together these data reveal early relatives for all currently defined *T. pallidum* sublineages, and point toward an extensive unexplored diversity of ancient forms in the Americas.

## Screening and capturing ancient treponemal DNA

**Fig. 1:**
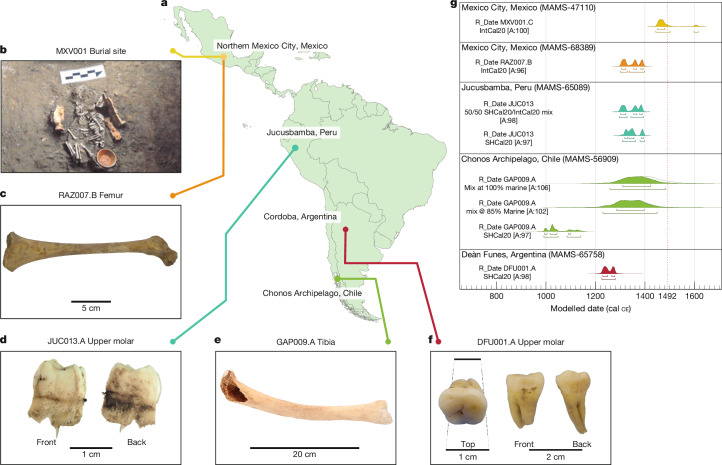
Contextual details of the five individuals who yielded treponemal genomes in this study. **a**, Map showing locations of the archaeological sites where the individuals were found. **b**, Image of skeleton MXV001 in situ in northern Mexico City. **c**, Femur from Mexico City that yielded genome RAZ007. **d**, Immature molar (JUC013.A) with unfused roots that yielded genome JUC013. **e**, Image of the mature tibia (GAP009.A) that yielded genome GAP009. **f**, Upper left second molar (DFU001.A) that yielded genome DFU001. **g**, Calibrated ^14^C radiocarbon data for all individuals. OxCal v4.4.4; r:5 (Bronk Ramsey^51^); atmospheric data from Hogg et al.^52^ and Reimer et al.^53^; marine data from Heaton et al.^54^. For the calibrated date interval, the upper brackets designate 1*σ* ranges (68.3% probability) and the lower brackets designate 2*σ* ranges (95.4% probability).

**Fig. 2:**
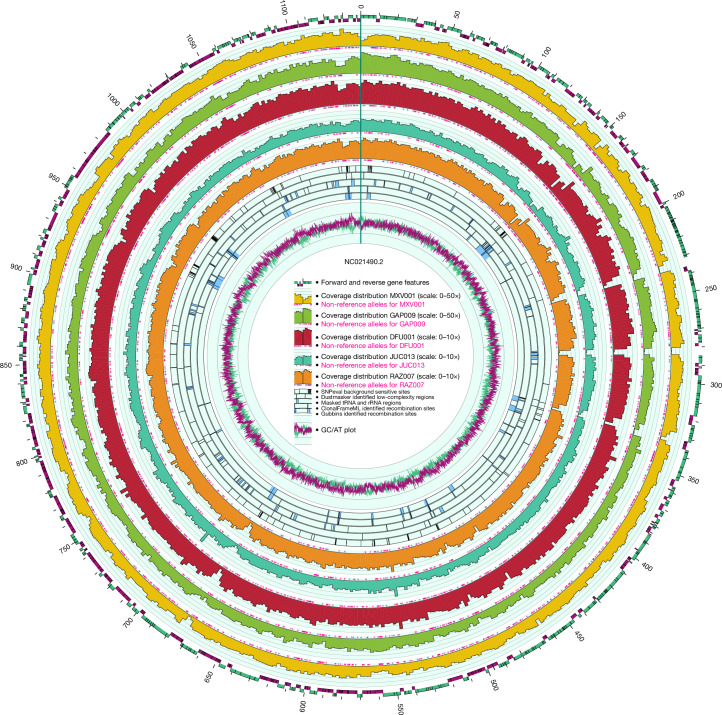
Circos plot showing genome features of the TPA Nichols reference genome and additional tracks containing metadata specific to the five ancient genomes presented here. Gridlines on the average distributions for DFU001, JUC013 and RAZ007 (twofold read support) and GAP009 and MXV001 (fivefold read support). Numbers along the outer circumference indicate coordinates in kb. rRNA, ribosomal RNA.

**Extended Data Fig. 1:**
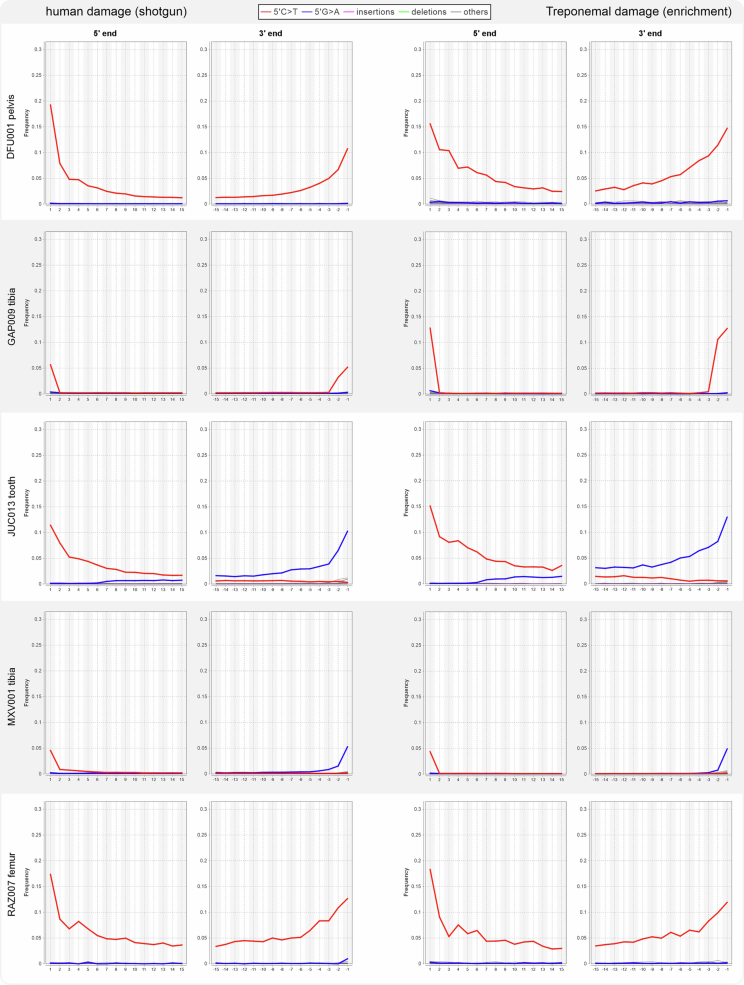
Authentication of ancient treponemal DNA in shotgun and capture libraries via DamageProfiler as implemented in EAGER2 via mapping to the human reference genome (left) and *T. pallidum pallidum* Nichols (right). Damage signals are predictably low in the partial-UDG treated GAP009 and MXV001 libraries.

As a first approach, we sampled from pathological bone displaying lesions consistent with treponemal infection^19^ from multiple sites in Chile (145 tissues from 39 individuals), 41 individuals from Mexico and 1 individual from Argentina (Supplementary Information, section 2 and Supplementary Tables 1 and 2). Teeth and pathological features in bone were preferentially targeted to generate approximately 50 mg of pulverized tissue for molecular extraction. Powders were processed using established techniques with both double-stranded^28^ and single-stranded libraries^29^ constructed for sequencing and analysis. Datasets of bulk DNA content were generated to a depth of around 5 million reads and were screened for the presence of *T. pallidum* DNA via analysis within the MALT/HOPS pipeline^30–32^ (Supplementary Table 3). This process revealed four candidates for genome enrichment: an adult tibial fragment (GAP009) from the Chonos Archipelago of southern Chile, a tibia and humerus (pooled as MXV001) from an infant in Mexico City, a femur and occipital bone (pooled as RAZ007) from a child in Mexico City, and an upper molar, pelvic bone (iliac crest) and rib (pooled as DFU001) from a young adult in Deán Funes, Córdoba Province, in central Argentina (Fig. 1, Supplementary Table 4 and Supplementary Figs. 1–5). In a parallel process, a weak *T. pallidum* signal was identified in one individual from Jucusbamba, Peru (JUC013, an immature molar) via a custom MALT^31^-based TPA computational screening of 36,214 sequencing datasets generated for other research applications at the Max Planck Institute for Evolutionary Anthropology (Supplementary Figs. 6 and 7 and Supplementary Table 4). To maximize chances of DNA recovery, additional libraries were prepared from the extracts and digested products of GAP009, DFU001, RAZ007 and JUC013. Genome-level enrichment for all five candidates was carried out using established protocols^25^. Enriched products were sequenced to depths of 4.8 to 108 million reads, mapped to the TPA Nichols reference genome (RefSeq accession NC_021490.2) in nf-core/eager^32^ (Supplementary Table 5), and single-nucleotide polymorphism (SNP) profiles were generated on the basis of positions with a minimum of fourfold read support. Pooled data from all captured libraries unique to each individual yielded average genome coverages of 25.6-fold, 24.7-fold, 7.2-fold, 4.3-fold and 2.3-fold for MXV001, GAP009, DFU001, JUC013 and RAZ007, respectively (Fig. 2 and Supplementary Table 5). Treponemal captured data for JUC013, DFU001 and RAZ007 (no uracil-DNA glycosylase (UDG) treatment), as well as GAP009 and MXV001 (both partial UDG treatment) showed damage profiles consistent with human DNA, thus supporting their ancient authenticity (Extended Data Fig. 1, Supplementary Table 6 and Supplementary Figs. 8–10). Negative controls were free of *T. pallidum* DNA (Supplementary Table 7).

## Diversity of *T. pallidum* genomes that predate 1492

**Fig. 3:**
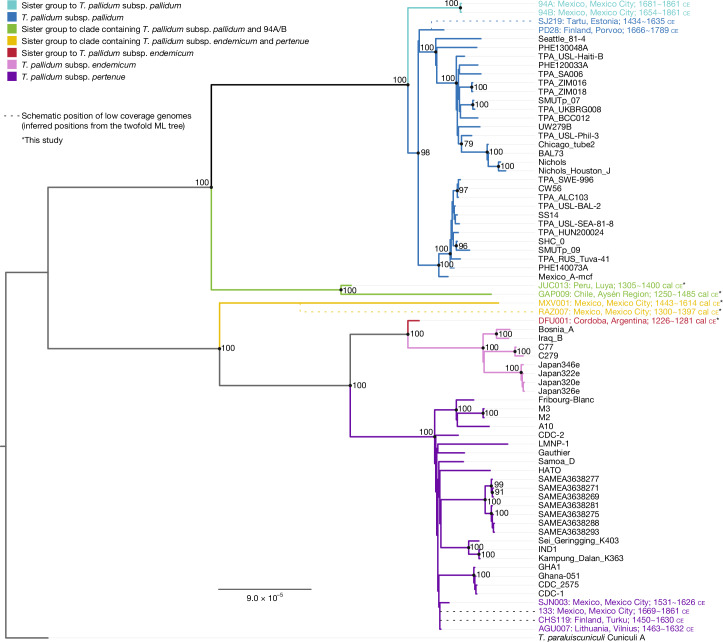
Maximum likelihood-based phylogenetic reconstruction. The reconstruction is based on 2,104 SNPs called at fourfold allele support with recombinant and low-confidence regions removed, and 1,000 bootstrap replicates with 25% permission for missing data. Branches with bootstrap support of at least 70% are represented with a dot and the support value. Names of ancient genomes are coloured according to their corresponding clade. Placements of the lower coverage genomes CHS119, SJ219, 133 and RAZ007 are shown with dotted lines and were made on the basis of their position in trees generated with twofold allele support (Supplementary Fig. 17). Asterisks indicate data generated in this study. ML, maximum likelihood.

**Extended Data Fig. 2:**
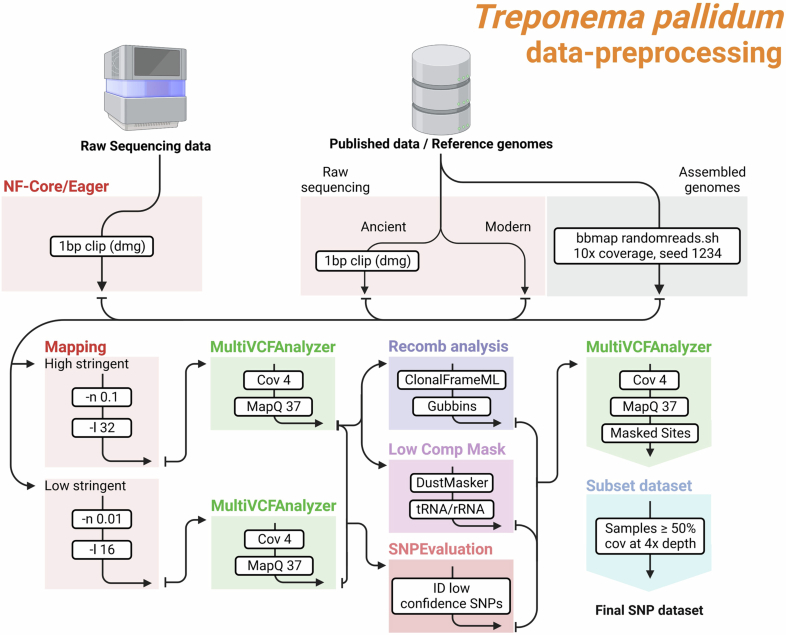
Computational screening pipeline for treponemal data processing of modern and ancient datasets. Image Created in BioRender. Sitter, L. (2024) https://BioRender.com/d39q601.

Placement of these genomes within known treponemal diversity was evaluated with 60 modern genomes (*n* = 28 TPA, *n* = 23 TPE, *n* = 8 TEN and *n* = 1 *Treponema paraluiscuniculi* as an outgroup), along with 9 published genomes of ancient origin (Supplementary Tables 8–10). The threshold for inclusion in phylogenetic analysis was set at a minimum 50% coverage of the genome with fourfold read support (Extended Data Fig. 2). This eliminated the ancient genomes RAZ007 (Mexico City), KM14-7 (Netherlands; Supplementary Information, section 7.4, Supplementary Fig. 11 and Supplementary Tables 11–15), CHS119 (Finland), SJ219 (Estonia) and 133 (Mexico) from further phylogenetic analysis owing to their low coverage (Supplementary Tables 5 and 16–20). Genome ZH1540, previously published as TEN from Brazil around 2000 bp, was also not included (see Majander et al.^33^, as accessed online December 2024). As recombination is commonly observed amongst *T. pallidum* subspecies, genomic regions affected by this phenomenon were removed through filtering of the joined recombinant positions identified in both Gubbins^34^ and ClonalFrameML^35^ following a quality evaluation for heterozygosity in all genomes (Supplementary Figs. 12–14 and Supplementary Tables 18–20). Maximum likelihood trees were generated in RAxML-ng^36^ with 1,000 bootstrap replicates and 25% permission of missing data (Fig. 3 and Supplementary Figs. 15–17). This process revealed that genomes JUC013 and GAP009 branch together as a group paraphyletic to TPA and the 94A/B ancient clade. By contrast, genomes MXV001 and RAZ007 branch together from the lineage common to TPE and TEN. DFU001 forms a sister lineage to the TEN clade. This phylogenetic grouping is maintained when mapping is performed using TPE CDC-2 or TEN Bosnia A as a reference (Supplementary Fig. 16). Where possible, placement of the lower covered genomes was accomplished through phylogenetic reconstruction based on twofold read support (Supplementary Table 10 and Supplementary Fig. 17). Special attention was given to the European genome KM14-7 (1494–1631 ce), whose previously published phylogenetic position showed it to branch from the common lineage of TPE and TEN^26^. Our reanalysis reveals a coverage that is too low for its resolved phylogenetic placement within any *T. pallidum* sublineage (Supplementary Information, section 7.4).

Branch shortening in phylogenetic reconstruction is a common feature of ancient genomes, and is reflective of the reduced time over which derived positions have accumulated^37^. In the case presented here, genomes GAP009 and MXV001 paradoxically do not display this phenomenon (Fig. 3). Through multiple lines of investigation, we conclude that the longer branch lengths in GAP009 and MXV001 do not result from a currently detectable artefact (Supplementary Figs. 15–17 and Supplementary Tables 16 and 17).

**Extended Data Fig. 3:**
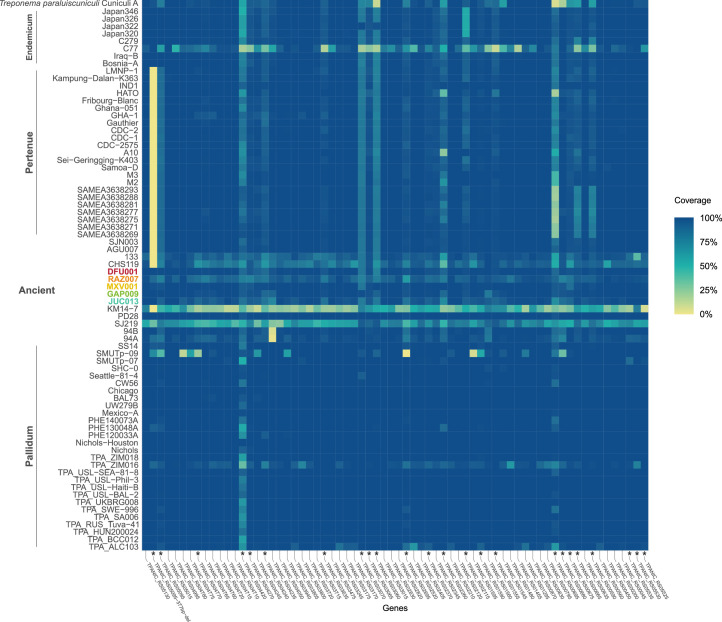
Coverage of putative virulence-associated genes across the Nichols reference genome (NC_021490.2). TEN = *T. pallidum endemicum*, TPE = *T. pallidum pertenue*, and TPA = *T. pallidum pallidum*. Genes indicated with an asterisk are reported to be potentially implicated in phenotypic differences between TPE and TPA (supplementary section 11). For additional information on gene coordinates and function, see Table S24.

For all positions that are either uniquely derived in the ancient genomes, or where JUC013 and GAP009 carry the ancestral allele compared with modern syphilis, at least one-third comprise non-synonymous nucleotide changes with potential functional significance (Supplementary Tables 21–24). Given the antiquity of these genomes, representation of known *T. pallidum* virulence determinates (Supplementary Tables 25–26) was assessed on the basis of regional coverages. This revealed the presence of all known virulence genes in the five ancient genomes featured here, with predictable reduced coverage across them for genomes JUC013 and RAZ007 (Extended Data Fig. 3).

**Fig. 4:**
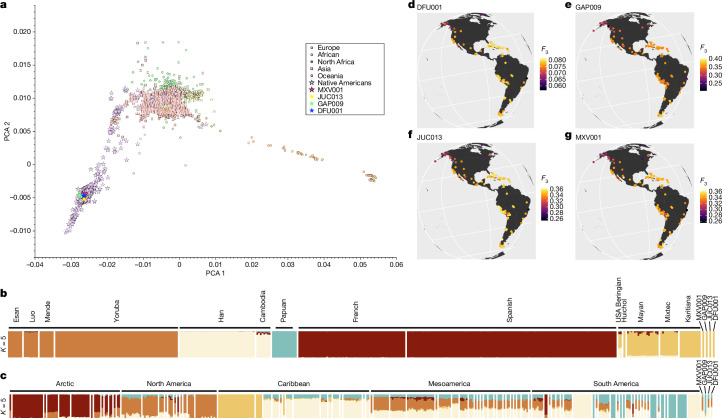
Human genomic analyses. Analyses are based on the individuals presented here and datasets listed in Supplementary Table 27. **a**, Principal component analysis (PCA) showing the position of the four individuals analysed among other Indigenous American populations. **b**, ADMIXTURE analyses (*K* = 5) for non-Indigenous American sources plus selected representative Indigenous groups from North, Central and South America. **c**, ADMIXTURE analyses (*K* = 5) for representative Indigenous groups from North, Central and South America. **d**–**g**, *F*_*3*_-statistics of the form *F*_*3*_(outgroup; target, DFU001) (**d**), *F*_*3*_(outgroup; target, GAP009) (**e**), *F*_*3*_(outgroup; target, JUC013) (**f**) and *F*_*3*_(outgroup; target, MXV001) (**g**) for Indigenous populations of the Americas. Lighter colours represent higher genetic affinity.

To explore evolutionary history of the disease complex, radiometric ^14^C dates for each individual were considered (Supplementary Information, section 4). At 2*σ* confidence level, these results securely place JUC013, DFU001 and RAZ007 in pre-1492 contexts. A modelled ^14^C age for GAP009 that accommodates an influence of marine protein consumption yielded a pre-Columbian estimated date range that spans into the late fifteenth century. By contrast, the age of MXV is estimated somewhere within the fifteenth to seventeenth centuries. Analyses of human uniparental markers (mitochondrial haplogroups C1b1 (MXV001), A2 + (64) (JUC013, DFU001 (A2bc) and RAZ007) and C (GAP009); Y-chromosome haplogroups Q1b1a1a1e1a (Q-CTS10359; MXV001), Q1b1a1a1 (Q-M3; DFU001) and Q1b1a1a1 (Q-CTS2610; JUC013)) and whole-genomic human DNA for JUC013, GAP009, DFU001 and MXV001 (Fig. 4 and Supplementary Table 27), reveal them to be genetically of Native American origin, with no indications of non-Indigenous admixture. Human DNA recovery in individual RAZ007 was too low for whole-genomic evaluation.

**Fig. 5:**
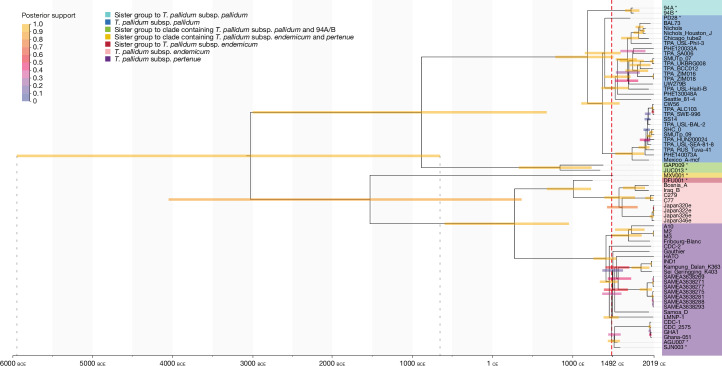
MCC tree. The maximum clade credibility (MCC) tree was generated using BEAST 2 under an uncorrelated relaxed clock model based on 2,104 SNPs with fourfold read support and removal of positions with more than 25% missing data in the dataset after exclusion of putative recombinant positions. Coloured bars represent the 95% HPD intervals of dates assigned to each node, with colour indicating the level of posterior support. The red dashed line indicates the year 1492 ce. Asterisks indicate ancient genomes.

Ancient genome representation exceeding fourfold average coverage (*n* = 9) provides multiple calibration points for molecular dating across the entire *T. pallidum* phylogeny. Radiometric data (bounded by 2*σ* intervals) or historical data from ancient genomes, along with isolation dates of modern strains, were used with the filtered SNP alignment for a molecular dating analysis using BEAST 2.6.3^38^ (Supplementary Information, section 12 and Supplementary Table 28). Beforehand, the presence of significant temporal signal in our dataset was confirmed using root-to-tip regression with Clockor2^39^ (*R*^2^ = 0.25; Supplementary Fig. 18) as well as Bayesian evaluation of temporal signal (BETS^40^; log Bayes factor (BF) = 11.1; Supplementary Information, section 12). The Clockor2 analysis suggested similar rates for TPA and TPE/TEN clades (Supplementary Fig. 18). Clock model comparison using path sampling^41^ showed that an uncorrelated relaxed clock model was largely favoured over a strict clock model based on the marginal likelihood comparison in path sampling^41^ (log BF = 167; Supplementary Information, section 12). The uncorrelated relaxed clock model was therefore used and yielded an estimated mean clock rate of 9.3 × 10^−^^8^ substitutions per site per year (95% highest probability density (HPD): 5.9 × 10^−8^ to 1.3 × 10^−^^7^ substitutions per site per year), and a time to the most recent common ancestor (*T*_MRCA_) estimate for all *T. pallidum* of 5048 bp (95% HPD: 7970–2669 bp) (Fig. 5, Supplementary Table 29 and Supplementary Fig. 19). Rates for the lineages giving rise to genomes MXV001 and GAP009 appeared higher compared with the average, with mean estimates of 1.1 × 10^−^^7^ and 3.1 × 10^−^^7^ substitutions per site per year, respectively under this clock model. The use of an alternative epoch model that accounts for potential clock rate time dependency^42^ (but not inter-lineage rate variation as offered by the relaxed clock) indicated a detectable negative correlation between time and evolutionary rate (slope coefficient 95% HPD: −0.26 to −0.01) and yielded an older *T*_MRCA_ estimate of 6296 bp (95% HPD: 8758–4179 bp, Supplementary Table 29 and Supplementary Figs. 20 and 21). Resolution of current dating uncertainties in deep temporal estimates may come from eventual analyses of ancient genomes that pre-date those presented here and the development of clock models that jointly accommodate multiple types of rate variation.

## An American origin for *T. pallidum*

This study confirms the presence in the Americas of deeply derived lineages for all three known *T. pallidum* subspecies prior to the first Columbian expedition in 1492. Consideration of these broad data in two different molecular dating approaches has yielded an upper bound of around 9000 bp for emergence of the most recent ancestor common to all *T. pallidum*. This post-dates both current genetic estimates for the initial divergence of Indigenous American populations from East Asians^43^ and archaeological estimates for human arrival in the Americas^44^. Our data thus support a scenario in which all genomically typed ancient and modern *T. pallidum* stem from an origin in the Americas during the middle Holocene epoch, possibly as a zoonotic infection from an unidentified host. Alternatively, post-glacial migrations of Siberian peoples into the Americas over the past 5,000 years^45^ could have introduced *T. pallidum* to the continent under the assumption of a Eurasian emergence; however, the exclusively northern movements of these small populations make it unlikely for them to have both harboured and transmitted the diversity needed to seed endemic sister lineages of both TPA and the TPE/TEN clade in the time periods and locations we report here. Palaeopathological interpretation also supports an endemic treponematosis in the Americas that predates these migrations (Supplementary Information, section 1).

The divergence estimates presented here, which derive from time-calibrated ancient genomes across all typed sublineages, provide the strongest support thus far for an American emergence of *T. pallidum*, consistent with the Columbian hypothesis. In light of this assertion, several questions persist regarding treponemal history such as: (1) the putative cases of Eurasian treponemal pathology that pre-date 1493^17^; (2) the mysterious ‘venereal leprosy’ historically described in the European Middle Ages^46^; and (3) the reported intensity of the late fifteenth and sixteenth century European epidemic that is unparalleled in modern contexts of any treponemal disease^47^. Although limited specificity in skeletal lesions have led to treponematosis being commonly referred to as ‘the great imitator’, the abundance of data supporting pre-Columbian treponemal infection outside the Americas should not be quickly dismissed in light of the temporal dating estimates presented here. This study provides additional threads in what is presumably a complex web of past infectious treponemal diversity both within and outside the currently typed *T. pallidum* subspecies that yet await discovery. The potential effect of uncharacterized treponemal diversity is, however, not limited to Eurasia. The Americas house a higher abundance of morphologically diagnosed archaeological cases of treponematosis, and extinct forms could account for noted pre- and post-colonial epidemiological differences as inferred from analyses of osteological collections from North American contexts^48^. Such unknown lineages may also partially account for the unexpectedly long branches we observe in genomes GAP009 and MXV001, given the known frequency of detectable recombination events that occur across multiple lineages of this pathogen. Although syphilis receives the greatest attention in relation to global human health, the impact and resilience of other *T. pallidum* members should not be minimized: Yaws persists in many parts of the developing world and has survived previous eradication efforts^49^. Further refinements to scenarios of evolutionary history may also be needed once a genome sequence of the elusive *T. carateum* (pinta) joins the discussion.

**Extended Data Fig. 4:**
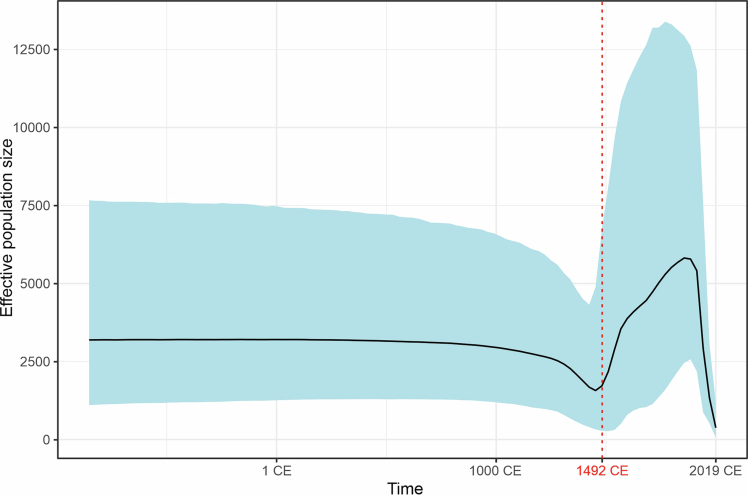
Bayesian skyline plot showing inferred effective population size (*N*_*e*_) changes of *T. pallidum* through time. The mean estimate and 95% HPD interval are shown. The red dashed line indicates the year 1492 CE.

On the basis of these new data, let us now consider where and when syphilis, the sexually transmitted disease we know today, may have first appeared. Our analyses estimate a *T*_MRCA_ of at most around 5000 bp for the divergence between the pre-contact American JUC013/GAP009 lineage and that which has given rise to all typed modern syphilis (Supplementary Table 29). With regard to syphilis sensu stricto, our analyses yield strongest support for its emergence from an ancestral form within the Americas just before the arrival of Columbus; however, analytical uncertainty in this date leaves open its possible emergence in Europe or even Africa from an American ancestral strain that was newly introduced in the late fifteenth or early sixteenth century (Supplementary Table 29). We cannot comment on the frequency or geographic distribution of this ancestral form in the Americas, and nor can we temporally place its adoption of sexual transmission, the genetic basis for which remains undetermined. Whereas our Bayesian skyline analysis indicates a possible population expansion for the pathogen in the second half of the fifteenth century (Extended Data Fig. 4), our analysis does not yield enough resolution to confidently date the divergence of the three main TPA subclades of Nichols, SS14 and Seattle as before or after 1492 ce. Despite the density of morphologically identified treponemal cases in the Americas before European arrival, few examples of possible congenital infection exist, and the observed palaeopathology is not specific to an endemic sexually transmitted treponematosis across the continents^50^. Although the reconstructed genomes presented here indicate accumulation of unique non-synonymous alleles, these changes cannot be linked to phenotypic differences in transmission strategy. In light of this, we interpret our data as indicating a high degree of treponemal variation in the Americas, which, through a series of bottlenecks and rapid population expansions during their global dissemination in the early colonial period, responded to a variety of selection pressures imposed by climate, population density, host genetics (both human and non-human) and cultural activity that favoured transmission of a small number of successful lineages. Their diffusion was subsequently enabled by human trafficking networks and European expansions, where lineages that are now globally dominant achieved their current distributions and replaced those adapted to the varied regional climates and Indigenous populations across the Americas. All five of the genomes isolated here, as well as those from historic Mexico (genomes 94A/B)^9^, may well represent diversity exclusive to the Americas that was lost in the centuries following contact.

Given the rarity of cases recovered from our screening, DNA alone may not permit reconstruction of the treponemal historical narrative to the satisfaction of all discussants. Although we anticipate tangible contributions from molecular data in the coming years, debates are likely to continue on the basis of balanced review of the available evidence.

## Methods

### Radiocarbon dating

Radiocarbon dating was carried out at the Curt-Engelhorn Centre for Archaeometry (Mannheim, Germany). Skeletal fragments were processed using ultra filtrated collagen (fraction >30kD)^55,56^ and dated using the MICADAS-AMS of the Klaus-Tschira-Archäometrie Zentrum. Radiocarbon dates are reported with the lab code MAMS.

### Stable isotope measurements and marine reservoir effects

Radiocarbon and stable isotope measurements were made on the remains from Jucusbamba, Peru (JUC013), the Chonos Archipelago of southern Chile (GAP009) and Deán Funes, Argentina (DFU001).

Prior to calibration, stable isotope measurements were made on extracted collagen from individuals where a potential marine reservoir offset could result in a radiocarbon age older than a contemporary individual who had consumed a wholly terrestrial-based diet. Data were generated from the collagen extract used for the radiocarbon date (Mannheim, Germany, laboratory code MA), and were subsequently corroborated via analysis of a second collagen extract of the same bone generated at the Max Planck Institute for Geoanthropology in Jena, Germany (laboratory code Jena). In the latter process, 1 mg of bone collagen was weighed in duplicate in tin capsules and combusted in a Thermo Scientific Flash 2000 Elemental Analyser coupled with a Thermo Delta V Advantage Mass Spectrometer at the Max Planck Institute of Geoanthropology. Isotopic values are reported as the ratio of the heavier isotope to the lighter isotope (^13^C/^12^C or ^15^N/^14^N) as δ values in parts per thousand (‰) relative to international standards (Vienna Peedee belemnite (VPDB) for δ^13^C and atmospheric N_2_ (air) for δ^15^N). Results were calibrated against international standards (International Atomic Energy Agency (IAEA)-CH-6: δ^13^C = −10.80 ± 0.47‰, IAEA-N-2: δ^15^N = 20.3 ± 0.2‰, and USGS40: δ^13^C = −26.38 ± 0.042‰, δ^15^N = 4.5 ± 0.1‰) and a laboratory standard (fish gelatin: δ^13^C ≈ −15.1‰, δ^15^N ≈ 14.3‰). Based on replicate analyses long-term machine error over a year is ±0.2‰ for δ^13^C and ± 0.2‰ for δ^15^N. Overall measurement precision was studied through the measurement of repeats of fish gelatin (*n* = 80, ±0.2‰ for δ^13^C and ± 0.2‰ for δ^15^N). Isotopic data are reported in Supplementary Table 2. All samples met quality control criteria^57,58^ with a C/N ratio between 2.9–3.6, %C of 15–48%, and %N of 5–17%.

The individual from Jucusbamba (JUC013) had stable isotopes indicative of a fully terrestrial-based C_4_ diet, thus marine offset was not considered. By contrast, the individual from the Chonos Archipelago (GAP009) had a δ^13^C value consistent with both C_4_ and marine dietary inputs (−10.5‰), but a δ^15^N value that pointed to a high marine component in the diet (+18.2‰). Chile is dominated by C_3_ plants^59^, thus a high input of marine protein in the diet likely contributed to the shift in stable isotopes. Of interest, the δ^13^C value for this individual showed more enrichment than high trophic level marine carnivores from the region, such as sea lion data from Tierra del Fuego summarized by Yesner et al.^60^. For the individual from Deán Funes (DFU001), only δ^13^C as measured on the AMS were available, which lacks the precision needed for a dietary correction. Regardless, this site is located over 600 km from the coast, so a marine dietary component is considered highly unlikely.

A modelling approach was necessary to account for the associated radiocarbon offset in individual GAP009. Because of the lack of isotopic data directly relevant for interpreting this individual (for example, measurements made on faunal samples from the same site) a literature search was made to approximate the range of expected δ^13^C and δ^15^N values for both a wholly terrestrial and marine diet. The δ^13^C range for a terrestrial diet is estimated to be −22.5 to −19.5‰ and −16 to −12‰ for a marine diet, while the δ^15^N was set to +7–11‰ and +14–22‰, respectively. These ranges are estimated from the archaeological faunal data for Tierra del Fuego and summarized Yesner et al.^60^ and the Late Pleistocene stable isotope data from horse (*Equus andium*) and llama (*Hemiauchenia paradoxa*) from northwestern Chilean Patagonia^61^. The date correction was made following the linear interpolation method^62,63^ with δ^13^C endmembers of −20 and −12‰ (100% Terrestrial and Marine, respectively) and δ^15^N endmembers of +10 and +22‰ (100% terrestrial and marine, respectively). Undertaking the interpolation using both isotopes allowed for a check on the overall sensitivity of the dating to this correction. This is especially important as: (1) there were no isotope data on associated faunal material to produce a temporally and spatially relevant bioavailable baseline; and (2) the δ^13^C values are enriched beyond expectation for a diet consisting entirely of marine protein. The result of the modelling estimates either a 100% or 85% marine diet, when using the δ^13^C and δ^15^N values, respectively.

Since all three individuals (GAP009, JUC013 and DFU001) lived in the Southern Hemisphere, the SHCal20 calibration curve of Hogg et al.^52^ was used to calibrate the radiocarbon results to a calendrical scale using OxCal v4.4^51^.

Jucusbamba, Peru lies just west of the Intertropical Convergence Zone (ITCZ), a mixing zone indicated by Marsh et al.^64^. As such, a second calibration was done with a 50/50 mix between the Northern Hemisphere IntCal20^53^ and SHCal20, with 1*σ* error of 25%, as a relatively vague approximation of a mixed calibration so that the effect could be assessed.

With no modelling for ITCZ mixing, the calibrated date is bimodal with ranges of 1308–1363 ce (73.7% probability) and 1380–1399 ce (21.7% probability). The modelled calibration is also bimodal and slightly earlier, with ranges of 1296–1328 ce (40.0% probability) and 1342–1394 ce (55.5% probability).

For the individual from the Chonos Archipelago in Chile, the two calibrations mixed the SHCal20 and Marine20 calibration curves at 85 ± 10% and 100 ± 10%, respectively. The Marine20 also had a local ΔR correction of −93 ± 30 years made, which is a weighted average of the eight nearest points in the Calib.org Marine20 database (http://calib.org/marine/). The 85% modelled correction calibrates to 1226–1451 ce (95.4% probability), and the 100% modelled correction calibrates to 1255–1483 ce (95.4% probability).

Finally, Deán Funes is well within the area designated by Marsh et al.^64^ as requiring calibration using SHCal20. Therefore, no modelling or other correction was necessary, and so only SHCal20 was used for calibration. This individual died in 1226–1281 ce (95.4% probability).

Additional numerical data for the above can be found in Supplementary Table 2. Modelled dates are shown in Fig. 1g.

### DNA extraction, library preparation and sequencing

Processing of archaeological tissues was performed in the ancient DNA clean room facility of the Max Planck Institute for Evolutionary Anthropology. Pulverised material from bone was generated with a dental drill at low rotation and high torque. For the immature molar of JUC013, powder was drilled through entry to the crown via the unfused roots to generate two aliquots of approximately 50 mg for dentin each for extraction. Subsampling of Argentinian and Chilean tissues was done on site (by D.A.R., R.B. and T.v.H.), and material was subsequently processed in the clean room facility. The collection of biological material from Chile included soft tissues, which were dissolved directly in a protein kinase digestion buffer. For individual GAP009 specifically, powder was obtained both at the site of the subperiosteal lesion and at a location adjacent to the lesion. Individual MXV001 was sampled at multiple skeletal elements in locations that displayed pathology consistent with treponemal infection. Individual RAZ007 was sampled at the occipital bone where lesions were displayed, and at the distal diaphysis of the left femur. Finally, individual DFU001 was sampled from the left iliac crest where lesions attributable to unspecific infections were observed. DNA was extracted with a silica-based method optimized for the recovery of short DNA fragments^65^. In brief, lysates were prepared by adding 1–1.8 ml extraction buffer (0.45 M EDTA, pH 8.0, 0.25 mg ml^−1^ proteinase K, 0.05% Tween-20) to the sample material in 2.0-ml Eppendorf Lo-Bind tubes^65,66^ and rotating the tubes at 37 °C for approximately 16 h. For GAP009, DFU001 and RAZ007 this lysate was used directly for library preparation. For MXV001 powders and JUC013, the lysate was further purified and concentrated over a silica membrane^65^. A double-stranded DNA library was manually generated from JUC013 with 10 µl of extract^28^, which later produced the shotgun screening library JUC013.A0101. Libraries with partial UDG treatment^67^ were manufactured for the MXV001 DNA isolate using 30 µl of extract. Lysates of 150μl-aliquots from both GAP009 digests were purified via an automated liquid handling system (Bravo NGS Workstation B, Agilent Technologies) with silica-coated magnetic beads and binding buffer D as described in Rohland et al.^66^. Elution volume was 30 µl. Single-stranded libraries of RAZ007, DFU001 and GAP009 lysate from the lesion (GAP009.A0101) and the region adjacent to it (GAP009.A0201), as well as subsequent libraries from 30 µl of the JUC013 extract (JUC013.0102) and additional powder (JUC013.A0201; extracted as above for GAP009) were manufactured via automation. DNA libraries were prepared from 30 µl extract using an automated version of single-stranded DNA library preparation^68^ described in detail in Gansauge et al.^29^. *Escherichia coli* UDG and *E. coli* endonuclease VIII were added during library preparation (together with the phosphatase) to remove uracils (either partially or in full) in the interior of molecules was applied for generation of libraries GAP009.A0301, GAP009.A0401, GAP009.A0501, JUC013.A0102 and JUC013.A0201. Negative controls were produced in parallel for all extractions and libraries. Library yields and efficiency of library preparation were determined using two quantitative PCR assays^29^.

### Computational screening for *T. pallidum* DNA

Sequencing data from GAP009 (two libraries), MXV001 (three libraries), DFU001 (one library) and RAZ007 (two libraries) was screened via HOPS^30^ using a MALT^69^ database consisting of all ‘complete’ or ‘chromosome’ level bacterial genomes (on 3 November 2017), ‘complete’ viral genomes (without ‘other’ or ‘unknown’ in their names) (on 30 October 2017) and a selection of eukaryotic genomes obtained through the NCBI assembly portal. Read processing, removal of human reads, as well as HOPS analysis to asses MALT output, was performed through the nextflow (nf)-core/eager v2.4.4^32^ pipeline, built on the nextflow v21.04.1^70^ workflow language. nf-core/eager is a reproducible pipeline for quality control and assessment of sequencing data, generating as well as analysing mapping data and generating SNP calls, among other features. The nf-core/eager pipline is optimized for aDNA and employs several gold standard published software packages including AdapterRemoval v2^71^ for removal of adapters and trimming of low-confidence base calls at the 3′ end of reads, bwa v0.7.17^72^ for mapping, samtools c1.12^73^ for the removal of reads with low mapping quality (<37), dedup v0.21.8 for removal of duplicates, and DamageProfiler^74^ for analysing damage percentages in reads, among others. A full list of the software used by this nf-core/eager pipeline can be found in Supplementary Table 3. Candidates were assessed based on their HOPS candidate profiles (Supplementary Fig. 10). JUC013 was identified as a *T. pallidum* candidate via an alternative computational approach to the one described above (Supplementary Information, section 3). The number of reads from RAZ007 that were assigned to the genus *Treponema* in MALT (*n* = 3 for the femur and *n* = 2 for the occipital) were too low to permit an assessment in HOPS.

### Enrichment for *T. pallidum* DNA and sequencing

Sample and control libraries were enriched for TPA DNA in two rounds of consecutive in-solution capture^75^ automated on the Bravo NGS workstation. Both non-enriched and enriched library products were sequenced on an Illumina HiSeq 4000 using single-end 75 bp chemistry.

### Analyses of *T. pallidum* capture data

See Supplementary Information, sections 6–8.

### Human ancestry analyses

We analysed the shotgun and human-enriched capture data using nf-core/eager v. 2.3.4^32^. AdapterRemoval v.2^71^ was used to trim adapter sequences and to remove adapter dimers and low-quality sequence reads (min length = 30; min base quality = 20). Pre-processed sequences were mapped to the human genome assembly GRCh37 (hg19) from the Genome Reference Consortium^76^ using BWA v. 0.7.12^72^ and a seed length of 32. The C to T misincorporation frequencies typical of aDNA were obtained using mapDamage 2.0^77^ to assess the authenticity of the ancient DNA fragments. Genetic sex of the analysed individuals was assigned using SNP capture data by calculating the ratio of average X chromosomal and Y chromosomal coverage to the average autosomal coverage normalized by the chromosome length at the targeted SNPs^78^. Samples with an X rate between 0.35 and 0.55 and a Y rate between 0.4 and 0.7 were confirmed male (JUC013, DFU001, MXV001 and RAZ007). ANGSD v. 0.935 (as implemented in nf-core/eager) was used to estimate nuclear contamination, since males are expected to be homozygous at each X chromosome position^79^. The contamination estimates returned a value of 0.0 ± 0.0 for all individuals analysed. We used samtools mpileup (parameters –q 30 –Q 30 –B) to generate a pileup file from the merged sequence data of each individual, and used a custom script (pileupCaller v. 8.2.2^80^) to genotype the individuals, using a pseudo-haploid random draw approach. Only individuals with >10,000 SNPs called (GAP009: 156,533; JUC013: 761,151; MXV001: 1,027,622; DFU001: 43,818 (on the Human Origins panel)) were retained for further population genetics analyses (RAZ007 was excluded due to a lower number of SNPs called, 2,134).

Consensus sequences for the mitochondrial genomes of the individuals were determined via the mapping of the sequencing reads obtained from shotgun and post-capture enrichment (post-capture enrichment only for MXV001) to the revised Cambridge reference sequence^81^. The mitochondrial DNA (mtDNA) sequences were aligned and manually inspected with Geneious Prime 2021.1.1 (https://www.geneious.com). For the resulting sequences, we filtered positions with likelihoods above 30 and used HaploGrep2^82^ and HAPLOFIND^83^ to assign and confirm the corresponding mtDNA haplogroups. The aligned mtDNA genomes were compared to other available genomes from the literature. A new contamination estimation was performed after the processing of the human data, this time using the mtDNA information and the Haplocheck–Contamination Detection v1.0.0 from Mitoverse^84^. The results obtained for the five individuals at the contamination status and contamination level are non-detectable except for RAZ007 (average coverage on the mtDNA reference: 549X (MXV001), 1252X (DFU001), 721X (GAP009), 53X (JUC013) and 224 (RAZ007, contamination level 4.5%)). For the Y-haplogroup assignments, we created pileups of reads for each individual which mapped to Y-chromosome SNPs as listed on the ISOGG Y-DNA Haplogroup Tree (v15.73; https://isogg.org/tree/). We then manually assigned Y-chromosome haplogroups for each individual based on the most downstream SNP retrieved after evaluating the presence of upstream mutations along the Y-chromosome haplogroup phylogeny as described in Rohrlach et al.^85^.

We merged sequence data for JUC013, MXV001, DFU001 and GAP009 with sources of published genomic data summarized in Supplementary Table 27. A principal component analysis (PCA) from this worldwide reference set comprising 7271 ancient and modern individuals (593,115 SNPs), was constructed to investigate admixture with European/African ancestry components (Fig. 4a). RAZ007 was excluded from this analysis due to its low coverage (fewer than 10,000 resolved SNPs in the 1240 K reference panel).

We used a set of ancestral populations from five continental regions to estimate admixture proportions to test for admixture with non-Native American populations (which would be a clear indication of the individual being born during the post-contact period): we included Huichol^43^, Mayan, Karitiana^86^ and Mixtec^87^ as part of a genetic pool to assess the Native American contribution to our samples; the African component was estimated by using the genetic data of individuals from Yoruba^86,88^, Esan, Mende, and Luo^78^ groups; Spanish^87,89^ and French^86,89^ were used to model the European genetic contribution; the East Asian genetic contribution consisted of Han and Cambodian^88^; and the near Oceania component was estimated with genotypes from Papuan^88^. We then used ADMIXTURE^90^ and AdmixturePlotter^91^ to calculate the best *K* for our model (that is, the one with the lowest cross validation error (CVE); *K* = 5) and the admixture proportions of the components that are maximized in each of the parental populations modelled (Fig. 4d,e). To further refine the visualization of the Native American ancestry in the individuals analysed, another ADMIXTURE run was performed, but only with Indigenous populations of the Americas as ancestral populations: populations from the Arctic, North American, Caribbean, Mesoamerican and South American regions were included as ancestral (Fig. 4f,g). The lowest CVE was estimated at *K* = 5. The results yielded admixture proportions expected for the geographical regions and archaeological periods that the contextual information provided for the individuals analysed.

To assess the genetic relationships and admixture events suggested in the ADMIXTURE analysis, we carried out *F*-statistics analyses using the Xerxes CLI software from the Poseidon framework^92^. We performed *F*_3_-tests of the form *F*_3_(outgroup; test, *X*) to measure the amount of shared genetic drift between Indigenous American test populations and each analysed individual after their divergence from an African outgroup (Fig. 4a–c), where *X* refers to each of the individuals analysed, test is each of the Indigenous American populations included in our analyses, and Outgroup corresponds to Mbuti individuals from Congo^88^.

### Virulence factor analysis

To infer putative functional differences among ancient and modern treponemal genomes, we investigated the presence or absence of genes potentially associated with virulence profiles in the *pallidum* (syphilis) and *pertenue* (yaws)/*endemicum* (bejel) lineages, as well as in their outgroup *T. paraluiscuniculi*^93–95^ (Supplementary Table 25). For this, BAM files prior to the application of a mapping quality filter with SAMtools^73^ were used to calculate the coverage across 68 chromosomal genes annotated on the Nichols reference genome (NC_021490.2) (Supplementary Table 26), as previously performed^26,96^. Coverage proportions across each investigated gene were calculated with BEDtools^97^ and subsequently plotted on a heatmap using ggplot2 of R version 4.2.2 (Extended Data Fig. 3 and Supplementary Table 26). Moreover, we used the program snpEff version 3.1i [build 2012-12-12]^98^ to infer the possible functional impact of called SNPs (see Supplementary Information, section 7.3 for details on SNP calling strategy) across eight ancient and 53 modern treponemal genomes. The inferred SNP effects were integrated into the comparative SNP table of all treponemal genomes generated for our analyses (*n* = 61) using MultiVCFAnalyzer v0.85.2^99^. The table was filtered to identify SNPs and their effects present on the three newly reported lineages, which gave rise to ancient genomes MXV001 (Supplementary Table 21), DFU001 (Supplementary Table 22), and JUC013/GAP009 (Supplementary Table 23), as well those derived in all modern TPA (syphilis) diversity (Supplementary Table 24).

### Molecular dating

To investigate the potential of a molecular dating analysis, we formally evaluated the presence of temporal signal in our dataset using two different approaches. First, we performed a root-to-tip regression, as implemented with Clockor2^39^ (Supplementary Fig. 18). This showed a positive correlation between sample date and genetic distance to the root (*R*^2^ = 0.25). Regressions fitted independently for the TPA and TPE/TEN clades suggested similar evolutionary rates in both lineages (6.7 × 10^−^^8^ and 8.4 × 10^−^^8^ substitutions per site per year, respectively; Supplementary Fig. 18), although the local clock model was slightly preferred over a global clock model (Bayesian information criterion (BIC) = −1,250 and −1,254, respectively). In a second step, we compared the fit of a strict clock and an uncorrelated log-normal relaxed clock using path sampling^41^, as implemented in the BEAST 2 model selection package^38^. In this case, an exponential coalescent tree prior was used due to known issues of the skyline coalescent for model comparison^100^. A proper uniform prior between 0 and 1 million was used for the end population size. For these analyses, the dates of ancient genomes were fixed to their median estimates. The marginal likelihood of each model was then estimated using path sampling^40^. We used 10 million pre-burn-in iterations and 100 steps initially set to 3 million iterations each (including 50% burn-in), which was then extended by the same length to ensure that the resulting Bayes factor estimate had converged. The latter showed overwhelming support for the relaxed clock model (log BF = 167). Finally, we performed Bayesian evaluation of temporal signal (BETS)^40^. The previously selected relaxed clock model was then compared to an isochronous model in which all samples were set to 0 bp and the clock rate fixed to one, with all other parameters otherwise identical. To this end, path sampling^40^ was used as described above. The results indicated strong support for the heterochronous model (log BF = 11.1) confirming the presence of significant temporal signal in our dataset.

Following the assessment of significant temporal signal in our dataset, a time-calibrated phylogenetic tree was estimated using BEAST 2.6.7^38^ based on the alignment containing 2104 SNPs described above (Fig. 3 and Supplementary Table 20). A filtered alignment was used to account for the number of constant sites remaining after all filtering steps were applied. The tree was calibrated using sample dating information. Isolation dates were used for modern strains. For ancient strains, dating information was entered as uniform prior distributions bounded by intervals based on either dietary modelled 2*σ* ^14^C date ranges, or historical data (Supplementary Table 28). A general time reversible (GTR) substitution model was used with a gamma-distributed rate heterogeneity using four discrete categories^101^, with permission for 25% missing data (ambiguous base calls) across the dataset for each position. A Bayesian skyline coalescent model with ten groups was used as the tree prior^102^ to allow estimation of changes in population size through time, and following Majander et al.^26^. Following clock model selection, an uncorrelated log-normal relaxed clock model was used^103^ (Supplementary Data, BEAST2_UCLN.xml), with uniform prior between 0 and 1 substitutions per site per year for the mean rate. All other parameters were set to default (BEAUti v.2.6.7)^38^. The analysis was run using an MCMC of 300 million iterations, with sampling every 30,000 steps. Ten percent of the iterations were discarded as burn-in and convergence was assessed in Tracer v. 1.7.1^104^, ensuring that all effective sample size (ESS) values were above 200. We also sampled the prior distribution of parameters using the same MCMC specifications to ensure that clock rate and *T*_MRCA_ estimates were driven by the data (Supplementary Fig. 19). A maximum clade credibility (MCC; Fig. 4) tree was constructed from the posterior distribution of trees using Treeannotator^105^. Sampled trees and population parameters were used to reconstruct a Bayesian skyline plot (Extended Data Fig. 4) using a dedicated R script to visualize estimates of bacterial population size through time.

In addition, to account for the fact that observed evolutionary rates may vary depending on the considered time-scale, we performed a second analysis using the time-dependent-rate (TDR) model implemented in BEAST 1.10.4^42,106^ (Supplementary Data, BEAST1_TDR.xml). The latter model can, however, not accommodate inter-lineage rate variation as the uncorrelated relaxed clock model does. We used a three-epoch model with an exponential structure including a transition first at 100 years ago and a second at 1,000 years ago. The midpoint of each period was used as a reference point (taking 5,000 years ago for the last epoch). We used normal priors with mean = −14.5 and 0, and s.d. = 5 and 5 for the intercept and slope coefficients of the log-linear relationship between the substitution rate and time, respectively (corresponding to an average of around 5.10–7 substitutions per site per year at time 0 and no prior assumption on the direction of the relationship). Other model specifications were identical to the main analysis described above (Supplementary Fig. 20). Results of the dating analyses are presented in Supplementary Table 29.

### Reporting summary

Further information on research design is available in the Nature Portfolio Reporting Summary linked to this article.

## Online content

Any methods, additional references, Nature Portfolio reporting summaries, source data, extended data, supplementary information, acknowledgements, peer review information; details of author contributions and competing interests; and statements of data and code availability are available at 10.1038/s41586-024-08515-5.

## Supplementary information

Supplementary Information This document includes Supplementary Methods, Supplementary Figs. 1–21, Supplementary Tables 3 and 11–14, additional references, and a list of references cited in the Supplementary Tables.

Reporting Summary

Supplementary Tables Supplementary Tables 1, 2, 4–10 and 15–29

Supplementary Data Molecular dating files (.xml) for use within BEAST. BEAST1_TDR.xml – “BEAST1 Time Dependent Rate”. BESAT2_UCLN.xml – “BEAST2 Relaxed Clock”.

## Data Availability

Data are accessible via ENA Project ID PRJEB62879. Data from other public sources utilized in this study can be accessed via the information contained in Supplementary Table 8 (*T. pallidum* data) or Supplementary Table 27 (human data). Mapping to the human genome was performed against reference GCF_000001405.13. Map figures were generated as follows: ‘America’ was downloaded from: https://web.archive.org/web/20180625082054/https://tapiquen-sig.jimdo.com/descargas-gratuitas/américa/. Carlos EfraÌn Porto TapiquÈn. OrogÈnesis Soluciones Geogr·ficas. Porlamar, Venezuela 2015. Based on shapes from Enviromental Systems Research Institute (ESRI). Free Distribuition.

## Code availability

BEAST 1 and BEAST2.xml are included as supplementary files. The custom code used for the analysis of SNP effects and virulence-gene coverage is available at: https://github.com/mariaspyrou/Treponema-Americas-paper/tree/main/virulence-gene-analysis and https://github.com/mariaspyrou/Treponema-Americas-paper/tree/main/snpeff.
