# Supplementary material for: Ancient genomes reveal a deep history of Treponema pallidum in the Americas
Source: Nature. 2024 Dec 18;640(8057):186–93. doi: 10.1038/s41586-024-08515-5 (PMC11964931; doi:10.1038/s41586-024-08515-5)
Supplement: Supplementary file 1 — This document includes Supplementary Methods, Supplementary Figs. 1–21, Supplementary Tables 3 and 11–14, additional references, and a list of references cited in the Supplementary Tables. [file 41586_2024_8515_MOESM1_ESM.docx]

**SUPPLEMENTARY INFORMATION for: “Ancient genomes reveal a deep history of *Treponema pallidum* in the Americas”**

This document includes supplementary methods, Supplementary Figures S1-S21, and Supplementary Tables S3 and S11-14

Rodrigo Barquera^1^, T. Lesley Sitter^1^, Casey L. Kirkpatrick^1,2^, Darío A. Ramirez^3,4^, Arthur Kocher^1^, Maria A. Spyrou^1,5^, Lourdes R. Couoh^6^, Jorge A. Talavera-González^6^, Mario Castro^7,8^, Tanya von Hunnius^9^, Evelyn K. Guevara^10^, W. Derek Hamilton^11^, Patrick Roberts^12^, Erin Scott^13^, Mariana Fabra^3,4^, Gabriela V. Da Peña^14,15^, Aryel Pacheco^16^, Mónica Rodriguez^17^, Eugenio Aspillaga^18^, Anthi Tiliakou^1^, Elizabeth A. Nelson^1^, Karen L. Giffin^1^, Raffaela A. Bianco^1^, Adam B. Rohrlach^1,19,20^, María de los Ángeles García Martínez^21^, Fabiola A. Ballesteros Solís^6^, Antti Sajantila^10,22^, Shelley R. Saunders^9^, Rodrigo Nores^3,4^, Alexander Herbig^1^, Johannes Krause^1^, and Kirsten I. Bos^1^

^1^Max Planck Institute for Evolutionary Anthropology, Leipzig, Germany

^2^Western University, London, Ontario, Canada

^3^Instituto de Antropología de Córdoba, Consejo Nacional de Investigaciones Científicas y Técnicas, Universidad Nacional de Córdoba, Museo de Antropologías, Córdoba, Argentina

^4^Departamento de Antropología, Facultad de Filosofía y Humanidades, Universidad Nacional de Córdoba, Córdoba, Argentina

^5^Institute for Archaeological Sciences, Eberhard Karls Universität Tübingen, Tübingen, Germany

^6^ Dirección de Antropología Física, Instituto Nacional de Antropología e Historia, Mexico City, Mexico

^7^Museo Nacional de Historia Natural, Santiago, Chile

^8^Department of Morphology, Faculty of Medicine, Clínica Alemana-Universidad del Desarrollo, Santiago, Chile

^9^McMaster University, Department of Anthropology, Hamilton, Ontario, Canada

^10^Department of Forensic Medicine, University of Helsinki, Helsinki, Finland

^11^Scottish Universities Environmental Research Centre, East Kilbride, UK

^12^Department of Coevolution of Land Use and Urbanisation, Max Planck Institute for Geoanthropology, Jena, Germany

^13^Laboratory Unit, Max Planck Institute of Geoanthropology, Jena, Germany

^14^Consejo Nacional de Investigaciones Científicas y Técnicas, Museo de Ciencias Naturales y Antropológicas Juan C. Moyano, Mendoza, Argentina

^15^Departamento de Arqueología, Facultad de Filosofía y Letras, Universidad Nacional de Cuyo, Mendoza, Argentina

^16^Bioarchaeology Research Group, Durham University, Durham United Kingdom

^17^Independent researcher, Santiago, Chile

^18^Department of Anthropology, University of Chile, Santiago, Chile

^19^School of Computer and Mathematical Sciences, University of Adelaide, Adelaide 5005, South Australia, Australia

^20^Adelaide Data Science Centre, University of Adelaide, Adelaide 5005, South Australia, Australia

^21^Dirección de Salvamenta Arqueológico, Instituto Nacional de Antropología e Historia, Mexico City, Mexico

^22^Forensic Medicine Unit, Finnish Institute of Health and Welfare, Helsinki, Finland

**Table of Contents**

1. **Overview of treponemal palaeopathology in the Americas**…………………………………… **3**
2. **Archaeological details and skeletal assessments**……………………………………………………..**6**
   1. Chonos Archipelago, Chile…….…………………………………………………………………………….6
   2. Northern Mexico City, Mexico….…………………………………………………………………………8
   3. Jucusbamba, Peru..…………….……………………………………………………………………………..10
   4. Deán Funes, Argentina………………………………………………………………………………………11
   5. STC Línea 6 Metro Refinería Azcapotzalco, Mexico City, Mexico……………………….13
3. **Computational screening for *T. pallidum* in existing datasets**……………………………….**15**

3.1 Sample database……………………………………………………………………………………………….15

3.2 Performing taxonomic identification reads using MALT…………………………………….17

3.3 Identifying *Treponema pallidum pallidum* candidate samples from MALT…………19

1. **Radiocarbon dating**…………………………………………………………………………………………….....**19**

4.1 MXV001………………………..……..…………………………………………………………………………..20

4.2 RAZ007 …………………………………………………………………………………………………………….21

1. **Computational screening of GAP009, MXV001 and DFU001.**…………………………………**22**
2. **Analyses of *T. pallidum pallidum* capture data**………………………………………….…………..**24**

6.1 Capture validation through reference mapping…………………………………………………24

6.2 Competitive mapping………………………………………………………………………………………..24

6.3 SNP calling…………………………………………………………………………………………………………25

6.4 Analyses of genome KM14-7……………………………………………………………………………..26

1. **SNP filtering**……………………………………………………………………………………………………………**30**

7.1 Low complexity regions…………………………………………………………………………………….30

7.2 Highly conserved genomic regions…………………………………………………………………….30

7.3 Recombinant positions……………………………………………………………………………………..31

7.4 Regions prone to non-target mapping………………………………………………………………31

7.5 Masking of spurious regions from genotyping…………………….…………………………….33

1. **Phylogenetic assessment of *T. pallidum* genomes**..……………………………………………….**36**
2. **Molecular dating**…………………………………………………………………………………………………….**40**

**References** …………………………………………………………………………………………………………….**44**

**1.** **Brief Overview of treponemal paleopathology in the Americas**

Correspondence to: casey_kirkpatrick@eva.mpg.de

Syphilis, yaws, and bejel are among the few infectious diseases that cause changes in the skeleton, leaving “a record which outlives death, and is as imperishable as the bony skeleton itself”^1^. Cases of treponematosis can sometimes be identified based on this osteological evidence; however, there are few osteological indicators that are pathognomonic to treponematosis and these are generally only apparent in the late stages of the disease. Upon suspicion of treponemal disease, many attempts have been (and continue to be) made to distinguish between the treponemal diseases based on outdated understandings of the osteological evidence, as well as the geographic, climatic, social, and demographic context of the burial. However, Steinbock^2^ found that differences in the osteological effects of syphilis, yaws, and bejel are merely quantitative. Baker^3^ also points out that specifying a modern form of treponemal infection ignores the long, unknown evolutionary history of treponemal diseases and how they may have affected humans differently in the past. Consequently, when evidence of treponemal disease can be identified in human remains, paleopathologists are generally advised to diagnose only the broader category of treponematosis, rather than syphilis, yaws, or bejel. This poses a particular challenge when trying to trace the origins of the specific diseases.

Although the limited pre-Columbian evidence of treponematosis in Afroeurasia has been widely debated, secure skeletal evidence of treponematosis has long been observed in human remains from the Americas. In 1871, J. Wyman crafted the earliest description of treponematosis from an archaeological context in North America^4^. This was later followed by reports from South and Central America, by Parrot^5^ and Gann^6^, respectively. Since the publication of these seminal works, an abundance of American evidence of treponematosis has been revealed, firmly establishing its presence before contact.

The earliest alleged case of treponematosis in the Americas comes from the Chan Hol 3 underwater cave site in Mexico’s Yucatan peninsula and dates to at least 9900 +/- 100 YBP^7^. The individual is incorrectly reported to be infected with “*Treponema peritonitis”*, a misinterpretation of the treponemal periostitis described by Gerszten and colleagues^8^ with which the authors compare this case. However, the location, pattern, and morphology of the lesions observed on the Chan Hol 3 cranium are inconsistent with a diagnosis of treponematosis and are more likely a result of taphonomic erosion. The authors also argue that the infection may have resulted from peri-mortem trauma to the skull, which is in disagreement with the transmission patterns and pathophysiology of treponemal diseases as they do not typically affect the skeleton until the secondary or tertiary stages of the disease, with osteological involvement typically occurring years after the initial infection.

Oliveira and colleagues ^9^ presented a possible case of treponematosis dating to at least 9400 YBP from the Lapa do Santo cave site in Brazil. The authors attribute the observed skeletal and dental abnormalities in the 5-year-old child to congenital syphilis. This individual undoubtedly presents evidence of disease in the form of stunted growth, *cribra orbitalia*, porosity around the nasal suture, and a fistular lesion on the temporal bone; however, none of these conditions are pathognomonic to congenital syphilis or to treponematosis more generally. The widespread hypoplasia and carious lesions throughout the deciduous and permanent teeth may also be more indicative of amelogenesis imperfecta than congenital syphilis^10^. Amelogenesis imperfecta has also been associated with peg-shaped lateral incisors and other dental morphological abnormalities such as those observed in this case (though the depicted incisors appear to have normal morphology and separation of mamelons in the growing incisors rather than being tapered and notched Hutchinson’s incisors, as claimed). Oliveira and colleagues^9^ acknowledge that much of the remaining evidence presented in support of the diagnosis of congenital syphilis is debatable as the alleged osteolytic lesions in the skull, left ulna, and left femur may be a result of taphonomic processes.

Gerszten and colleagues^8^ also stated that evidence of treponematosis can be found in human remains in nearly every Andean culture from Chile and Peru dating back almost 8000 years. Unfortunately, no further details about these early cases or their diagnostic criteria were provided; therefore, this claim cannot be verified.

Secure diagnostic evidence of treponematosis becomes prevalent in the archaeological record after 8000 YBP, during the Middle Archaic Period in the modern regions of Canada and the USA. After 3000 YBP (around the time of the Neolithic Revolution in the Americas) the prevalence increases significantly, perhaps due to wider transmission linked with increasing population size and density, better preservation of later burials, and more archaeological interest in this time period^11^. It is during this period of growth that the first secure osteological evidence of treponematosis appears in the modern region of Mexico (ca. 900 – 200 BCE), though it may have been present before this time^12,13^. The prevalence of treponematosis continues to grow throughout North America until the time of contact, with the same types of lesions observed throughout North America regardless of chronological context^11^. The prevalence of osteological evidence of treponematosis drastically decreases in the colonial period. This may be an effect of the “osteological paradox”^14^ as historical records indicate that the strain of syphilis responsible for the 15^th^ century epidemic killed victims relatively quickly, perhaps prior to osteological involvement^15^. Alternatively, osteological evidence of treponematosis may have suddenly declined due to the introduction of novel infectious diseases from Europe. These diseases decimated indigenous populations in the Americas and would have reduced the number of individuals who lived long enough to suffer from the late stages of treponematosis in which the bones become involved.

Evidence of pre-contact treponematosis in Central and South America is decidedly less than that seen in North America. Given that most of the early evidence of treponematosis in South America has been found in the western desert coast of Peru and Chile, it is possible that the dearth of treponemal evidence is largely due to the relative paucity of scholarly focus on treponematosis in other areas, and issues of preservation in more humid climates. Nevertheless, the number of published cases has increased significantly in recent decades with pre-contact treponematosis reported in most countries in both Central and South America, and some cases also reported from the Caribbean Islands. Without accounting for the strength of diagnosis, the earliest reported case of treponematosis in Central America was discovered in Belize and dates to 1200-900 BCE^16^, while the earliest case in the Caribbean Islands comes from Cuba and dates to 3000 BCE^17^. The earliest cases in South America were discovered in Chile (5860 – 1720 BCE)^18^, Brazil (5367-5211 BCE)^19^, Peru (ca. 5000 BCE)^20^, and Colombia (3120-3040 BCE)^21^. Unfortunately, due to the relative paucity of published evidence of treponematosis and a lack of large-scale surveys of this evidence in the Caribbean Islands, Central America, and South America, any statements regarding fluctuations in the prevalence of treponematosis, or the directionality of its spread in these regions, would be merely speculative.

Some scholars have noted that the congenital form of treponematosis is much more likely to be associated with venereal syphilis than yaws or bejel. This is due to the fact that endemic treponematosis usually infects children and is in a latent state at child-bearing age, while venereal syphilis is more likely to initially infect mothers at a child-bearing age^11^. Only two debatably pathognomonic cases of congenital treponematosis have been reported from the Americas (specifically North America) before the time of contact, though a number of less secure cases have been reported throughout the Americas^11,22^. Due to the scarcity of secure cases of pre-contact congenital treponematosis and the question of whether syphilis in the sexually transmitted form was actually present in the pre-colonial Americas, some scholars have argued that the two aforementioned cases might be better interpreted as the less common congenital yaws^22^. However, it must also be noted that there may be other reasons for the low number of pre-contact cases of congenital treponematosis. For example, syphilitic pregnancies often end in spontaneous abortion or death of the infant soon after birth^23^. It is possible that the remains of these children were not buried in a typical manner; furthermore, the size and fragility of their bones may have rendered them more susceptible to taphonomic destruction. Consequently, these individuals may be less visible in the archaeological record.

In addition to the aforementioned congenital cases, possible diagnoses of sexually transmitted syphilis have been made for two pre-contact adults with skeletal evidence of treponematosis and thoracic aortic aneurysms. The first of these individuals dates to 515 BCE from the Bracken Cairn site in Saskatchewan, Canada^24,25^, and the second dates to 210 BCE from the Chiu Chiu site in Antofagasta, Chile^26^. These individuals have been tentatively diagnosed as having syphilis because clinical data show that thoracic aortic aneurysms occur in individuals suffering from the tertiary stage of sexually transmitted syphilis, but not from the endemic treponematoses^23,27,28^. Although there is a strong association between the two conditions, thoracic aortic aneurysms are not unique to syphilis and they may appear in individuals with treponematosis coincidentally. Consequently, these diagnoses would benefit from biomolecular verification.

In conclusion, despite the wealth of paleopathological evidence for pre-contact treponematosis that has been discovered in the Americas, the existence of pre-contact syphilis has remained a matter of debate. It is for this reason that molecular paleopathology is necessary to resolve this issue.

**2. Archaeological details and skeletal assessments**

**2.1 Chonos Archipelago, Chile, GAP009**

Correspondence to: casey_kirkpatrick@eva.mpg.de

**Tibder78, Isla Benjamín 1/CH11, Chonos Archipelago, Chile**


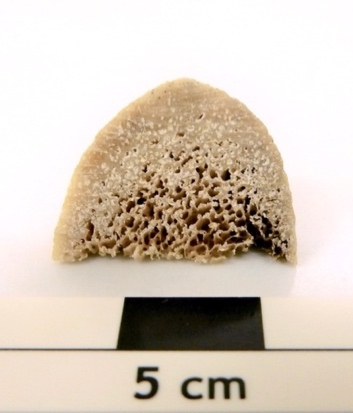

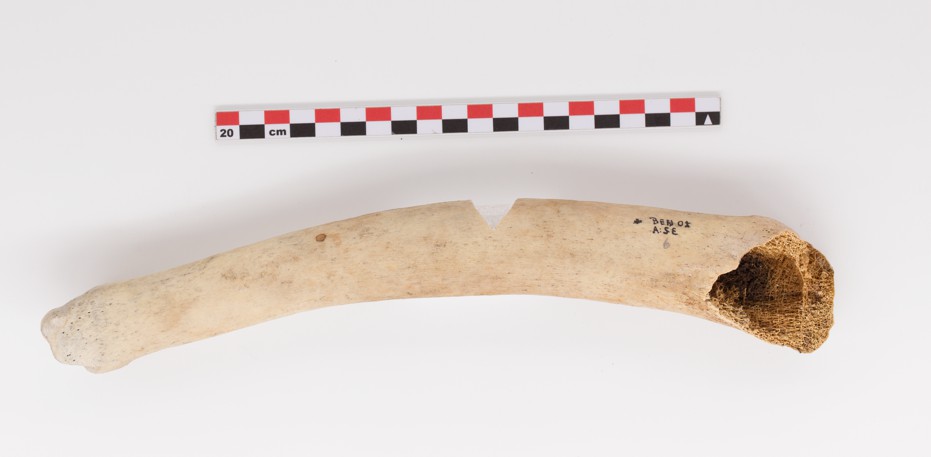

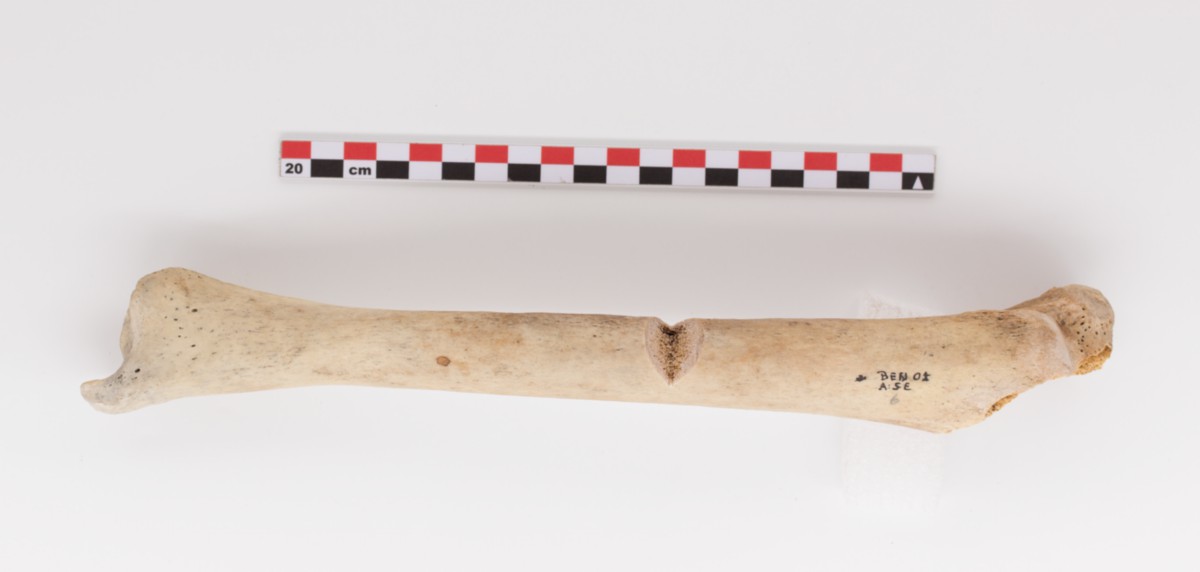


Figure S1 - Right tibia from Individual GAP009 sampled for this study: Upper left: Medial view showing the true bowing and expansion of the tibial diaphysis, Upper right: Superior view of fragment collected from the tibia in 2002 showing the trabeculated cortical bone, obliteration of the medullary canal, and a small, darker coloured subperiosteal lytic lesion on the lateral edge (right side in photo). Bottom: Anterior view showing the trabeculated cortical bone and obliteration of the medullary canal within the sectioned area, a small lytic focus distal to the sectioned area, and porosity and healing striated periostosis throughout the diaphysis.

The remains from a minimum number of 12 individuals, including the GAP009 tibia, were recovered without documentation by Ocampo and Aspillaga^29^ from the surface of the Isla Benjamin 01 cave site. Upon further investigation, Reyes et al.^30^ also recovered the remains of a minimum number of 5 individuals, as well as a variety of faunal remains (e.g. fish, birds, sea lions, marine otters, coypu, huemul). All of the collected remains are currently stored at the Universidad de Chile. The north-facing cave rests 3 meters above the current sea level and is located adjacent to a small bay accessible through a narrow channel off of the southern bank of a 20 km-long inlet that runs east-west from the western side of Isla Benjamin in the Chonos Archipelago. The cave contains a shell-midden matrix and its entrance is obscured and protected by thick forest and vegetation^31^.

Archaeological and radiocarbon dating indicate that the cave was occupied from around 1700 YBP (calibrated), and despite a lack of lithic material, biological remains provide evidence of fishing, fowling, mollusk consumption, and hunting of marine fauna as well as coypu and huemul, with the remains of the aforementioned species dating between 1400 and 1700 YBP (calibrated). Huemul is a species of deer found only on the mainland, which is 100 km away from the Isla Benjamin 01 site at its nearest point, suggesting long-distance mobility and perhaps trade by this time^31^. Radiocarbon dating of two human ribs from the surface of this site indicate that this cave site was used until at least 239 to 482 YBP (calibrated) (1468 - 1711 CE^31^). These latter dates from the human remains span the time of contact, with the first contact between the Chonos and the Spanish having occurred in 1553 CE^32^.

GAP009 is a genetically female adult with an estimated age of at least 19 years based on the complete fusion of the tibial epiphyses^33^. The sampled element is a right tibia; one of the scattered commingled remains recovered from the surface within a cave that also contained a shell midden. The medial half of the proximal end of the bone was broken post-mortem and is now missing. Similarities between the measurements of the antero-posterior and medio-lateral diameters, as well as the circumferences at the midpoint and nutrient foramen levels, indicate abnormal thickening of the middle third of the diaphysis. The anterior crest is also more rounded than usual, indicating pathological expansion of the diaphysis, which is usually a response to active disease in the medullary canal. The expanded cortex is more porous with trabeculated bone spanning the thickness of the cortical bone in some areas and expanding into, and contributing to the obliteration of, the medullary canal. The surface of the tibia has fine striations and porosity from healing periostosis, and the bone is morphologically abnormal with true anterior bowing of the diaphysis (as opposed to “saber shin” pseudo-bowing). Although true diaphyseal bowing has been associated with treponematosis (usually in congenital cases), it is not diagnostic. Although it is not visible in the photographs, this tibia also presents a latero-medial curvature; however, this is observed in most Chonos tibiae and is likely related to muscle development. Hackett^34^ considers the presence of striate expansions, as observed in this individual, to be diagnostic criteria (on trial) for syphilis. Furthermore, there is a small focal cavitation with healing margins on the anterior distal third of the diaphysis, which lends further diagnostic strength for treponematosis. Additionally, within the sectioned fragment, a small lytic lesion with slightly darker colouration was observed within the cortical bone at the lateral edge of the trabeculated area. It is from this lytic focus that the highest concentration of treponemal aDNA was isolated.

**2.2 Northern Mexico City, Mexico, MVX001**

Correspondence to: rodrigo_barquera@eva.mpg.de

**Burial 1, Manuel González No. 95**


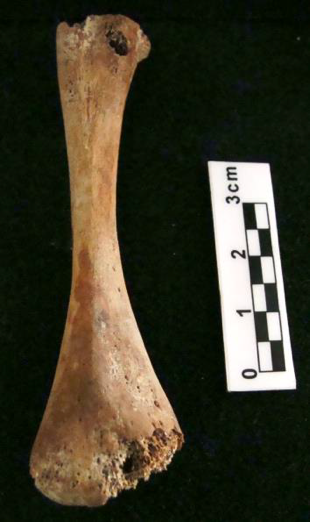

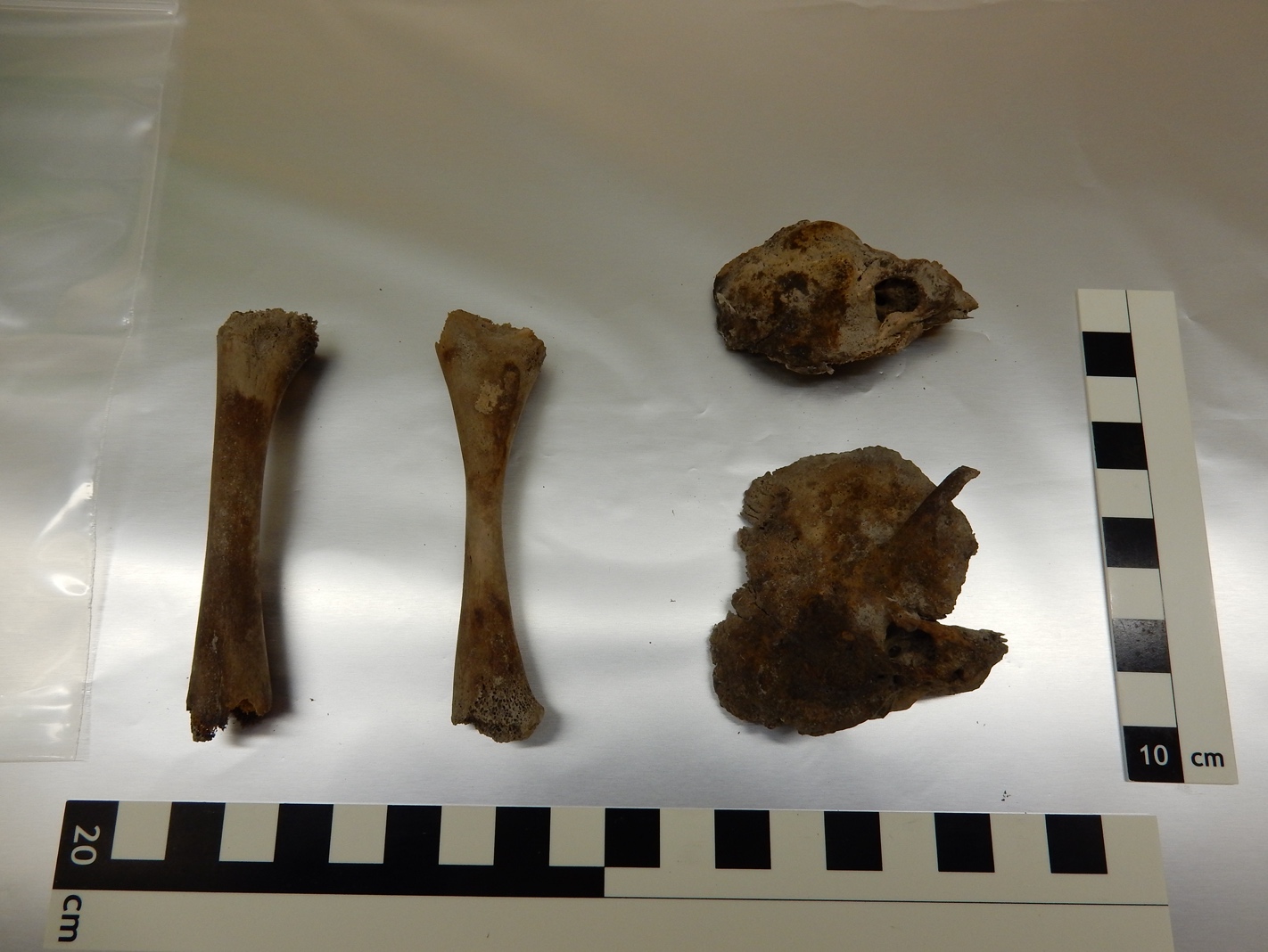


Figure S2 - Skeletal elements sampled from Individual MXV001. Left: right tibia. Right: right humerus.

The remains of this individual (burial 1; MXV001 in this paper) were recovered during the archaeological explorations of the Archival Salvage Project Manuel González No. 95, San Simón Tolnáhuac, Cuauhtémoc Delegation, Mexico City, by Dr. María de los Ángeles García Martínez, a researcher under contract to the Directorate of Archaeological Salvage of the National Institute of Anthropology and History. The archaeological work began on May 10, 2016, in rescue modality, since the demolition of the last building was being carried out and construction work would begin shortly. During the archaeological salvage research work at Manuel González # 95, ten human burials and skeletal remains from other species were recovered, along with ceramic objects associated with the Azteca II and Azteca III types (AD 900-1200, early post-Classic period). At a depth of 0.35m, and belonging to Layer II of the initial excavation unit, which corresponded to Cuadro A1NE, the first burial (Burial 1; MXV001 in this paper) was found in a fair state of conservation. This burial included an infant who was resting on their right side in a flexed position, oriented from west to east. The skull, although fractured, was facing south and the upper extremities were found semi-flexed towards the sides of the body, while the lower extremities were flexed towards the abdomen. From this site, this individual (MXV001) was the only one of the ten recovered individuals with visible osteological evidence of treponematosis. An offering was also recovered from this burial, consisting of three anthropomorphic figurines (a Warrior, a Musician and a Woman) as well as a small bowl located near the individual’s pelvis ^35,36^.

MXV001 is a genetically male non-adult with an estimated age of 2 years +/- 4 months based on dental development, a lack of fusion of the metopic suture and anterior fontanelle, and the incomplete fusion of the neural arches^37,38^. The skeleton was incomplete with missing os coxae, sternum, sacrum, and most of the hand and foot bones. The remaining bones demonstrated a variety of taphonomic effects, including delamination or exfoliation of bone (mostly on the skull), plant root etching, fracturing, and discolouration from soil and fungal growths. This individual had porotic hyperostosis and slight porosity throughout the skull, including the maxilla, mandible and orbital roofs. Significant porosity and active osteolytic reactions were observed on the palatine processes of both maxillary bones. Involvement of the palate is known to occur in both acquired and congenital treponemal infections^39^. The edges of the nasal aperture appear to be slightly rounded and the anterior nasal spine is not prominent, though it is unclear if this is related to pathological bone resorption. Bilateral round bony protrusions with multiple fenestrations showing growing dentition within them (perhaps dentigerous cysts) were observed on the buccal surface of the mandible below the areas of the alveolar bone that would hold the canines and first deciduous molars.

Slight porosity was also widespread in the infracranial skeleton. The right clavicle and scapula presented with superficial focal cavitations, and the latter also presented serpentine lytic lesions. The sternal ends of several ribs are also abnormally expanded. All of the long bones demonstrate healed or healing lamellar periostosis and both tibiae have a related sabre-shin malformation. Generally, pathological lesions are more severe in the bones on the right side of this individual. Porous lesions were observed below the coronoid process and periostosis was present at the distal end of the right ulna. A large abnormally expanded area with trabeculated bone and periostosis is present at the distal end of the right radius. The right femur was broken in two parts post-mortem and, from this fracture, concentric new bone formations can be observed in the medullary cavity. Additionally, the right first metatarsal bone and the right calcaneus have destructive lesions that may be perimortem or postmortem in nature.

The elements sampled for this study include a right humerus and a right tibia. Both bones have dark brown stains from continued contact with soil minerals, and small black spots on the bones likely indicate the growth of fungal colonies. Both bones have anterior bowing, and lamellar periostosis can be observed throughout the diaphyses of these long bones, with porous woven periosteal new bone also observed on the posterior surface of the humeral diaphysis. Samples were taken from areas of the humeral and tibial diaphyses with periostosis.

**2.3 Jucusbamba, Peru, JUC013**

Correspondence to: casey_kirkpatrick@eva.mpg.de

**CHA79, PAJ 235 (Site name: Putquerurco), Jucusbamba Valley, Peru**


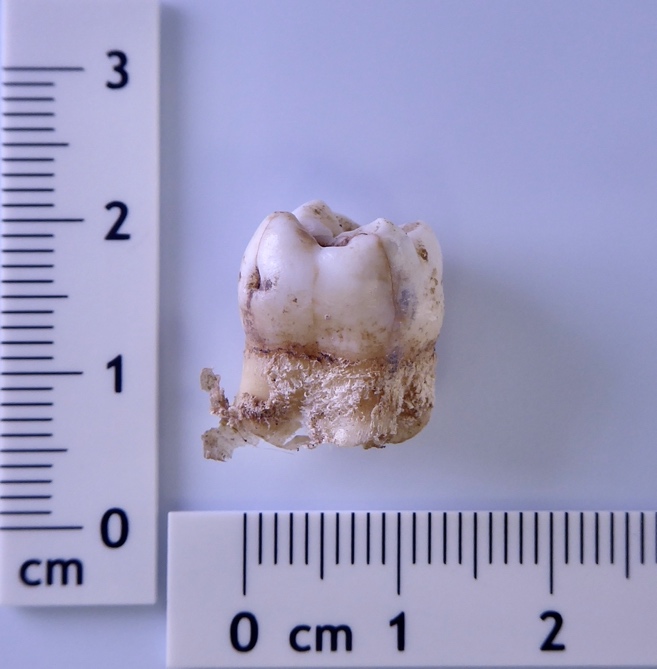


Figure S3 – Incompletely developed upper left first molar that was sampled from Individual JUC013.

JUC013 is a genetically male subadult with an estimated age of 6.5 (+/- 1) years based on dental development (Al Qahtani, 2009). This individual was discovered in a *chullpa* (funerary tower) associated with rock paintings in the site Putquerurco, Jucusbamba valley^40^. The valley is located on the eastern slopes of the north Peruvian Andes, a territory considered part of the Chachapoya realm. The sampled element is an incompletely developed upper left first molar with open roots through which the dentin was drilled. The tooth has a small amount of desiccated soft tissue still attached to the roots and it shows no evidence of pathology. This tooth was collected from a skull that was not available for study.

**2.4. Deán Funes, Argentina, DFU001**

Correspondence to: casey_kirkpatrick@eva.mpg.de and gabrieladape87@gmail.com

**1591/11, Deán Funes, Ischilín Department, Córdoba Province, Argentina**

 
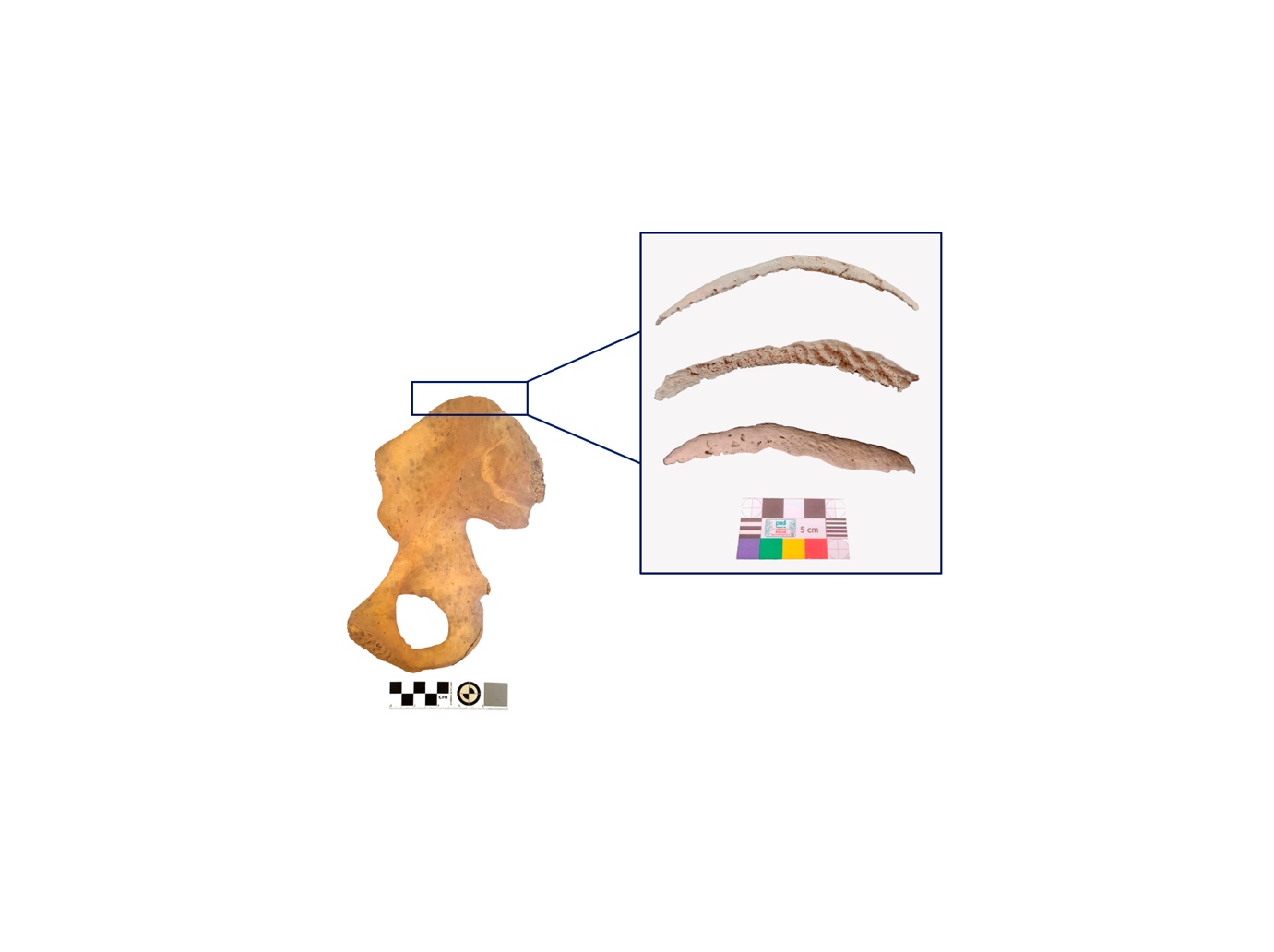


Figure S4. Left: The left ilium and the location at which the left iliac crest (also pictured from lateral and inferior view) rested prior to death. Right: Close-up views of the iliac crest (from top to bottom: lateral, inferior, and superior views).

The skeletonized remains of this individual were recovered near the town of Deán Funes, Ischilín Department, Córdoba province, Argentina, through a joint initiative by the Judicial Police and the National University of Córdoba’s Faculty of Philosophy and Humanities. Unfortunately, further contextual information is missing for this individual, who is genetically male and has an estimated age-at-death between 15 to 20 years ^41,42,43^. To the best of our knowledge, there were no other individuals recovered from this site.

This individual has a variety of paleopathological lesions throughout his skeleton, including enamel discoloration, which has been attributed to a possible case of dental fluorosis^44^. In the right mandibular ramus, there is extensive irregular periosteal new bone formation and microporosity in some of the surrounding bone. There were also three small lytic lesions in the left mastoid process without evidence of healing, which may be attributable to mastoiditis; however, they may be taphonomic in nature^45^. A mixture of osteolytic and osteoblastic reactions was also visible in the manubrium and in the left calcaneus. Periosteal new bone formation was observed in both zygomatic arches, both first ribs, both os coxae, vertebral segments T5-T12 and L2-L5, and the left tibia. The diaphyses of the left tibia and fibula, and both femora also demonstrated striated lamellar bone formation, sometimes with slightly darker coloration from the cortical surface. Additionally, an area of exposed trabecular bone surrounded by periosteal new bone growth (possible *cribra humeralis*) was observed on the medial surface of the right humeral neck, and concave areas of exposed trabecular bone in both femoral necks are consistent with Allen’s fossae^46^. On the superoposterior surface of the medial condyle of the right femur, there is also an area of exposed trabecular bone with evidence of remodelling on the marginal cortical bone, which represents a likely case of osteochondritis dissecans^47^. Finally, the epiphysis of the right iliac crest presents evidence of both osteolytic and osteoblastic processes. Given the age of this individual, the osteoblastic changes on the inferior surface may be, in part, associated with the beginning of the fusion of this element to the iliac wing. However, the irregular appearance of this surface and the disorganized appearance of the woven new bone may be attributable to pathological osteoblastic and osteolytic processes, if not also taphonomic processes. On the superior and lateral surfaces, the combination of antemortem osteolytic and osteoblastic reactions is also apparent as small osteolytic lesions that can be observed in various stages of healing, including penetrating lesions at the lateral edges of the iliac crest, and osteolytic lesions with remodelled edges and bony depressions on the superior surface that represent the complete healing of some osteolytic lesions. Sampling from this epiphysis of the iliac crest produced the treponemal aDNA presented in this paper.

**2.5** **STC Línea 6 Metro Refinería Azcapotzalco, Mexico City, Mexico, RAZ007**

Correspondence to: casey_kirkpatrick@eva.mpg.de  and lourdes_couoh@inah.gob.mx

**INAH SURPMZAH Folio Real: 1ARH00001738, Burial 189**

 
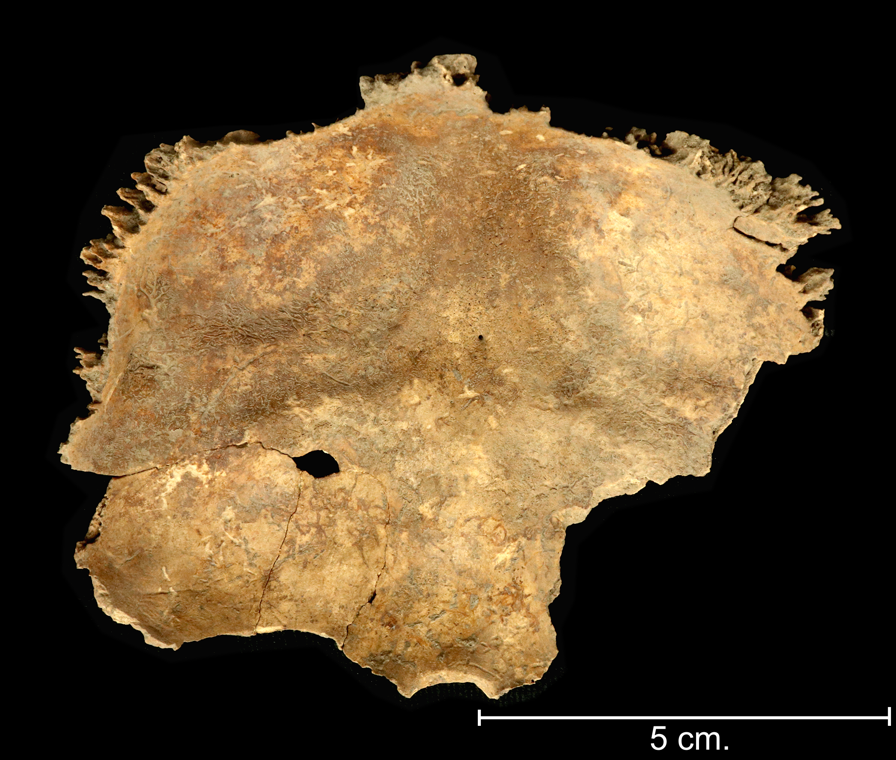
 
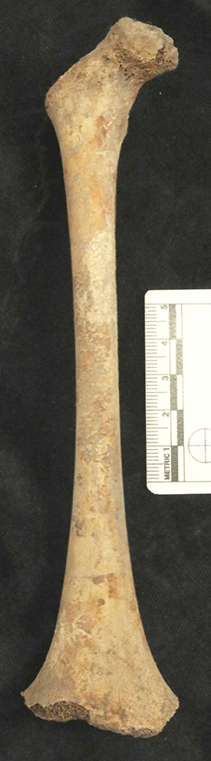


Figure S5: Skeletal elements sampled from Individual RAZ007. Left: fragmentary occipital bone with *serpens endocrania symmetrica*. Right: right femur with *cribra femoralis*.

The remains of Individual RAZ007 (Burial 189) were recovered in the early 1980s through a rescue excavation conducted by the *Instituto Nacional de Antropología e Historia* (INAH) in the neighborhood of Azcapotzalco, in the northwest of present-day Mexico City. This rescue project, called ‘*STC Línea 6 Metro Refinería Azcapotzalco’*, excavated and documented archaeological remains during the construction of the underground metro station named ‘Azcapotzalco’ by the *Sistema de Transporte Colectivo (*STC*)*. This excavation revealed the remains of a residential complex with 326 human burials found in domestic contexts, sometimes with burial offerings. Due to preservation issues, only 255 of these burials (284 individuals) could be recovered and bones and teeth from 35 of these individuals with osteological evidence of pathology were sampled for archaeogenetic analysis. The archaeological context suggests that all these burials belonged to the Tepanecan ethnic group^48^ that settled in the Basin of Mexico between 1220-1272 CE, and expanded to form the ‘Tepanecan Empire’ from 1375 to 1426 CE^49^. Following a war in 1428, the Mexicas assumed control of the Basin of Mexico; however, the Tepanecan culture continued to thrive in certain areas of Azcapotzalco under Mexica rulership^50^. Unfortunately, most of the documentation for this site has since been lost. However, the remaining archaeological data and radiocarbon dating have revealed that this archaeological site dates to the Late Post-Classic Period (ca. 1200-1520 CE) with usage likely stopping before the time of Spanish colonization (i.e. 1521 CE).

Radiocarbon dating (cal AD 1300 – 1397 2σ) clearly placed individual RAZ007 within the Tepanecan culture. This individual is genetically male and estimated to be 4.5 years old according to the stages of dental development and eruption^37^ and a femoral diaphyseal length of 190 mm^51^.

The remains of this individual are fragmentary and incomplete with evidence of taphonomic alterations in the form of bone fracturing, root etching, abrasion, discoloration, and patchy brown staining from sustained contact with soil. The skull is fractured post-mortem in multiple places with some parts missing, including the nasal bones and a Wormian bone from the right lambdoid suture. The clavicles, manubrium, right femur, and 16 vertebrae from this individual are unfused but mostly intact. The scapulae, humeri, radii, right ulna, right tibia, both fibulae, many of the ribs, and some of the metacarpals and hand phalanges are present but fragmentary. All of the infra-cranial bones were unfused and the epiphyses were missing.

Osteological evidence of pathology was visible throughout the recovered remains. The inferior edges of the nasal aperture were rounded, likely due to resorption, with porosity and remodeling visible in and around the nasal aperture. Unfortunately, the anterior nasal spine could not be observed due to post-mortem breakage. Porous lesions were also visible on the palatine process of the maxilla (the palatine bone was missing), the temporal bones, the *pars basilaris, and* both *pars lateralis*. Slight porosity and periosteal new bone were also observed in the area of the glabella, as well as bilateral symmetrical cribra orbitalia with level 2 severity and level 1 healing^52^. There was also endocranial porosity and thickening of the right parietal bone adjacent to the sagittal suture and *serpens endocrania symmetrica* (SES^53^) on the occipital bone, largely focused on and around the cruciform eminence. On the inferior surface of the sternal end of the left clavicle there is an ellipsoid depression with porosity and well-defined margins. Periosteal new bone was also observed on the anterior of both humeral diaphyses and on the posteroproximal region of the right tibial diaphysis, where there was also some porosity (the left tibia is missing). Additionally, the right femoral neck presented with porous lesions, i.e. *cribra femoralis*^54^ and the left femur is missing. Sampling from the distal end of the femoral diaphysis and the occipital bone produced the treponemal aDNA included in this study.

**3. Computational screening for *T. pallidum* in existing datasets**

Correspondence to: lesley_sitter@eva.mpg.de

#### **3.1 Sample database**

#### The Max Planck Institute for Evolutionary Anthropology hosts an extensive library of sequenced archaeological material from ~18000 individuals from different geographical locations and eras. A total of ~37000 sequencing datasets existed as of Feb 2022, which were dominated by geographic representation of Eurasia, Central and South America, and to a more limited extent North Africa. Of these, 19,770 datasets are raw shotgun data. Sequenced samples were automatically demultiplexed with bcl2fastq (https://support.illumina.com/sequencing/sequencing_software/bcl2fastq-conversion-software.html). For most datasets obtained from human dentition or bones, adapter clipping and merging of paired-end reads was automatically performed using leeHom^55^. Subsequently, the data were mapped to h19 genome using bwa v0.7.12^56^ (parameters: -n 0.01 -o 2 -l 16500) through an internal automatic pipeline. From these h19 mapped datasets, non-human reads were extracted from the produced BAM file using bam2fastq (https://gslweb.discoveryls.com/information/software/bam2fastq) v1.1.0 (parameters: --no-aligned --unaligned --no-filtered). Reads containing ambiguous nucleotides (Ns) or with length of <30bp were removed;

$ cat File_in.fastq | paste - - - - | awk -F "\t" '(length($2)>=30)&&($2!~/N/){print}' | tr '\t' '\n' | gzip -9 > File_out.fastq"

Resulting datasets were used for subsequent taxonomic screening.
The remaining sequenced datasets include those from specific pathogen and 1240K^57^ capture datasets, which also contain non-target sequences. For these capture datasets, raw sequencing reads were processed with TrimGalore^58^ [consisting of FastQC^59^ and CutAdapt^60^] for adapter removal and automatic removal of <30bp fragments and trimming of PHRED <30 base-calls at the 3’ and resulting datasets were directly used for taxonomic screening.


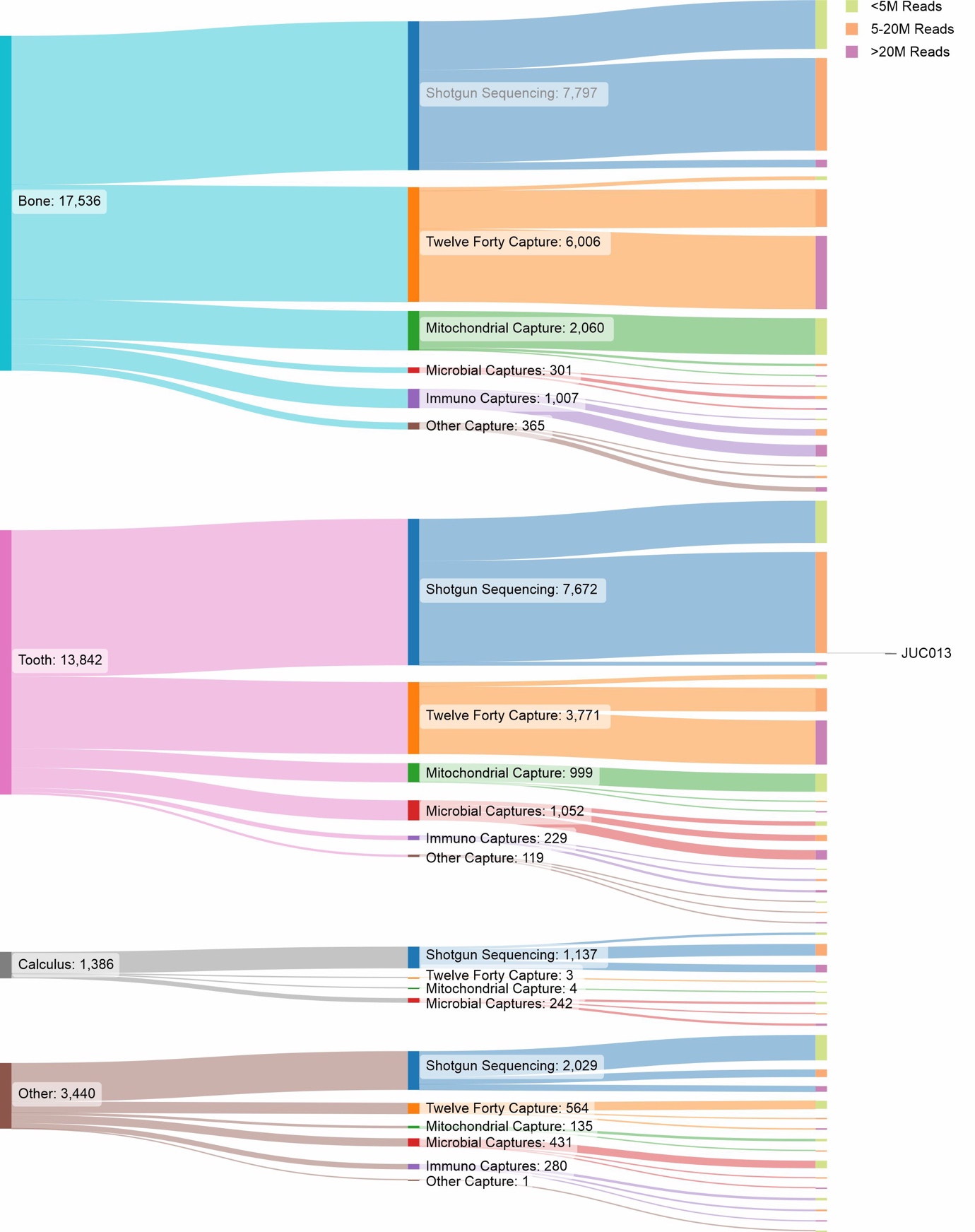


#### Figure S6 – Database composition for computational Treponema pallidum screening. Image generated using SankeyMatic (https://sankeymatic.com/)

#### **3.2 Performing taxonomic identification of reads using MALT**

Taxonomic identification of reads was performed using the MEGAN Alignment Tool (MALT) v0.5.2^61^ with a custom MALT database and index. The custom reference database was generated using all representative spirochete genomes (as of 24-02-2021), obtained from NCBI’s Assembly portal using the following search query;

("Bacteria"[Organism] OR "Archaea"[Organism]) AND (reference_genome[filter] OR representative_genome[filter]) AND (latest[filter] AND ("complete genome"[filter] OR "chromosome level"[filter] OR "scaffold level"[filter]) AND all[filter] NOT anomalous[filter]) AND ("Spirochaetia"[Organism] OR "Spirochaetes"[Organism])

This query resulted in the identification of 91 representative genomes. Inclusion of non-target genomes is necessary to facilitate a proper taxonomic assessment for the input data. Additionally, an Acc2Taxonomy file was generated by converting NCBI’s accession2taxid table (12-02-2021) (ftp://ftp.ncbi.nih.gov/pub/taxonomy/accession2taxid/nucl_gb.accession2taxid.gz), using the following command;

$ gunzip nucl_gb.accession2taxid.gz |cut -f1,3 | gzip -9 - > nucl-acc2tax.map.gz

Both the 91-reference genome multi fasta and the nucl-acc2tax.map.gz were supplied to MALT-build to generate an indexed MALT reference database. Subsequently, all processed ancient sequencing datasets were screened with MALT using malt-run, with a semiglobal alignment approach and a sequence identity cut-off of 90%.

$ malt-run \
-J-XX:ParallelGCThreads=1 \
-J-Xmx130G \
-d Big_screening_DB/malt_index/ \
-o ${SAMPLE}_rma6_output \
-id 90 \
-v false \
-t 16 \
-oa ${SAMPLE}_aligned_reads_output \
-m BlastN \
-at SemiGlobal \
-mq 25 \
-supp 0.01 \
-sup 0 \
-top 1 \
-mpi 0.0 \
-mrf 100000 \
--memoryMode load \
-i ${SAMPLE} \
1> ${SAMPLE}_malt-run_STDout.txt
2> ${SAMPLE}_malt-run_STDerr.txt


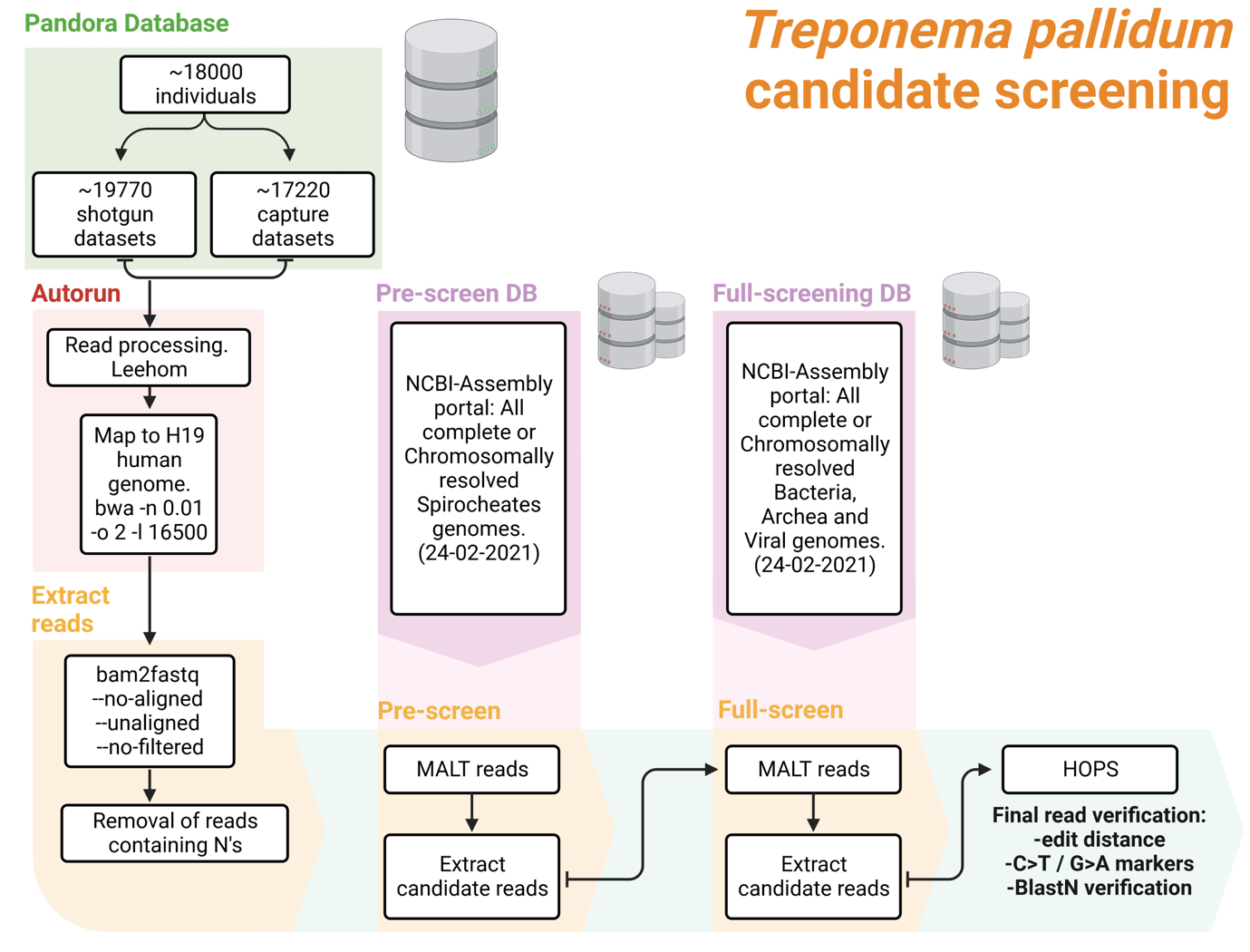


Figure S7: Protocol of computational dataset screening for *Treponema pallidum* DNA. Created in BioRender. Sitter, L. (2024) https://BioRender.com/r67y419

#### **3.3 Identifying Treponema pallidum subsp. pallidum candidate samples from MALT-run output**

The rma6 files generated by MALT were analysed for positive hits to our species of interest using the MaltExtract tool (https://github.com/rhuebler/MaltExtract)^62^. Manual assessment of MaltExtract’s summary files resulted in identification of >100 *Treponema pallidum* subsp. *pallidum* (TPA) positive datasets. The dataset was further limited by picking samples that exhibited characteristics of DNA damage often seen in ancient DNA such as 3’ G>A and 5’ C>T substitutions^63^. Additional removal of false positive hits was performed by running the TPA aligned reads from each candidate dataset against a larger Malt reference database containing 7508 bacterial genomes obtained from NCBI’s assembly portal (24-02-2021) using the following query;

("Bacteria"[Organism] OR "Archaea"[Organism]) AND (reference_genome[filter] OR representative_genome[filter]) AND latest[filter] AND (latest[filter] AND all[filter] NOT anomalous[filter])

Samples exhibiting reads matching to TPA in the second screening were further assessed using the negative difference proportion (NPD) as described in HOPS^62^.

**4.** **Radiocarbon Dating**

Radiocarbon dating was carried out at the Curt-Engelhorn-Centre for Archaeometry (Mannheim, Germany). Skeletal fragments were processed using ultra filtrated collagen (fraction > 30kD)^64,65^ and dated using the *MICADAS-AMS* of the Klaus-Tschira-Archäometrie Zentrum. Radiocarbon dates are reported showing the lab codes MAMS.

Radiocarbon and stable isotope measurements for evaluation of marine reservoir effects were made on the remains from Jucusbamba, Peru (JUC013), the Chonos Archipelago of southern Chile (GAP009), and Deán Funes, Argentina (DFU001) (see Methods). Send correspondence to W. Derek Hamilton, [derek.hamilton.2@glasgow.ac.uk](mailto:derek.hamilton.2@glasgow.ac.uk).

**4.1** **Radiocarbon measurement from the remains of individual MXV001**

Correspondence to: rodrigo_barquera@eva.mpg.de

This individual only had the δ^13^C measured on the AMS, which is not suitable for a dietary correction. However, this site is located over 600 km from the coast, so a marine reservoir effect is highly unlikely.

*Calibration*

Since this individual likely lived in an area around where they were recovered (Mexico City) the Northern Hemisphere, IntCal20, calibration curve of Reimer et al. (2020)^66^ was used to calibrate the radiocarbon results to a calendrical scale using OxCal v4.4 (Bronk Ramsey 2009)^67^.


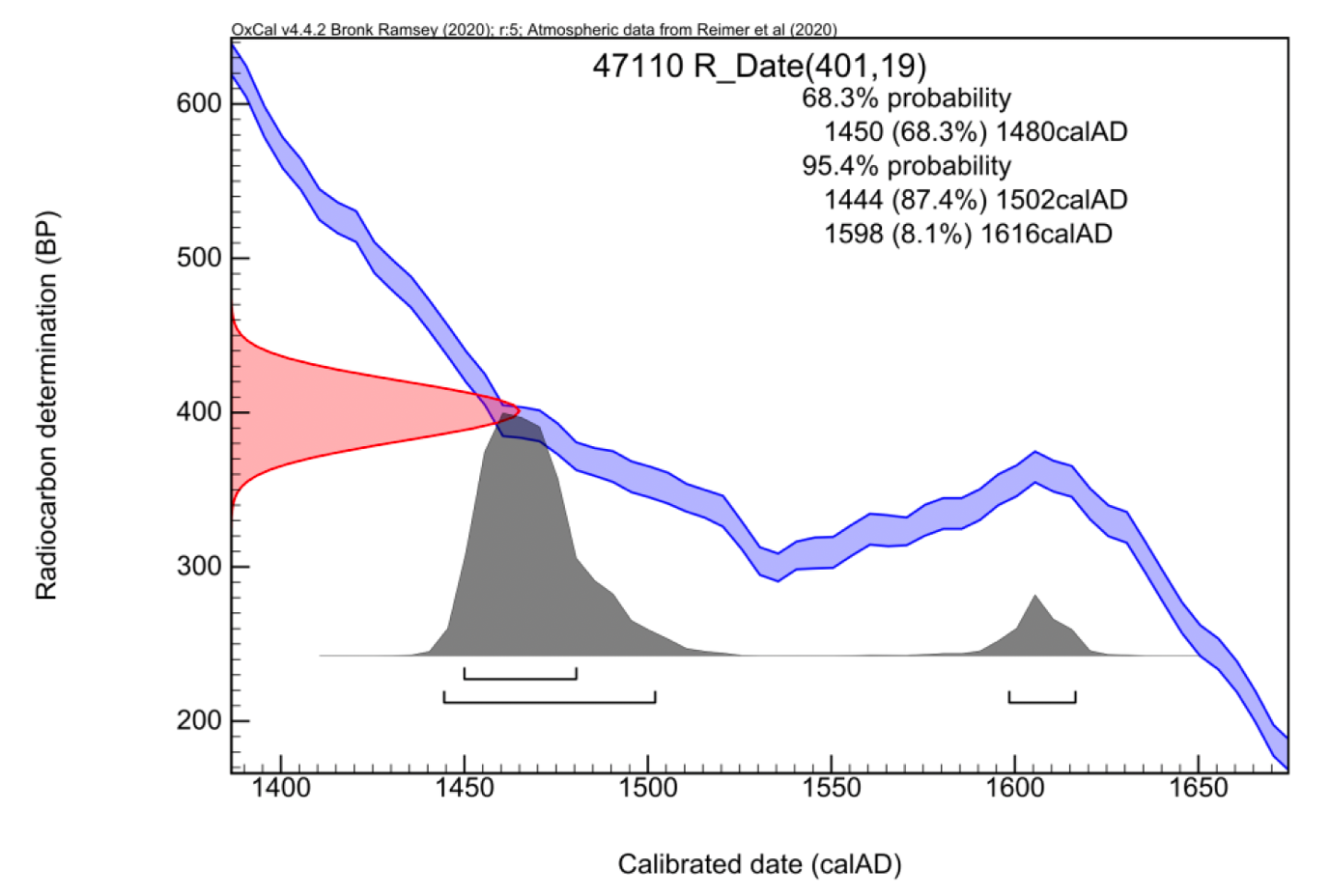


Figure S8 – AMS data for MXV001 with calibration in OxCal using IntCal20.

**4.2** **Radiocarbon measurements from the remains of individual RAZ007.B**

Correspondence to: lourdes_couoh@inah.gob.mx

This individual only had the δ^13^C measured on the AMS, which is not suitable for a dietary correction. However, this site is located over 600 km from the coast, so a marine reservoir effect is highly unlikely.

*Calibration*

Since this individual likely lived in an area around where they were recovered (Mexico City) the Northern Hemisphere, IntCal20, calibration curve of Reimer et al. (2020)^66^ was used to calibrate the radiocarbon results to a calendrical scale using OxCal v4.4 (Bronk Ramsey 2009)^67^.

The radiocarbon age calibrates to 1300–1397 cal CE (95% probability), indicating the individual died prior to known European contact in the region.


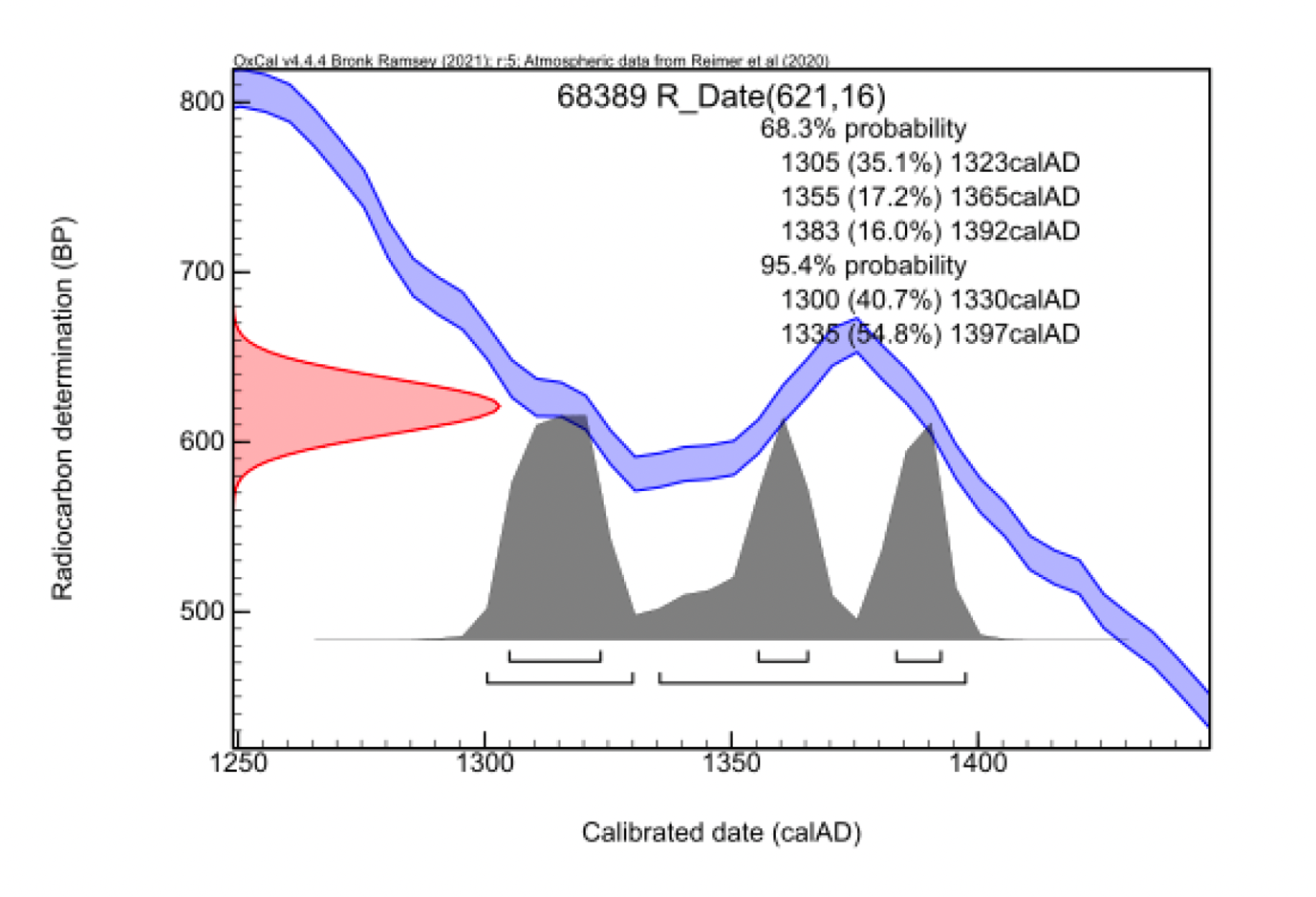


Figure S9: AMS data for RAZ007 with calibration in OxCal using IntCal20.

**5. Computational analysis of screening data from GAP009, MXV001, DFU001, and RAZ007**

Correspondence to: lesley_sitter@eva.mpg.de

Table S3: An overview of the different softwares and versions used by the nf-core/eager pipeline

| Software_Name | Version |
| --- | --- |
| nf-core/eager | v2.4.4 |
| Nextflow | v21.04.1 |
| FastQC | v0.11.9 |
| MultiQC | v1.12 |
| AdapterRemoval | v2.3.2 |
| BWA | v0.7.17-r1188 |
| Samtools | v1.12 |
| endorS.py | v0.4 |
| DeDup | v0.12.8 |
| Picard MarkDuplicates | v2.26.0 |
| Qualimap | v2.2.2-dev |
| Preseq | v3.1.1 |
| GATK UnifiedGenotyper | vPicked |
| DamageProfiler | v0.4.9 |
| bamUtil | v1.0.15 |


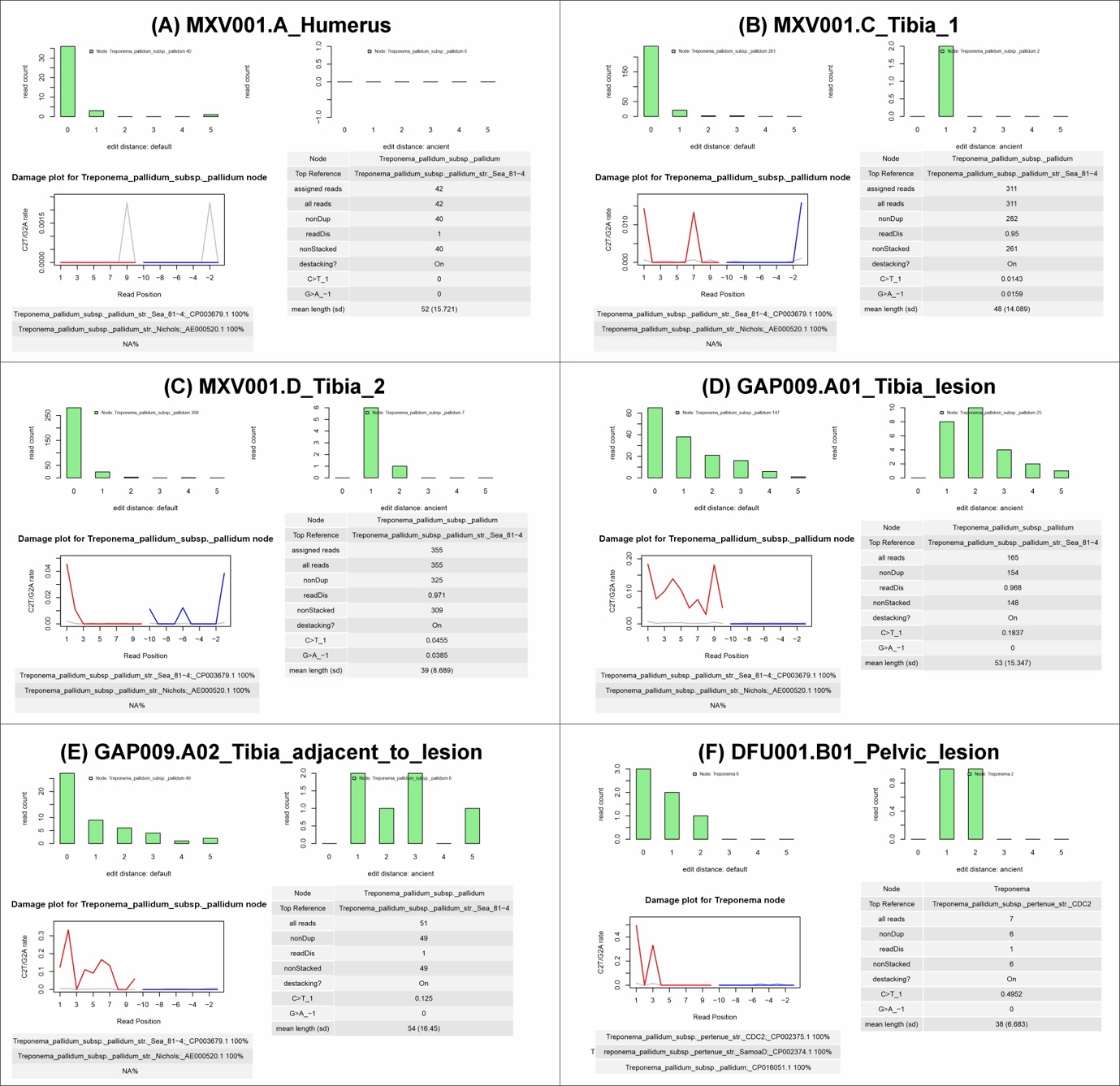


Figure S10 – HOPS^62^ results for *T. pallidum pallidum* identified reads for sequencing data from MXV001 (A-C) and GAP009 (D, E) and DFU001 (F).

**6.** **Analyses of *Treponema pallidum subsp. pallidum* captured data**

Correspondence to: [lesley_sitter@eva.mpg.de](mailto:lesley_sitter@eva.mpg.de)

#### **6.1 Capture validation through reference mapping**

Sequencing data generated from *Treponema pallidum* subsp. *pallidum* (TPA) captured samples were analysed in nf-core/eager. TPA Nichols (RefSeq ID NC_021490.2)^68^ was used as a reference genome. Datasets were supplied as a tsv file using option –input. EAGER2 was run with the following command:

$ nextflow run nf-core/eager \
-r 2.4.4 \
-profile conda \
–name ‘Tpallidum’ \
–input ‘Sample_table.tsv’ \
--fasta NC_021490.2.fasta' \
--outdir './Outdir' \
--clip_adapters_list sequencing_adapter_combinations.txt \
--run_post_ar_trimming \
--post_ar_trim_front 1 \
--post_ar_trim_tail 1 \
--bam_filter_minreadlength 30 \
--preserve5p \
--clip_min_read_quality 30 \
--run_bam_filtering \
--bwaalnn 0.1 \
--bwaalnl 32 \
--run_trim_bam \
--bam_mapping_quality_threshold 37 \
--run_genotyping \
--genotyping_tool 'ug' \
--genotyping_source 'raw' \
--gatk_ug_out_mode EMIT_ALL_SITES

For low-stringency mapping used for SNPevaluation^69^ analysis (see below), the –bwaalnn parameter was changed to 0.01, and the –bwaalnl was changed to 16.

Samples having a median coverage of <4x were analysed using the ClusterFactor ($CF=\frac{1}{1-Duplication rate})$, where a CF of >2 means individual reads have been sequenced on average two times. Only one sample, JUC013, yielded a positive signal but exhibited low coverage and a high CF, which indicated a potential benefit for the manufacture, enrichment, and sequencing of additional libraries.

#### 6.2 Competitive mapping

A competitive mapping to both *Treponema pallidum* subsp. *pallidum* str. Nichols (RefSeq ID NC_021490.2) and a relative outside of the human pathogenic complex, *Treponema paraluiscuniculi Cuniculi A* (RefSeq ID NC_015714.1), was performed to measure capture specificity. Both reference genomes were concatenated into a new multi-FASTA file and mapped as described in the previous section. The comparison was performed by analysing the median coverage over each genome using the following command:

$ samtools index mapped_reads.bam > mapped_reads.sorted.bam

samtools coverage --min-read-len 30 --min-MQ 37 mapped_reads.sorted.bam

Samples that did not pass the previously mentioned quality thresholds, or that mapped with higher values to *Treponema paraluiscuniculi Cuniculi A*, were excluded from downstream analysis.

#### 6.3 SNP Calling

Alleles were called using GATKs unified genotyper (UG) v3.8^70^, running within nf-core/eager. The *.vcf* files produced by UG where then processed using MultiVCFAnalyzer v0.85.2^71^. This software package uses a reference genome and several *.vcf* files, as generated by UG, as input and creates a multiple sequence alignment (MSA) of all the SNPs in the samples that pass a user defined depth call (snpalignment.fasta) as well as an MSA of the entire genome mapping (fullAlignment.fasta). It also generates a SNP table containing only alternative genotypes within the sample set. MultiVCFAnalyzer was run as follows:

java -Xmx10G -jar MultiVCFAnalyzer-0.85.2.jar \
NA \
NC_021490.2_Treponema_pallidum_subsp._pallidum_str._Nichols.fasta \
NA \
output_folder \
T \
37 \
[DEPTH_CALL] \
0.9 \
0.9 \
NA \
Sample1.vcf \
Sample2.vcf \
:
SampleN.vcf

[DEPTH CALL] was replaced with either ‘2’ or ‘4’ depending on the analysis that was performed.

**6.4 Analyses of genome KM14-7**

Here we investigate the genome KM14-7 (also described as “Kampen” and “Leiden”) from Majander et al. 2020^72^. This genome is radiocarbon dated to 1494-1631 CE, and has a published phylogenetic position within the common lineage of TPE and TEN, branching basal to their separation. As such it is presented by the authors as a possible representative of pre-Columbian treponemal diversity within Europe. We have investigated this genome in some detail. Mapping the datasets from Majander et al. 2020^72^ with the stringency parameters described in this study yields the following coverage results:

Table S11: Mapping differences between the method employed in this publication and the statistics reported in Majander et al. 2020^72^.

| **Library** | **Nr. Dedup. Mapped Reads** | **Avg / Mean Cov** | **≥ 1X** | **≥ 2X** | **≥ 3X** | **≥ 5X** | **Median read length** | **Source** |
| --- | --- | --- | --- | --- | --- | --- | --- | --- |
| PD28 | 1430292 | 136.23X | 98.09% | 98.07% | 98.06% | 98.04% | 59,91 | Majander et al. 2020 |
|  | 1484214 | 78.3X | 98.10% | 98.10% | 98.00% | 98.00% | 57,00 | Stringent mapping |
| SJ219 | 29198 | 1.41X | 64.31% | 34.00% | 15.69% | 2.58% | 49,12 | Majander et al. 2020 |
|  | 32292 | 1.4X | 64.30% | 29.00% | 11.20% | 8.90% | 44.22 | Stringent mapping |
| CHS119 | 52054 | 2.82X | 83.32% | 62.31% | 42.04% | 15.13% | 54,16 | Majander et al. 2020 |
|  | 60234 | 2.9X | 83.40% | 56.30% | 32.40% | 32.30% | 49.87 | Stringent mapping |
| KM14-7 | 18034 | 0.91X | 46.89% | 19.35% | 7.93% | 1.61% | 51,69 | Majander et al. 2020 |
|  | 13898 | 0.6X | 38.90% | 10.40% | 2.30% | 0.40% | 46.17 | Stringent mapping |

The strict parameters applied in our analyses reduced the coverage of 2-fold supported positions (the lowest possible coverage of SNP calling) in KM14-7 from 19.35% to 10.4%. Inspection of the SNP table in Majander et al 2020^72^ revealed positions in KM14-7 that matched allele calls found uniquely in TPA (n=10 shared with PD28) and others that are unique to TPE (n=10 shared with CHS119) (Figure S11).


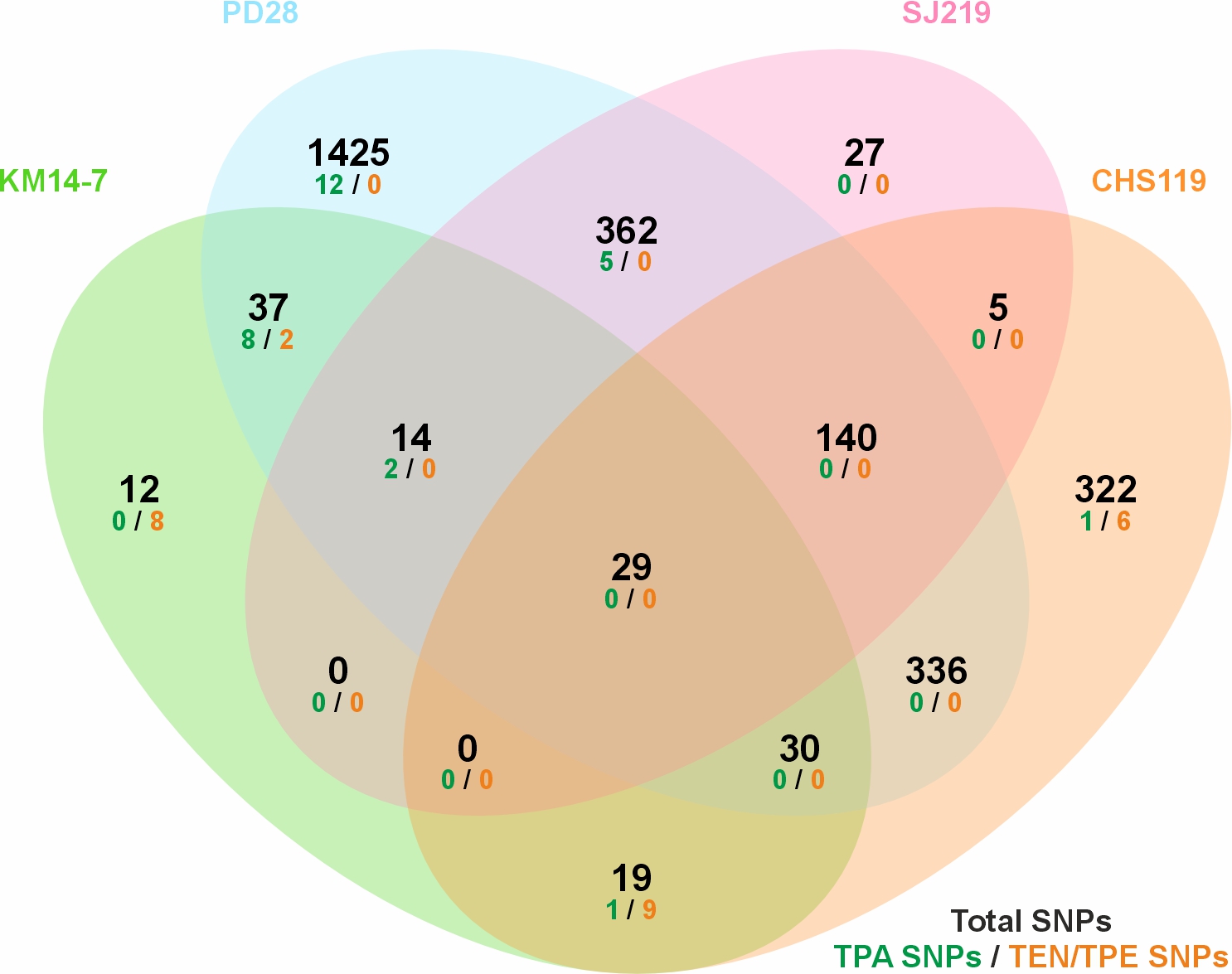


Figure S11: Visual representation of SNPs reported in Majander et al. 2020^72^ for their ancient genome datasets. TPA = *Treponema pallidum pallidum*, TEN = *Treponema pallidum endemicum*, TPE = *Treponema pallidum pertenue*.

To further investigate the phenomenon of shared SNPs between datasets of genomes that represent different *T. pallidum* sublineages, we extracted reads that mapped to the Nichols *T. pallidum* reference from all Majander et al. 2020^72^ datasets (based on our strict mapping) and queried them for reads that are identical (same nucleotide sequence) within the four datasets tethered to the publication (PD28, KM14-7, SJ219, and CHS119 (Table S12).

Table S12: Reads with identical nucleotide sequence contained within the datasets of mapping reads from libraries KM14-7, PD28, and CHS119.

| **Sample** | **Mapped Reads** | **Identical in PD28** | | **Identical in SJ219** | | **Identical in CHS119** | | **Total shared reads** |
| --- | --- | --- | --- | --- | --- | --- | --- | --- |
|  |  | **Nr.** | **%** | **Nr.** | **%** | **Nr.** | **%** |  |
| KM14-7 | 12784 | 1397 | 10.9% | 275 | 2.2% | 241 | 1.9% | 15.0% |
| CHS119 | 27675 | 1418 | 5.1% | 799 | 2.9% |  |  | 8.0% |
| SJ219 | 44493 | 1220 | 2.7% |  |  |  |  | 2.7% |

Of the three genomes, KM14-7 shows the greatest influence of read sharing with other datasets. Such a phenomenon could come from sequencing “cross talk”, which is documented to occur with Illumina sequencing^73^, though known examples are smaller in scale. To investigate if identical reads could exist by chance between ancient datasets, we compared the KM14-7 raw read datasets against those from the other three samples published alongside it (PD28, SJ219, and CSH119), as well as three additional ancient treponemal datasets published two years earlier (2018), namely 133, 94A, and 94B^74^. This was accomplished by reducing the fastq files to only the nucleotide sequences (sed -i ‘1,2-3~4d’ sample.fastq) , sorting them (sort sample.fastq > sample.fastq.sort), removing duplicate entries (uniq sample.fastq.sort > sample.fastq.sort.uniq), and comparing samples in a pairwise method (comm -12 sample.fastq.sort.uniq sample2.fastq | wc -l). Through this process no duplicate reads were identified between KM14-7 and datasets from the 2018 publication^74^ (Table S13).

Table S13. Evaluation of identical DNA reads contained within several published raw datasets from human archaeological bone yielding *T. pallidum* genomes. KM14-7, PD28, CHS219, and SJ219 from Majander et al. 2020^72^. 133, 94A and 94B from Schuenemann et al, 2018^74^. “combined” refers to joined individual data files from paired end sequencing.


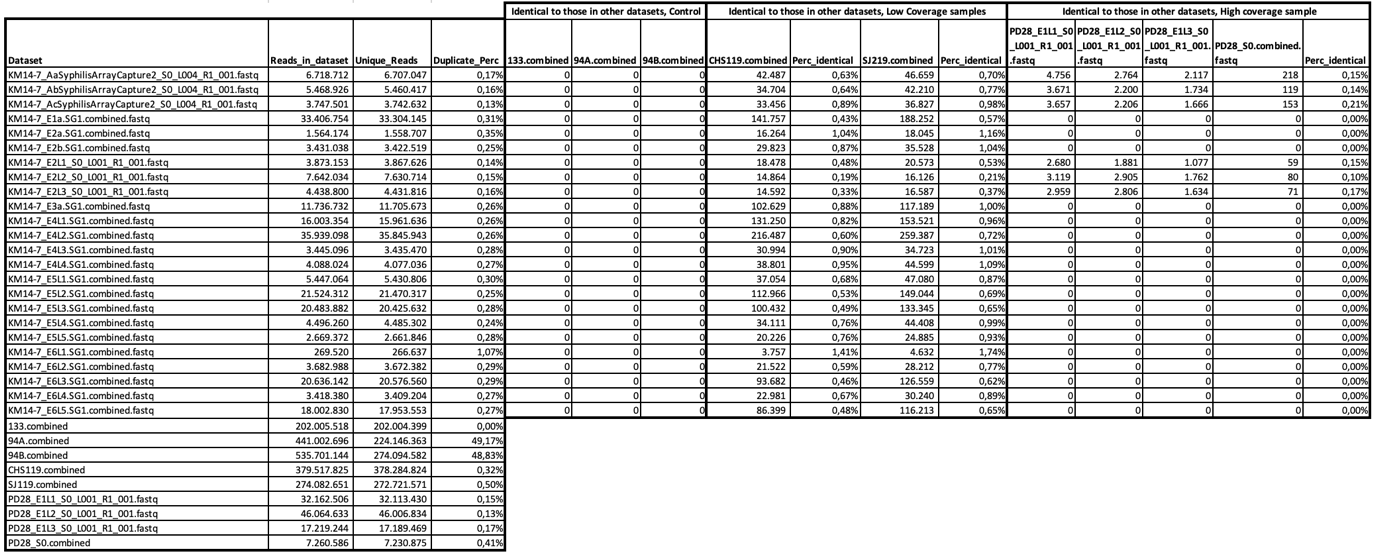


Whatever the reason for read duplication between the Majander et al. 2020^72^ datasets, we investigated genomic coverage and allele calls with duplicate reads across datasets removed. Despite the read duplication phenomenon having impacted all four genomic datasets in Majander et al 2020^72^, mapping post removal of these duplicate reads was investigated exclusively in KM14-7 because of its very low coverage (Tables S14 and S15).

Table S14: Strict mapping of dataset KM14-7 after removal of reads that are identical to those contained in datasets for PD28, CHS119, and SJ219.

| Sample Name |  | Nr. Dedup. Mapped Reads | Avg / Mean cov | ≥ 1X | ≥ 2X | ≥ 3X | ≥ 5X | Median read length |
| --- | --- | --- | --- | --- | --- | --- | --- | --- |
| KM14-7 | Before Filtering | 13,898 | 0.6X | 38.90% | 10.40% | 2.30% | 0.40% | 46.2 |
| KM14-7 | After Filtering | 11,018 | 0.5X | 34.00% | 8.30% | 1.60% | 0.10% | 48.0 |

The remaining data are too low for phylogeny construction based on 2-fold read support, the lowest coverage needed for SNP calling in GATK. For transparency, we report all 2-fold support allele calls for KM14-7 in Table S15. Here, no alleles show a genotype that would place KM14-7 within the TPA clade. This indicates that all KM14-7 positions previously identified by Majander et al^72^ as having the TPA allele have been removed either via our strict mapping or our removal of identical reads shared with other datasets. One position (643984) is shared exclusively with the TEN genome Bosnia A, though this position is removed via quality filters applied prior to tree construction (see section 8). For an additional position (474902), KM14-7 shares an allele common to the *T. paraluiscuniculi* outgroup and TPE/TEN. The position is, therefore, derived in TPA only. The remainder of the positions are unique to KM14-7 or are reference calls in all genomes considered. Based on this, KM14-7 has too few positions to permit its sublineage designation, and consequently it is not considered in further analyses.

**7. SNP filtering**

Correspondence to: lesley_sitter@eva.mpg.de

SNPs generated on low-complexity regions, regions susceptible to non-target mapping or recombination sensitive regions were removed to prevent spurious SNPs from interfering with phylogenetic analyses of vertically evolved genomic regions.

**7.1 Low-complexity regions**

Low complexity regions were identified with dustmasker v1.0.0^75^ with the following command;

$ dustmasker -in NC_021490.2_Treponema_pallidum_subsp._pallidum_str._Nichols.fasta -out NC_021490.2_Treponema_pallidum_subsp._pallidum_str._Nichols_repeatlist.txt -outfmt acclist

Dustmasker output was converted into a GFF3 formatted file as such:

$ sed -i '1s/^/\#\#sequence-region NC_021490.2 1 1139633\n\#\#species https\:\/\/www.ncbi.nlm.nih.gov\/Taxonomy\/Browser\/wwwtax\.cgi\?id=243276/g' NC_021490.2_Treponema_pallidum_subsp._pallidum_str._Nichols_repeatlist.gff3

$ sed -i "s|>NC_021490.2 Treponema pallidum subsp. pallidum str. Nichols, complete sequence    |NC_021490.2    DustMasker    region    |g" NC_021490.2_Treponema_pallidum_subsp._pallidum_str._Nichols_repeatlist.gff3

$ sed -i 's/\n|    .    +    .    ID="Repeat"\n/g' NC_021490.2_Treponema_pallidum_subsp._pallidum_str._Nichols_repeatlist.gff3

Subsequently, repeats that were <30bp were removed from the GFF3 for these should be mappable with >30bp reads.

**7.2 Highly conserved genomic regions**

Due to the metagenomic nature of ancient pathogen captures, combined with the allowance of mismatches to accommodate for actual SNPs and DNA damage, tRNA and rRNA regions are prone to background noise due to their high genomic conservation. These regions are, therefore, masked out by extracting their locations from the *.gff3* file that is associated with the RefSeq deposited *Treponema pallidum* subsp. *pallidum* str. Nichols (RefSeq ID NC_021490.2) reference genome.

$ grep "[r,t]RNA" NC_021490.2_Treponema_pallidum_subsp._pallidum_str._Nichols.gff3 > NC_021490.2_Treponema_pallidum_subsp._pallidum_str._Nichols.tRNA.gff3

$ grep "[1-2][0-9]s" NC_021490.2_Treponema_pallidum_subsp._pallidum_str._Nichols.gff3 >> NC_021490.2_Treponema_pallidum_subsp._pallidum_str._Nichols.tRNA.gff3

**7.3 Recombinant positions**

Recombinant sites were identified through the union of both Gubbins v3.2.1^76^ and ClonalFrameML^77^ output. For increased sensitivity of recombination detection we used the union of identified regions from both tools.

To run Gubbins, the following command was run on the fullAlignment.fasta generated for MultiVCFAnalyzer execution respectively:

$ run_gubbins.py --threads 4 --verbose --min_window_size 3 fullAlignment.fasta

Gubbins outputs a GFF3 formatted file (fullAlignment.recombination_predictions.gff) containing all the identified recombinant sites. To run ClonalFrameML, a “starter tree” is needed which was generated using FastTree v2.1.11^78^ using the following command:

$ FastTree -gtr -nt < fullAlignment.fasta > Fastree.tree

To run ClonalFrameML, the following command was used:

$ ClonalFrameML Fastree.tree fullAlignment.fasta ClonalframeML_output

ClonalFrameML does not produce a GFF3 format file containing the identified recombinant positions. Instead it generates an ‘. importation_status.txt’ file that was converted into a GFF3 format type file using a custom script.

**7.4 Regions prone to non-target mapping**

To investigate the persistence of DNA from non-target sources in our captured datasets, we investigated the density of multi-allelic positions considered in phylogenetic construction. This process revealed a dominance of homozygous positions, consistent with a low non-target background.


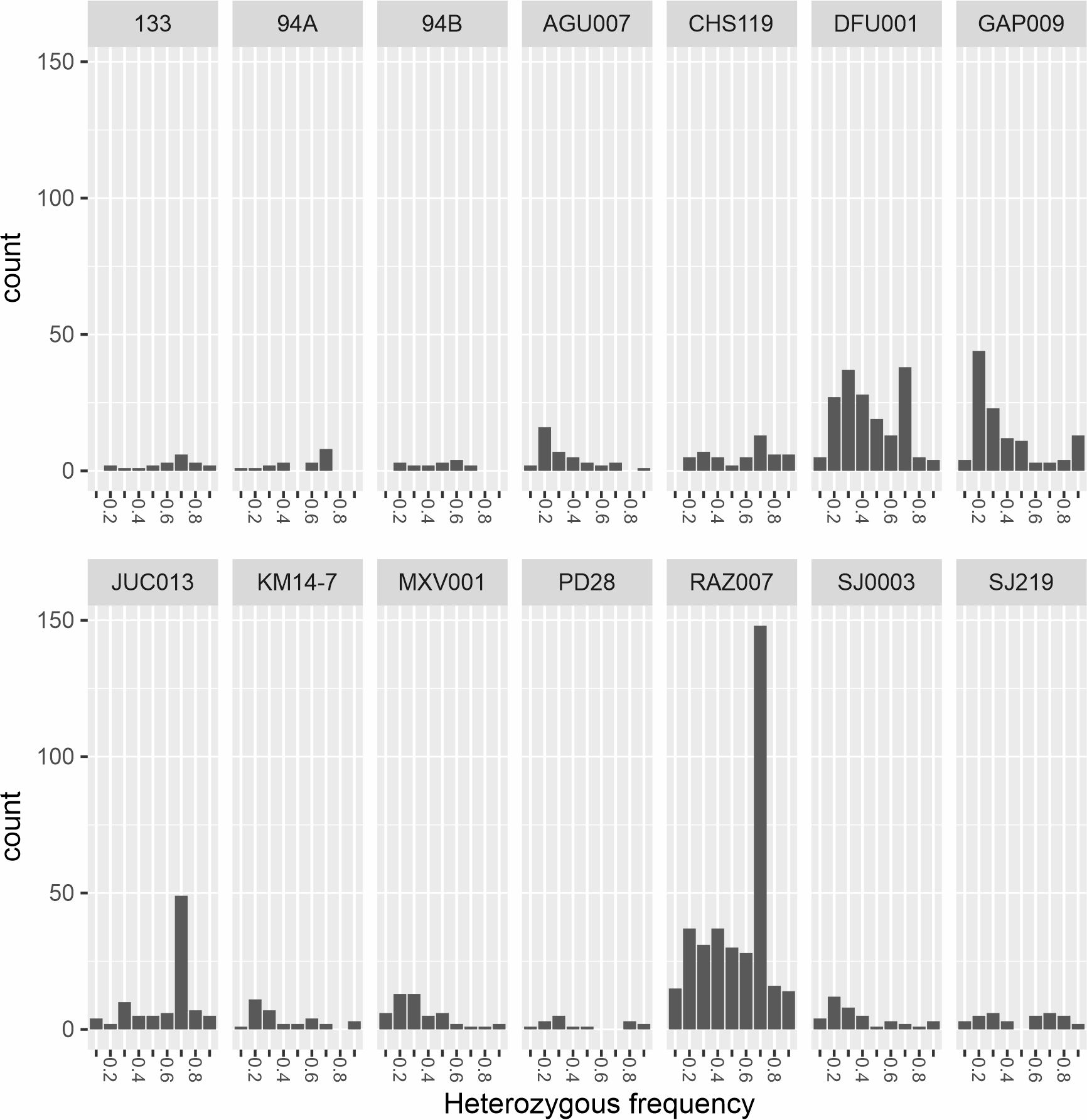


Figure S12: Heterozygous positions based on 0.1 to 0.9 frequency across mapped regions.

We further explored this potential artifact through use of SNPEvaluation^79,69^ to identify regions of suspicious homology and mapping artifacts. For ancient genome samples an additional nf-core/eager run was performed as previously described, with –bwaalnn 0.01, and the –bwaalnl 16 parameters. The SNPtable.tsv from the MultiVCFAnalyzer output as well as the stringent and low stringent *.vcf* files were supplied. We identified all positions that 1) resulted in a summary statistic >1.1 (i.e., more reads mapped when stringency is lowered), 2) were determined to be heterozygous themselves, or 3) to have additional sites within a frame of 50bp with no coverage or with heterozygous calls. Alleles that failed any of these criteria were extracted and their coordinates converted into GGF3 format for subsequent masking.


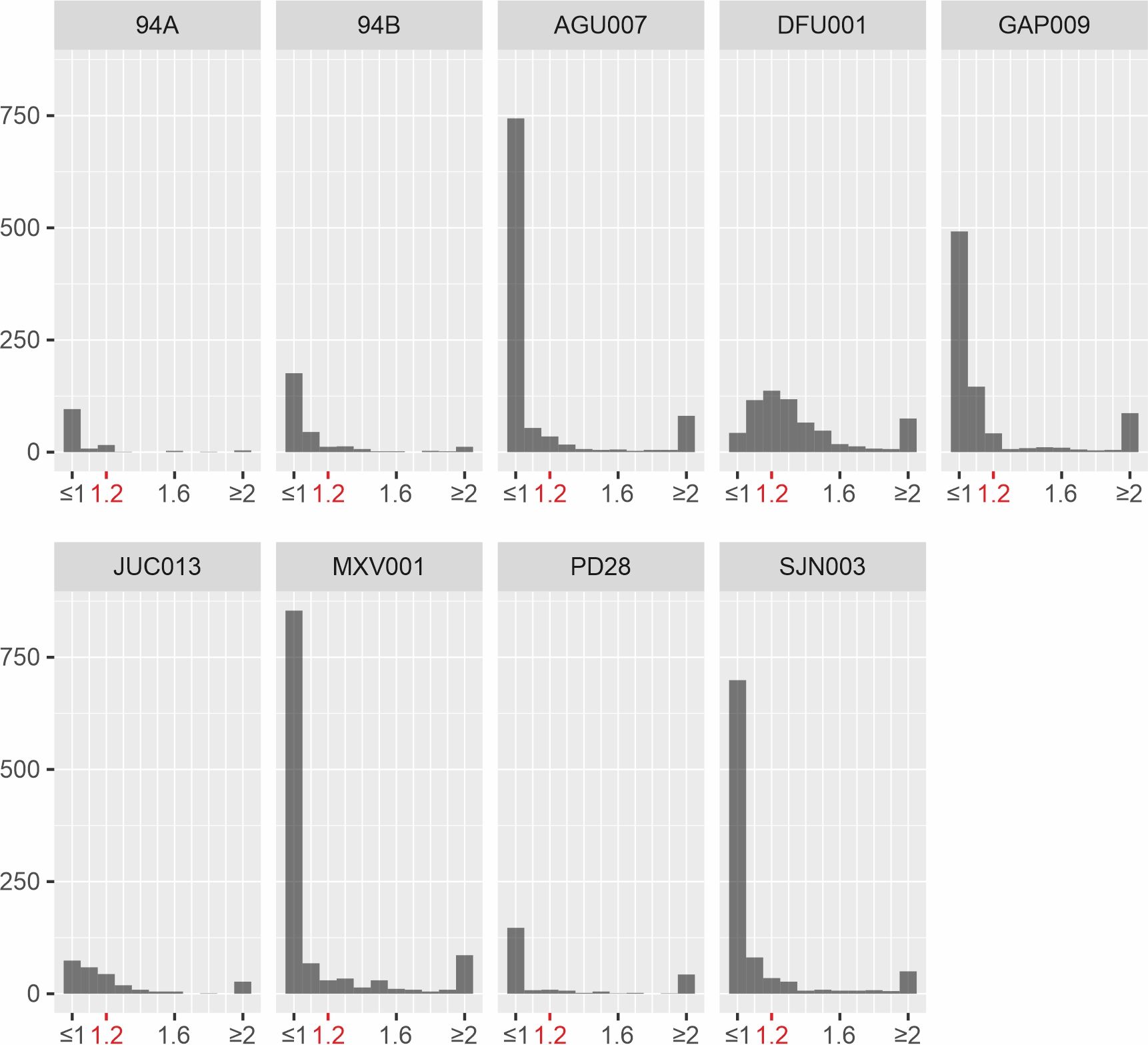


Figure S13: Distribution of the SNP Evaluation summary statistic. Values above 1 represent sites that tend to have increased depth at lower stringency mapping or have another artefact that suggest it’s prone to background noise. Normally a cutoff of around 1.1 is used to make sure that the analysed SNPs are not artefactual.

**7.5 Masking of spurious regions from genotyping**

The previously produced five *.gff3* files containing sites that should be excluded from SNP analysis were concatenated to generate a single gff3 formatted masking file. To mask these spurious regions from genotyping, MultiVCFAnalyzer was once again run but now supplied with the masking *gff3* file as excluded regions.

java -Xmx10G -jar MultiVCFAnalyzer-0.85.2.jar \
NA \
NC_021490.2_Treponema_pallidum_subsp._pallidum_str._Nichols.fasta \
NA \
output_folder \
T \
37 \
[DEPTH_CALL] \
0.9 \
0.9 \
Mask_from_genotyping.gff3 \
Sample1.vcf \
Sample2.vcf \
:
SampleN.vcf

This second MultiVCFAnalyzer run generates a SNPtable.tsv and both a SNPalignment.fasta and a fullalignment.fasta MSA file which are used in further downstream phylogenetic analysis.


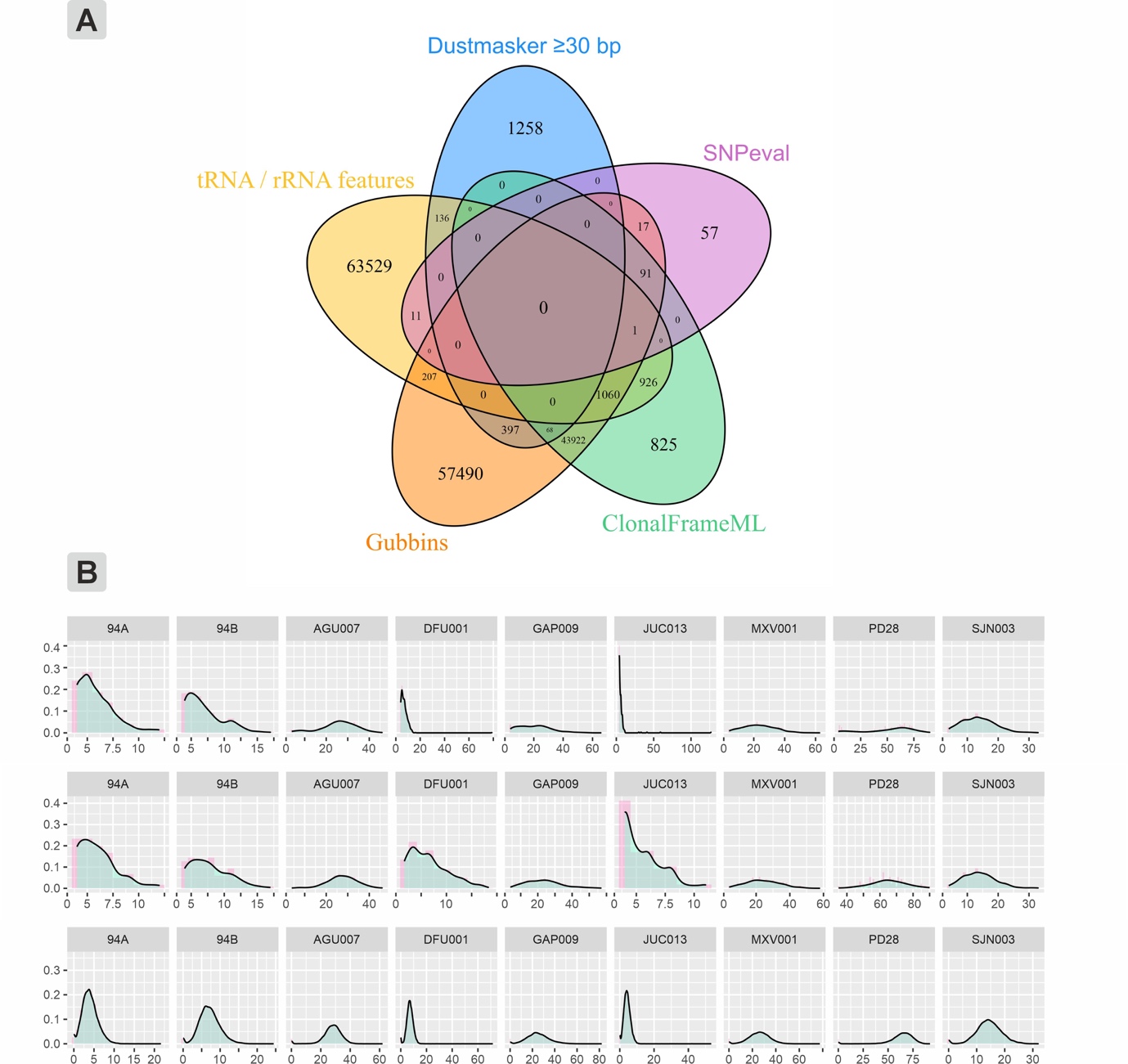


Figure S14: SNP filtering was performed using different metrics and criteria. A) A breakdown of each approach and the number of loci masked from SNP calling. Dustmasker filters low-complexity sites, ClonalFrameML and Gubbins identify recombinant regions within the analysed genomes, tRNA and rRNA features were taken directly from the gene features associated with the reference genomes as they are deposited on GenBank, lastly SNPeval identifies low confidence loci. B) Additionally, we investigated whether or not we had particular sites that accumulated substantially higher coverage than the global average, which could indicate sites prone to background noise.

**8.** **Phylogenetic assessment of *T. pallidum* genomes**

Correspondence to: lesley_sitter@eva.mpg.de

Maximum Likelihood (ML) phylogenetic tree construction was performed using RAxML-NG v1.10^80^ as described by Giffin et al.^81^ and Majander et al^72^. Trees were generated with 1000 bootstrap replicates, using a GTR model and GAMMA substitution correction model with 8 categories. Additionally, Stamatakis ascertainment bias correction was added by calculating the total number of constant sites for each nucleotide remaining after all filtering steps were applied (note that alignment positions containing missing data but for which all resolved sites were identical were also considered constant).

$ raxml-ng --all --msa Filtered_Fastas/FouX_RemovedInvariate.Fasta --msa-format FASTA --data-type DNA --prefix FourX_All-Sites_RaxmlNG_JC_G8_ASC_STAM --seed 12345 --threads 4 --model GTR+G8+ASC_STAM{226995/244265/250641/228906} --bs-trees 1000

Using FigTree v1.4.4 (http://tree.bio.ed.ac.uk/software/figtree), trees were rerooted using *Treponema paraluiscuniculi* as the outgroup, with outgroup-specific SNPs hidden to prevent extremely long branch length and skewing of the tree. Pruning of the outgroup’s unique SNPs was achieved through MultiVCFAnalyzer by picking an outgroup organism and prefixing its vcf with ‘outgroup_’.


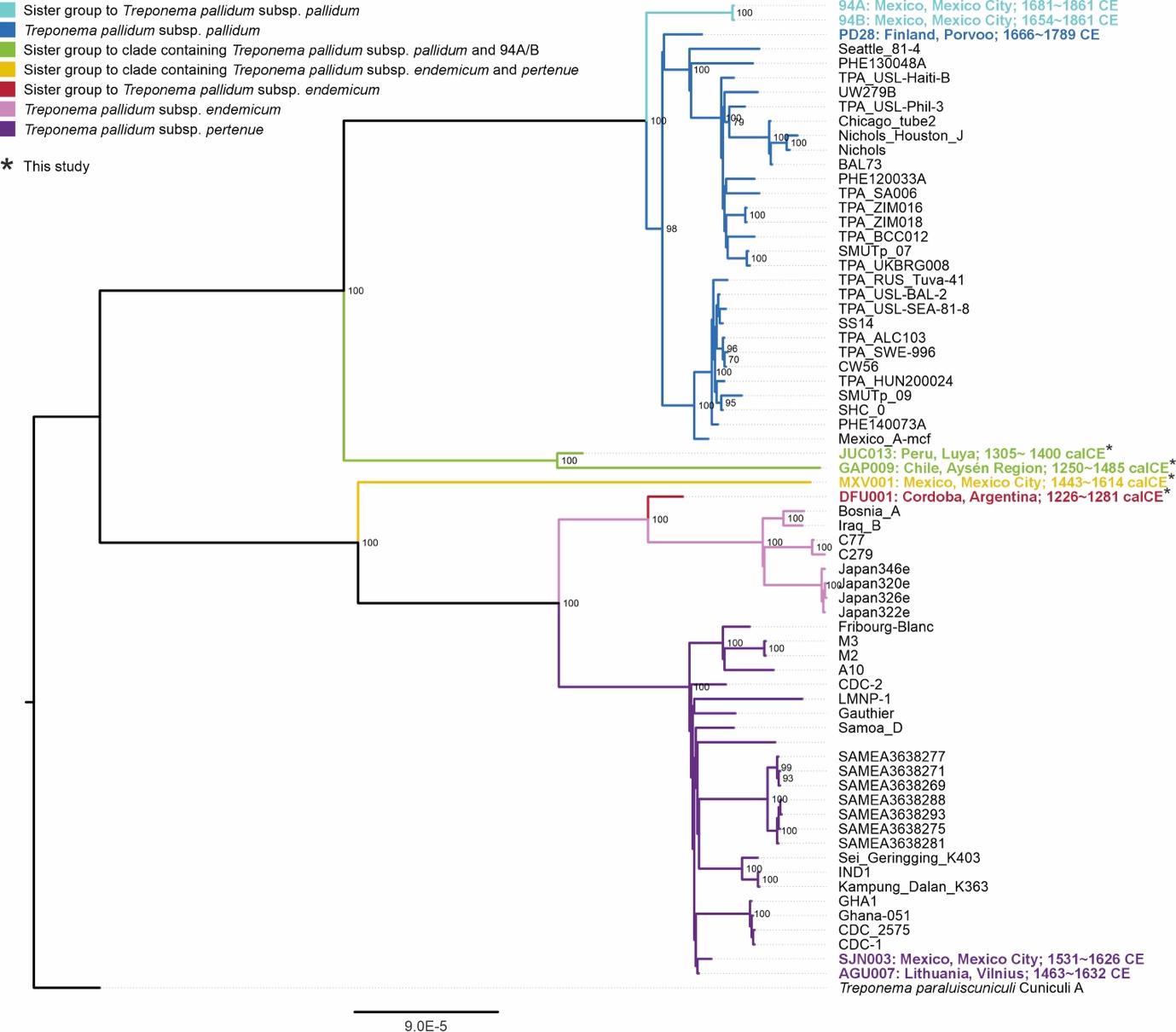


Figure S15: Maximum likelihood tree constructed without removal of positions identified by SNPEvaluation as being potentially from non-target sources (to demonstrate long branch lengths in GAP009 and MXV001). The tree was generated using the 2160 SNPs called at 4-fold coverage without recombination regions removed. The tree is based on 1000 bootstrap replicates, where branches with <70% support are not shown. Names of ancient genomes are coloured according to their corresponding clade.


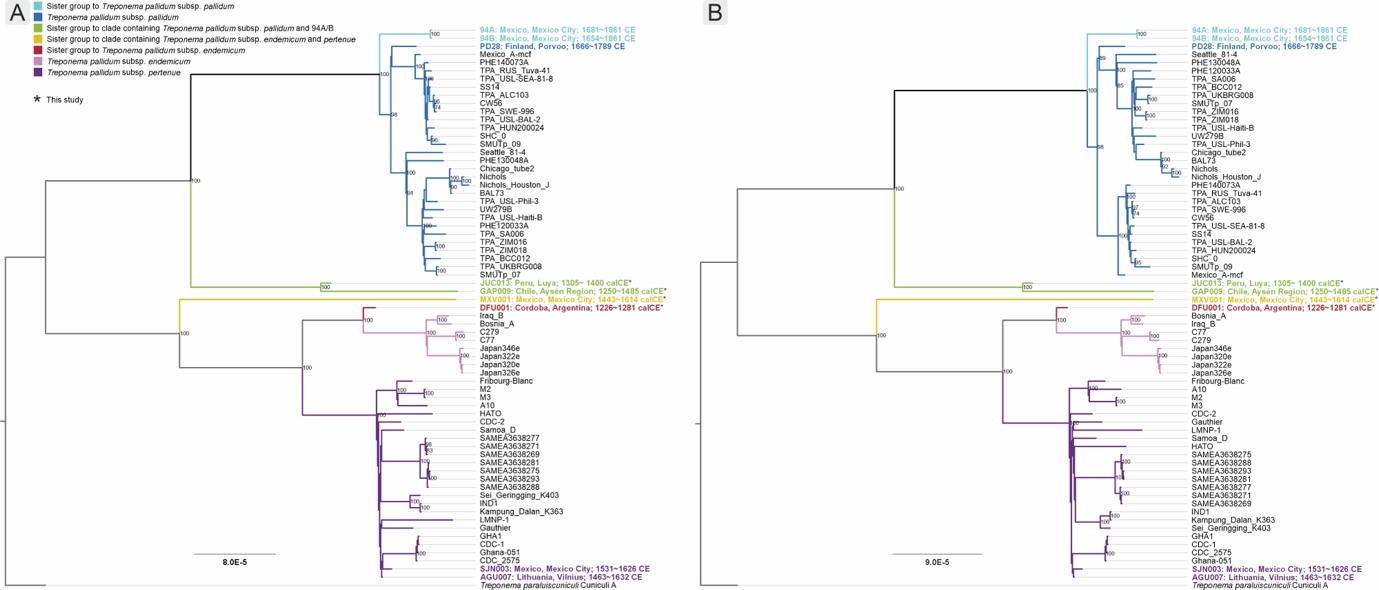


Figure S16: Maximum likelihood phylogenetic reconstruction based on mapping to reference *T. pallidum pertenue* CDC-2^82^ (A) and *T. pallidum endemicum* Bosnia A (B). The trees were generated using the 2055 and 1978 SNPs, respectively, called at 4-fold coverage with recombinant and low-confidence regions removed. The trees are based on 1000 bootstraps, branches presenting >70% bootstrap support are represented with a dot and the support value, values below the cutoff are hidden for clarity. Names of ancient genomes are coloured according to their corresponding clade.


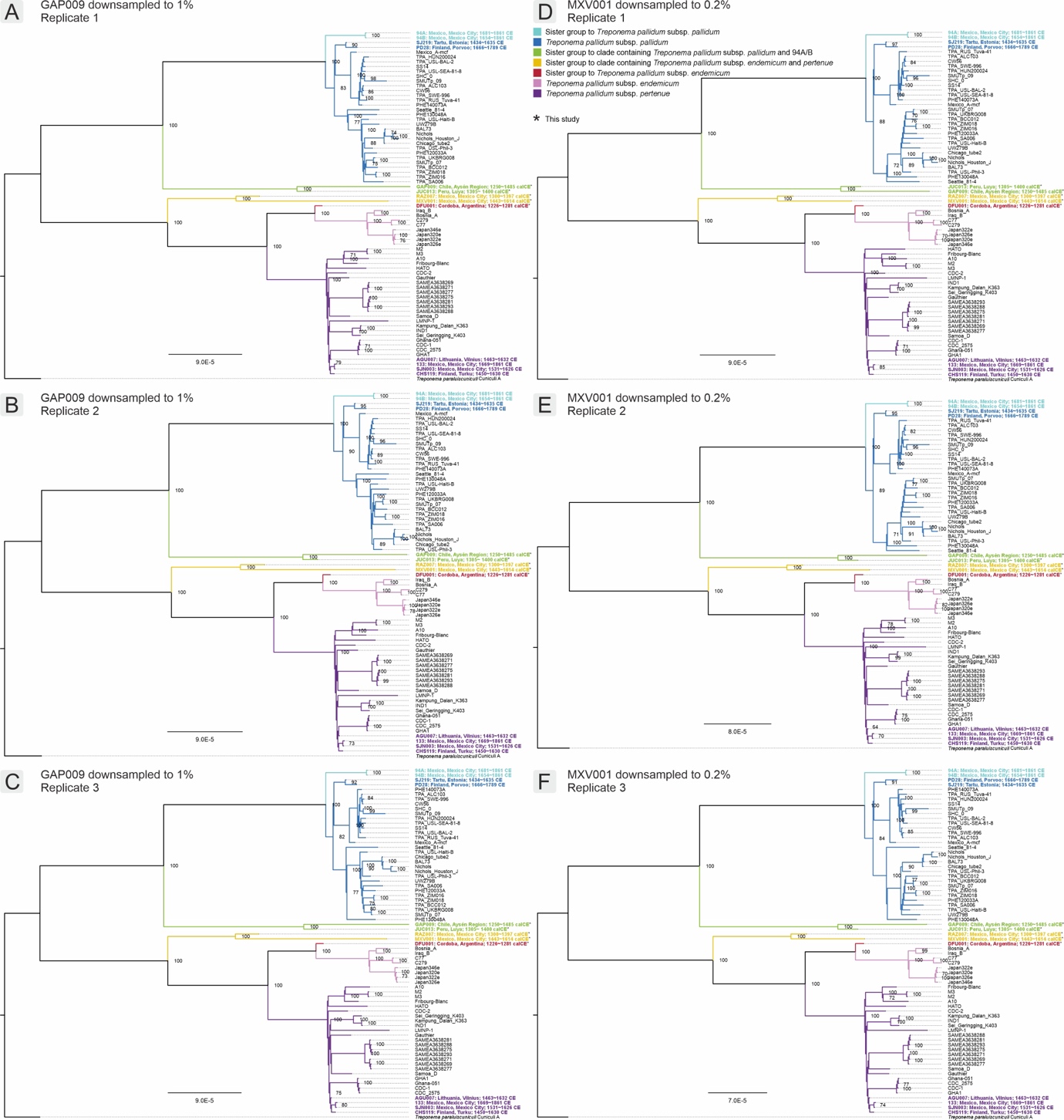


Figure S17: Maximum likelihood phylogeny reconstructed at two-fold read support with artificially reducing the coverage of genome GAP009 (A, B, and C) and MXV001 (D, E, and F) to average 4-fold. These trees were generated using 2009 (A), 1995 (B), 2008 (C), 1915 (D), 1919 (E), and 1907 (F) SNPs, respectively, with recombinant and low-confidence regions removed. The tree is based on 1000 bootstrap replicates, and branches representing <70% support at not shown. Names of ancient genomes are coloured according to their corresponding clade.

**9. Molecular Dating**


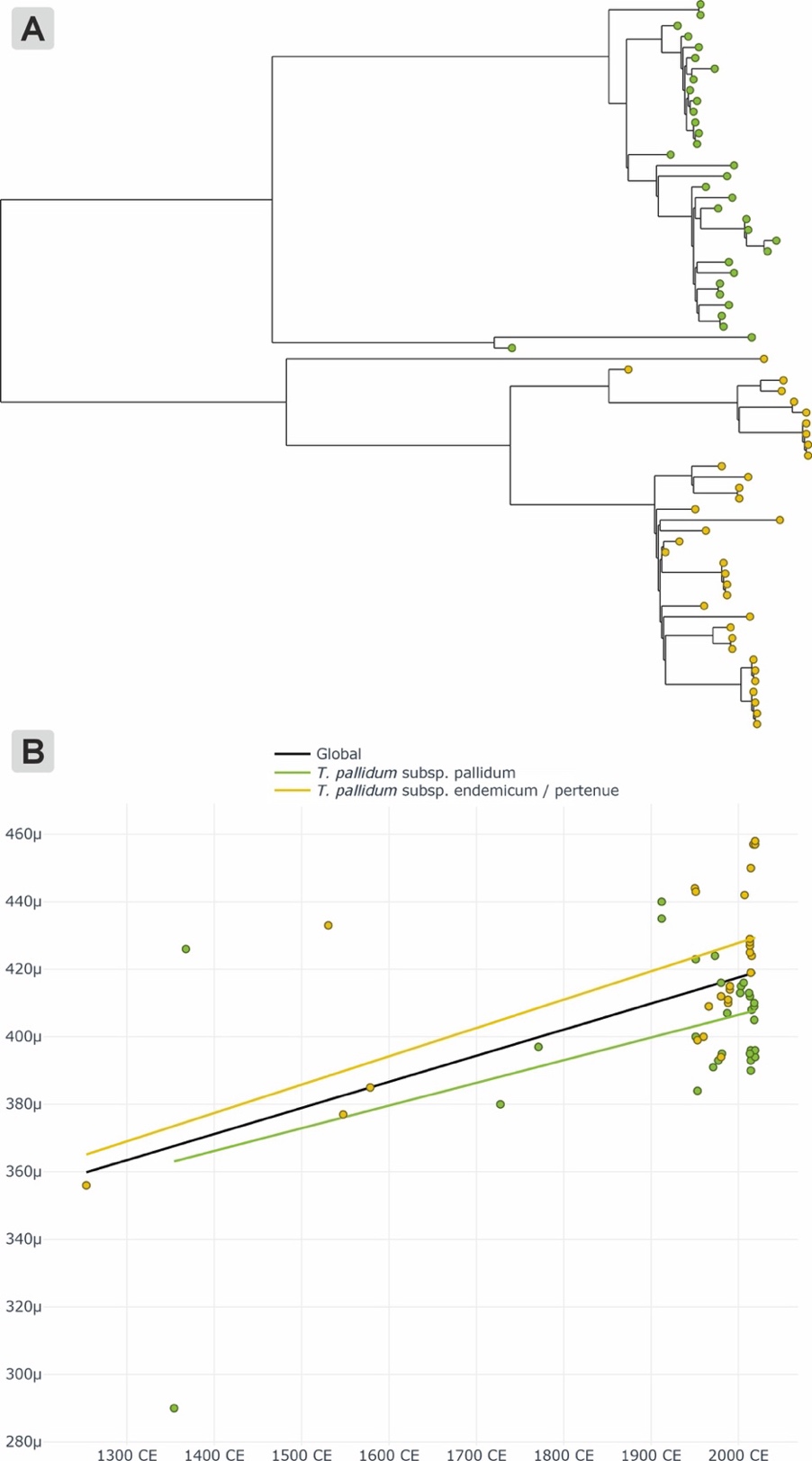


Figure S18 – Root to tip regression analysis using Clocktor2^83^. TPA shown in yellow and TPE/TEN shown in purple, and the global (full) dataset in black. A) Substitution tree estimated with RAxML following pruning of the outgroup; B) Root to tip regressions of average genomic substitutions per site against median tip dates for the dataset considered as TPA only (yellow), TPE/TEN only (purple) and the global (entire) dataset (black). The latter was fitted on the whole dataset as well as on TPA and TPE/TEN separately.

Figure S19 – Comparison of posterior and prior densities obtained using BEAST 2 for the mean clock rate (A) and time to the most recent common ancestor (B) of *Treponema pallidum*.


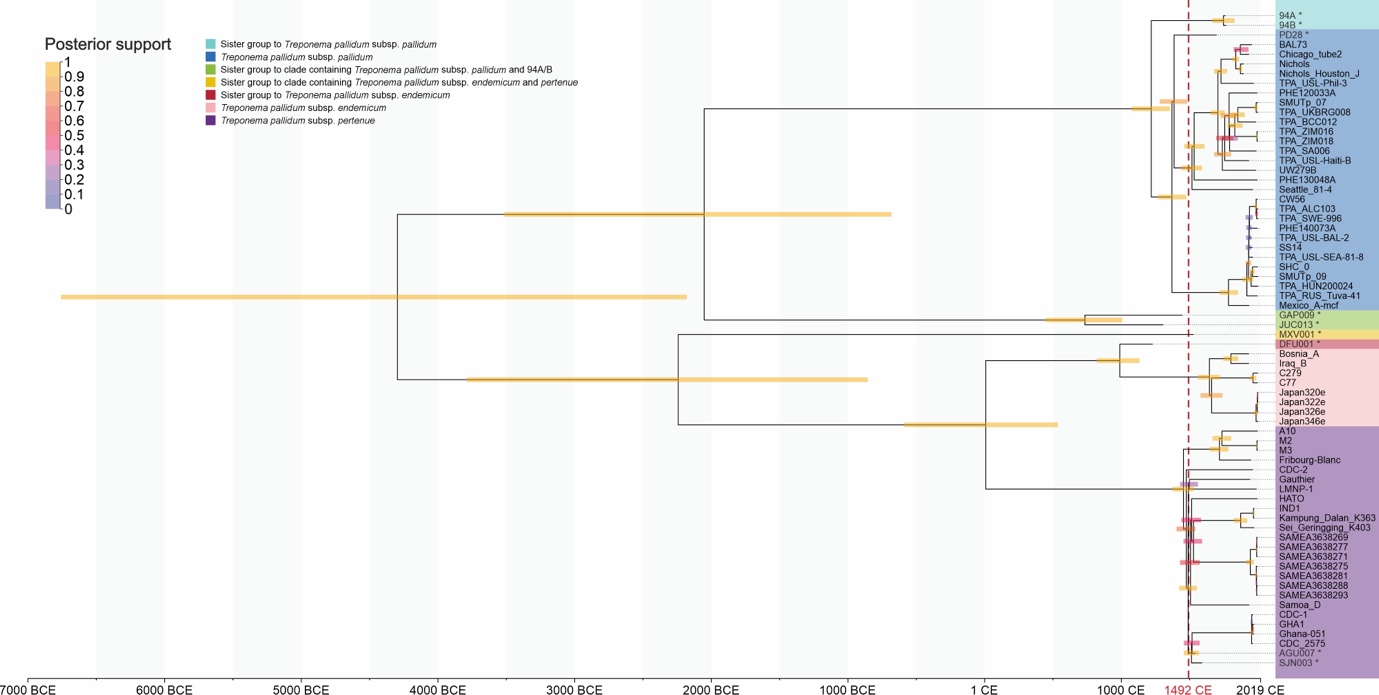


Figure S20 – Maximum clade credibility (MCC) tree generated under the time dependent rate (TDR) model.

Figure S21 – Probability densities for tMRCA estimates from the uncorrelated relaxed clock model (pink) and the time-dependent-rate (TDR) clock model (blue). Corresponding HPD 95% intervals are shown in dotted lines.

**References**

1. Jones, J. Explorations and researches concerning the destruction of the aboriginal inhabitants of America by various disease, such as syphilis, matlazahuatl, pestilence, malarial fever, and small-pox. *New Orleans Med. Surg. J.* 926–941 (1878).
2. Steinbock, R. T. *Paleopathological Diagnosis and Interpretation: Bone Diseases in Ancient Human Populations*. Charles C Thomas Pub Limited, Springfield, (1976).
3. Baker, B. J. Treponemal infection. in *The Routledge Handbook of Paleopathology* 292–305 Routledge, New York, (2022).
4. Wyman, J. Report of the Curator. Reports of the Peabody Museum of American Archaeology and Ethnology 1 (Fourth Annual Report of the Trustees), 5–24 (1871).
5. Parrot, M. J. The osseous lesions of hereditary syphilis. *The Lancet* **113**, 696–698 (1879).
6. Gann, T. Recent discoveries in Central America proving the pre-Columbian existence of syphilis in the New World. *The Lancet* **158**, 968–970 (1901).
7. Stinnesbeck, W. *et al.* New evidence for an early settlement of the Yucatán Peninsula, Mexico: The Chan Hol 3 woman and her meaning for the peopling of the Americas. *PloS One* **15**, e0227984 (2020).
8. Gerszten, P. C., Gerszten, E. & Allison, M. J. Diseases of the skull in pre-Columbian South American mummies. *Neurosurgery* **42**, 1145–1151 (1998).
9. Oliveira, R., Strauss, A., Murrieta, R., Castro, C. & Matioli, A. An Early Holocene case of congenital syphilis in South America. *Int. J. Osteoarchaeol.* DOI: https://doi.org/10.1002/oa.3180 (2022).
10. Koruyucu, M., Bayram, M., Tuna, E.B., Koray, G. & Seymen, F. Clinical findings and long-term managements of patients with amelogenesis imperfecta. *European journal of dentistry* **8**, 546-552 (2014).
11. Cook, D. C. & Powell, M. L. Piecing the puzzle together: North American treponematosis in overview. in *The Myth of Syphilis: The Natural History of Treponematosis in North America* (eds. Powell, M. L. & Cook, D. C.) 442–479 University Press of Florida, Gainesville (2005).
12. Goff, C. W. Syphilis. in *Diseases of Antiquity* (eds. Brothwell, D. R. & Sandison, A. T.) 279–294 Charles C Thomas, Springfield, (1967).
13. Johnson, F. & MacNeish, R. S. Chronometric dating. in *Prehistory of the Tehuacan Valley: Chronology and Irrigation* (ed. MacNeish, R. S.) vol. 4 3–59 University of Texas Press, Austin, (1972).
14. Wood, J. W. *et al.* The osteological paradox: problems of inferring prehistoric health from skeletal samples [and comments and reply]. *Curr. Anthropol.* **33**, 343–370 (1992).
15. Crosby Jr, A. W. The early history of syphilis: a reappraisal. *Am. Anthropol.* **71**, 218–227 (1969).
16. Saul, J. M. & Saul, F. P. The preclassic skeletons from Cuello. in *Bones of the Maya: Studies of ancient skeletons* (eds. Whittington, S. L. & Reed, D. M.) 28–50 Smithsonian Institution Press, Washington, (1997).
17. Vento Canosa, E. Antropofagia en aborígenes de Cuba. *Rev. Medica Electron.* **27**, 170–176 (2005).
18. Standen, V., Allison, M. & Arriaza, B. Patologías óseas de la población Morro-1, asociada al complejo Chinchorro: Norte de Chile. *Chungara* **13**, 175–185 (1984).
19. Filippini, J., Pezo-Lanfranco, L. & Eggers, S. Estudio regional sistemático de treponematosis en conchales (Sambaquis) precolombinos de Brasil. *Chungará Arica* **51**, 403–425 (2019).
20. Allison, M. J., Aste, G. F., Gerszten, E., Fouant, M. & Cebelin, M. La sífilis ¿una enfermedad americana? *Chungara* **9**, 275–283 (1982).
21. Burgos, J. D., Correal-Urrego, G. & Arregocés, C. Treponematosis en restos óseos precerámicos de Colombia. *Rev. Acad. Colomb. Cienc. Exactas Físicas Nat.* **19**, 237–241 (1994).
22. Roberts, C. A. & Buikstra, J. E. Bacterial infections. in *Ortner’s Identification of Pathological Conditions in Human Skeletal Remains* (ed. Buikstra, J. E.) 321–439 Elsevier, London, (2019).
23. Aufderheide, A. C., Rodríguez-Martín, C. & Langsjoen, O. *The Cambridge Encyclopedia of Human Paleopathology*. 478 Cambridge University Press, Cambridge, (1998).
24. Walker, E. G. Evidence for prehistoric cardiovascular disease of syphilitic origin on the northern plains. *Am. J. Phys. Anthropol.* **60**, 499–503 (1983).
25. Hodges, D. & Schermer, S. Treponematosis in the northern and central Great Plains: its nature and distribution. in *The Myth of Syphilis: The Natural History of Treponematosis in North America.* (eds. Powell, M. L. & Cook, D. C.) 200–227 University Press of Florida, Gainesville (2005).
26. Castro, M. M. *et al.* Thoracic aortic aneurysm in a pre-Columbian (210 BC) inhabitant of Northern Chile: Implications for the origins of syphilis. *Int. J. Paleopathol.* **13**, 20–26 (2016).
27. Kelley, M. A. Skeletal changes produced by aortic aneurysms. *Am. J. Phys. Anthropol.* **51**, 35–38 (1979).
28. Slater, E. E. & Desanctis, R. W. Diseases of the aorta. in *Heart Disease: A Textbook of Cardiovascular Medicine* (ed. Braunwald, E.) **2**,1597–1632 W.B. Saunders, Philadelphia (1980).
29. Ocampo C. & Aspillaga, E. Breves notas sobre una prospección arqueológica en los archipiélagos de las Guaitecas y de los Chonos. *Revista Chilena de Antropología* **4**, 155 (1984).
30. Reyes O., Moraga M., Méndez M. & Cherkinsky A. Maritime Hunter-Gatherers in the Chonos Archipelago (43°50’–46°50’S), Western Patagonian Channels. *J. Island Coastal Archaeol.* **10**(2), 207–231 (2015).
31. Reyes, O. 2020. *The Settlement of the Chonos Archipelago, Western Patagonia, Chile.* Springer, Cham, (2020).
32. De Cortés Hojea, F. Viaje del capitán Juan Ladrillero al descubrimiento del Estrecho de Magallanes. in *Anuario hidrográfico de la Marina de Chile* **5**, 482–520 Imprenta Nacional, Santiago, (1879).
33. Coqueugniot, H. & Weaver, T.D. Brief communication: Infracranial maturation in the skeletal collection from Coimbra, Portugal: new aging standards for epiphyseal union. *Am. J. of Phys. Anthropol.*, **134**(3), 424-437 (2007). doi:10.1002/ajpa.20683.
34. Hackett, C.J. 1976. *Diagnostic criteria of syphilis, yaws and treponarid (treponematoses) and of some other diseases in dry bones: for use in osteo-archaeology.* (Springer-Verlag Berlin Heidelberg, Berlin) (1976).
35. García Martínez, M. de los Á. *Informe de investigación de salvamento arqueológico “Manuel González 95, San Simón Tolnáhuac, Delegación Cuauhtémoc”,* (2016).
36. Talavera González, J. A. *Informe bioarqueológico de los restos óseos humanos localizados en el Proyecto de salvamento arqueológico “Manuel González No. 95., San Simón Tolnáhuac, Delegación Cuauhtémoc, Ciudad de México*. (2017).
37. Al Qahtani, S. J. *Atlas of tooth development and eruption*. Barts and the London School of Medicine and Dentistry, Queen Mary University of London, London, (2009). www.qmul.ac.uk/dentistry/atlas.
38. Scheuer, L. & Black, S. *The juvenile skeleton*. London: Elsevier, London, (2004).
39. Buikstra, J. E. Ortner’s Identification of pathological conditions in human skeletal remains. (2019).
40. Koschmieder, K. *Proyecto Arqueológico Jucusbamba. Segunda Temporada 2009-2010* (D. Forschungsgemeinschaft). Presented to Instituto Nacional de Cultura (2010).
41. Bass, W. M. *Human osteology: a laboratory and field manual.* Missouri Archaeological Society, Columbia, (2005).
42. Buikstra, J. E. & Ubelaker, D. H. *Standards for data collection from human skeletal remains*. Arkansas Archaeological Survey Research Series No. 44, Fayetteville: Arkansas Archaeological Survey (1994).
43. Schaefer, M., Black, S.M. & Scheuer, L. *Juvenile osteology*. London: Academic Press, (2009).
44. Dentoni, M. N., Seldes, V., Una, L., Aranda, C., Rodríguez, P., Callará, R. V., Ponce, R. H., V. A., & Fabra, M. First studies on dental fluorosis repopulation archaeological populations of the province of Cordoba (Argentina). *Revista Argentina de Antropología Biológica* **25**(1), 1-20 (2023).
45. Zhang, X., Zhao, Y., Niu, Y., Wang, Z. & Zeng, W. Two probable cases of mastoiditis in a cemetery from the Warring States to Han Dynasty (475 BCE–220 CE) in Qufu, Shandong Province, China. *International Journal of Paleopathology* **30**, 77-84 (2020).
46. Göhring, A. Allen’s fossa—An attempt to dissolve the confusion of different nonmetric variants on the anterior femoral neck. *International Journal of Osteoarchaeology*, **31**(4), 513-522 (2021).
47. Edmonds, E.W. & Polousky, J. A review of knowledge in osteochondritis dissecans: 123 years of minimal evolution from König to the ROCK study group. *Clinical Orthopaedics and Related Research* **471**, 1118-1126 (2013).
48. Ceja, M. *Azcapotzalco una Población del Postclásico*. Bachelor thesis, ENAH. (1987).
49. Santamarina N., C. *El Sistema de Dominación Azteca. El Imperio Tepaneca. Una Aproximación desde la Antropología Política.* Académica Española (2011).
50. Castañeda de la Paz, M. Dos parcialidades étnicas en Azcapotzalco: Mexicapan y Tepanecapan. *Estud. Cult. Náhuatl* ***46***, 223–248. (2013).
51. Facchini, F. & Veschi, S. Age determination on long bones in a skeletal subadults sample (b-12 years). *Collegium antropologicum*, ***28*,** 89-98 (2004).

1. Stuart‐Macadam, P. Porotic hyperostosis: representative of a childhood condition. *Am. J. Phys. Anthropol.* ***66***, 391-398 (1985).
2. Rinaldo, N., Zedda, N., Bramanti, B., Rosa, I. & Gualdi-Russo, E. How reliable is the assessment of porotic hyperostosis and cribra orbitalia in skeletal human remains? A methodological approach for quantitative verification by means of a new evaluation form. *Archaeol. Anthropol. Sci.* **11**, 3549–3559 (2019).

doi.org/10.1007/s12520-019-00780-0.

1. Hershkovitz, I., Greenwald, C. M., Latimer, B., Jellema, L. M., Wish‐Baratz, S., Eshed, V., Dutour, O. & Rothschild, B. M. Serpens endocrania symmetrica (SES): a new term and a possible clue for identifying intrathoracic disease in skeletal populations. *Am. J. Phys. Anthropol.* **118**, 201-216 (2002).
2. Renaud, G., Stenzel, U., & Kelso, J. leeHom: adaptor trimming and merging for Illumina sequencing reads. *Nucleic Acids Res*, **42**(18), e141–e141 (2014). doi.org/10.1093/nar/gku699.
3. Li, H., & Durbin, R. Fast and accurate short read alignment with Burrows-Wheeler transform. *Bioinformatics (Oxford, England)*, *25*(14), 1754–1760. (2009). doi.org/10.1093/bioinformatics/btp324.
4. Mathieson, I., Lazaridis, I., Rohland, N., Mallick, S., Patterson, N., Roodenberg, S. A., Harney, E., Stewardson, K., Fernandes, D., Novak, M., Sirak, K., Gamba, C., Jones, E. R., Llamas, B., Dryomov, S., Pickrell, J., Arsuaga, J. L., de Castro, J. M. B., Carbonell, E., … Reich, D. Genome-wide patterns of selection in 230 ancient Eurasians. *Nature*, **528**(7583), 499–503. (2015). doi.org/10.1038/nature16152.
5. Krueger, F. *Trim Galore* (v0.6.2). Babraham Bioinformatics (2019).
6. Krueger, F. *FastQC* (v0.11.8). Babraham Bioinformatics (2018).
7. Martin, M. Cutadapt removes adapter sequences from high-throughput sequencing reads. *EMBnetjournal*, **17** (2011).
8. Herbig, A., Maixner, F., Bos, K. I., Zink, A., Krause, J., & Huson, D. H. MALT: Fast alignment and analysis of metagenomic DNA sequence data applied to the Tyrolean Iceman. *BioRxiv*, 50559. (2016). doi.org/10.1101/050559.
9. Hübler, R., Key, F. M., Warinner, C., Bos, K. I., Krause, J., & Herbig, A. HOPS: automated detection and authentication of pathogen DNA in archaeological remains. *Genome Biol*, **20**(1), 280. (2019).

doi.org/10.1186/s13059-019-1903-0.

1. Dabney, J., Meyer, M., & Pääbo, S. Ancient DNA damage. *Cold Spring Harbor Perspectives in Biology*, **5**(7). (2013). doi.org/10.1101/CSHPERSPECT.A012567.
2. Longin, R. New method of collagen extraction for radiocarbon dating, Nature **230**, 241–242 (1971).
3. Brock, F., Higham, T., Ditchfield, P., Bronk Ramsey, C. Current pretreatment methods for AMS radiocarbon dating at the Oxford Radiocarbon Accelerator Unit (ORAU), *Radiocarbon* **52**, 103–112 (2010).
4. Reimer, P.J., Austin, W.E.N., Bard, E., Bayliss, A., Blackwell, P.G., Ramsey, C.B., Butzin, M., Cheng, H., Edwards, R.L., Friedrich, M., Grootes, P.M., Guilderson, T.P., Hajdas, I., Heaton, T.J., Hogg, A.G., Hughen, K.A., Kromer, B., Manning, S.W., Muscheler, R., Palmer, J.G., Pearson, C., Plicht, J.v.d., Reimer, R.W., Richards, D.A., Scott, E.M., Southon, J.R., Turney, C.S.M., Wacker, L., Adolphi, F., Büntgen, U., Capano, M., Fahrni, S.M., Fogtmann-Schulz, A., Friedrich, R., Köhler, P., Kudsk, S., Miyake, F., Olsen, J., Reinig, F., Sakamoto, M., Sookdeo, A., Talamo, S. The IntCal20 Northern Hemisphere Radiocarbon Age Calibration Curve (0–55 cal kBP), *Radiocarbon* **62**, 725–757 (2020).
5. Bronk Ramsey, C. Bayesian analysis of radiocarbon dates, *Radiocarbon* **51**, 337–360 (2009).
6. Pětrošová, H., Pospíšilová, P. Strouhal, M., Čejková D, Zobaníková, M. et al. Resequencing of. Treponema pallidum ssp. Pallidum strains Nichols and SS14: correction of sequencing errors resulted in increased separation of syphilis treponeme clusters. PLOS ONE 8, e74319 (2013) doi: 10.1371/journal.pone.0074319.
7. Keller, M. Spyrou, M.A., Scheib, C.L., Neumann, G.U., Kröpelin, A. et al. Ancient *Yersinia pestis* genomes from across Western Europe reveal early diversification during the First Pandemic (541-750). *Proc. Nat.l Acad. Sci. USA* **116**, 12363-12372 (2019).
8. van der Auwera, G. A., Carneiro, M. O., Hartl, C., Poplin, R., del Angel, G., Levy-Moonshine, A., Jordan, T., Shakir, K., Roazen, D., Thibault, J., Banks, E., Garimella, K. v, Altshuler, D., Gabriel, S., & DePristo, M. A. From FastQ Data to High-Confidence Variant Calls: The Genome Analysis Toolkit Best Practices Pipeline. *Current Protocols in Bioinformatics*, **43**, 11.10.1-11.10.33. (2013). doi.org/https://doi.org/10.1002/0471250953.bi1110s43.
9. Bos, K. I., Harkins, K. M., Herbig, A., Coscolla, M., Weber, N., Comas, I., Forrest, S. A., Bryant, J. M., Harris, S. R., Schuenemann, V. J., Campbell, T. J., Majander, K., Wilbur, A. K., Guichon, R. A., Wolfe Steadman, D. L., Cook, D. C., Niemann, S., Behr, M. A., Zumarraga, M., … Krause, J. Pre-Columbian mycobacterial genomes reveal seals as a source of New World human tuberculosis. *Nature*, **514**(7523), 494–497 (2014). doi.org/10.1038/nature13591.
10. Majander, K., Pfrengle, S., Kocher, A., Neukamm, J., du Plessis, L., Pla-Díaz, M., Arora, N., Akgül, G., Salo, K., Schats, R., Inskip, S., Oinonen, M., Valk, H., Malve, M., Kriiska, A., Onkamo, P., González-Candelas, F., Kühnert, D., Krause, J., & Schuenemann, V. J. Ancient Bacterial Genomes Reveal a High Diversity of Treponema pallidum Strains in Early Modern Europe. *Current Biology*, **30**, 3788-3803.e10 (2020). doi.org/https://doi.org/10.1016/j.cub.2020.07.058.
11. Wright, E.S., and Vetsigian, K.H. Quality filtering of Illumina index reads mitigates sample cross-talk. BMC Genomics 17, 876, (2016).

doi.org/10.1186/s12864-016-3217-x

1. Schuenemann, Verena J., et al. "Historic Treponema pallidum genomes from Colonial Mexico retrieved from archaeological remains." *PLoS neglected tropical diseases* **12**: e0006447 (2018).
2. Morgulis, A., Gertz, E. M., Schäffer, A. A., & Agarwala, R. A Fast and Symmetric DUST Implementation to Mask Low-Complexity DNA Sequences. *Journal of Computational Biology*, **13**, 1028–1040. (2006) doi.org/10.1089/cmb.2006.13.1028.
3. Croucher, N. J., Page, A. J., Connor, T. R., Delaney, A. J., Keane, J. A., Bentley, S. D., Parkhill, J., & Harris, S. R. Rapid phylogenetic analysis of large samples of recombinant bacterial whole genome sequences using Gubbins. *Nucleic Acids Research*, **43**, e15–e15. (2015). doi.org/10.1093/nar/gku1196
4. Didelot, X., & Wilson, D. J. ClonalFrameML: Efficient Inference of Recombination in Whole Bacterial Genomes. *PLOS Computational Biology*, **11**, e1004041-. (2015). doi.org/10.1371/journal.pcbi.1004041
5. Price, M. N., Dehal, P. S., & Arkin, A. P. FastTree: Computing Large Minimum Evolution Trees with Profiles instead of a Distance Matrix. *Molecular Biology and Evolution*, **26**, 1641–1650 (2009). doi.org/10.1093/molbev/msp077.
6. Kröpelin A. *SNP_Evaluation*. (2018). github.com/andreasKroepelin/SNP_Evaluation.
7. Kozlov, A. M., Darriba, D., Flouri, T., Morel, B., & Stamatakis, A. RAxML-NG: a fast, scalable and user-friendly tool for maximum likelihood phylogenetic inference. *Bioinformatics*, **35**, 4453–4455, doi.org/10.1093/bioinformatics/btz305 (2019).
8. Giffin, K., Lankapalli, A. K., Sabin, S., Spyrou, M. A., Posth, C., Kozakaitė, J., Friedrich, R., Miliauskienė, Ž., Jankauskas, R., Herbig, A., & Bos, K. I. A treponemal genome from an historic plague victim supports a recent emergence of yaws and its presence in 15th century Europe. *Sci. Rep*. **10**(1), 9499 (2020). doi.org/10.1038/s41598-020-66012-x
9. Čejková, D., Zoaníková, M., Chen, L., Pospíšilová, P., Strouhal, M., et al. Whole genome sequences of three Treponema pallidum ssp. Pertenue strains: yaws and syphilis treponemes differ in less than 0.2% of the genome sequence. *PLoS. Negl. Trop. Dis.* **6**, e1471 doi: 10.1371/journal.pntd.0001471 (2012).
10. Featherstone, L.A., Rambaut, A., Duchene, S. and Wirth, W. Clocktor2: Inferring global and local strict molecular clocks using root-to-tip regression. **bioRxiv** 2023.07.13.548947 (2023).
