# Supplementary material for: Ancient genomes reveal a deep history of Treponema pallidum in the Americas
Source: Nature. 2024 Dec 18;640(8057):186–93. doi: 10.1038/s41586-024-08515-5 (PMC11964931; doi:10.1038/s41586-024-08515-5)
Supplement: Supplementary file 2 — Reporting Summary [file 41586_2024_8515_MOESM2_ESM.pdf]

nature portfolio

Corresponding author(s): Kirsten I.Bos
Johannes Krause

Last updated by author(s): Dec 6, 2024

Reporting Summary

Nature Portfolio wishes to improve the reproducibility of the work that we publish. This form provides structure for consistency and transparency in reporting. For further information on Nature Portfolio policies, see our Editorial Policies and the Editorial Policy Checklist.

Statistics

For all statistical analyses, confirm that the following items are present in the figure legend, table legend, main text, or Methods section.

n/a	Confirmed	
☐	☒	The exact sample size (*n*) for each experimental group/condition, given as a discrete number and unit of measurement
☐	☒	A statement on whether measurements were taken from distinct samples or whether the same sample was measured repeatedly
☒	☐	The statistical test(s) used AND whether they are one- or two-sided<br>*Only common tests should be described solely by name; describe more complex techniques in the Methods section.*
☒	☐	A description of all covariates tested
☒	☐	A description of any assumptions or corrections, such as tests of normality and adjustment for multiple comparisons
☐	☒	A full description of the statistical parameters including central tendency (e.g. means) or other basic estimates (e.g. regression coefficient) AND variation (e.g. standard deviation) or associated estimates of uncertainty (e.g. confidence intervals)
☒	☐	For null hypothesis testing, the test statistic (e.g. *F*, *t*, *r*) with confidence intervals, effect sizes, degrees of freedom and *P* value noted<br>*Give P values as exact values whenever suitable.*
☐	☒	For Bayesian analysis, information on the choice of priors and Markov chain Monte Carlo settings
☒	☐	For hierarchical and complex designs, identification of the appropriate level for tests and full reporting of outcomes
☒	☐	Estimates of effect sizes (e.g. Cohen's *d*, Pearson's *r*), indicating how they were calculated

Our web collection on statistics for biologists contains articles on many of the points above.

Software and code

Policy information about availability of computer code

Data collection	N/A
Data analysis	nf-core/eager v2.4.4<br>Nextflow v21.04.1<br>FastQC v0.11.9<br>MultiQC v1.12<br>AdapterRemoval 22.3.2<br>Samtools v1.12<br>endorS.py v0.4<br>DeDup v0.12.8<br>Picard MarkDuplicates v2.26.0<br>Qualimap v2.2.2-dev<br>Preseq v3.1.1<br>GATK Unified Genotyper v3.8<br>DamageProfile v0.4.9<br>bamUtil v1.0.15<br>bcl2fastq v2.18.0.12<br>leeHom v1.2.15<br>Bam2fastq v1.1.0<br>TrimGalorev0.6.6<br>MALT v0.5.2

HOPS v0.35
Excel v4.4
MultiVCFAnalyzer v0.85.2
Dust masker v1.0.0
Gubbins v3.2.1
ClonalFrame v1.12
FastTree v2.1.11
SNPEval v1.0
RAxML-NG v1.10
FigTree v1.4.4
BWA v0.7.17-r1188
Samtools v1.12
endorS.py v0.4
DeDup v0.12.8
Picard MarkDuplicates v2.26.0
Qualimap v2.2.2-dev
Preseq v3.1.1
GATK UnifiedGenotyper vPicked
DamageProfiler v0.4.9
bwa v0.7.12
mapDamage v2.0
pileup Caller v8.2.2
HaploGrep2 v2.4.0
HAPLOFIND
Haplocheck v1.0.0
ISOGG Y-DNA Haplogroup Tree v15.73
ADMIXTURE v1.3
AdmixturePlotter
Xerxes CLI (Poseidon)
BEDtools v2.30.0
ggplot v4.2.2
snpEff v3.1i
Clocktor2
BEAST2 v2.6.7
BETS
BEAUTi v2.6.7
Tracer v1.7.1
Treeannotator
BEAST 1.10.4

For manuscripts utilizing custom algorithms or software that are central to the research but not yet described in published literature, software must be made available to editors and reviewers. We strongly encourage code deposition in a community repository (e.g. GitHub). See the Nature Portfolio guidelines for submitting code & software for further information.

Data

Policy information about availability of data

All manuscripts must include a data availability statement. This statement should provide the following information, where applicable:

- Accession codes, unique identifiers, or web links for publicly available datasets
- A description of any restrictions on data availability
- For clinical datasets or third party data, please ensure that the statement adheres to our policy

Data are accessible via ENA Project ID PRJEB62879.
Data from other public sources utilized in this study can be accessed via information contained in Table S8 (T. pallidum data) or Table S27 (human data). Mapping to the human genome was performed against reference GCF_000001405.13.

Research involving human participants, their data, or biological material

Policy information about studies with human participants or human data. See also policy information about sex, gender (identity/presentation), and sexual orientation and race, ethnicity and racism.

Reporting on sex and gender	N/A
Reporting on race, ethnicity, or other socially relevant groupings	N/A
Population characteristics	N/A
Recruitment	N/A

Ethics oversight | N/A

Note that full information on the approval of the study protocol must also be provided in the manuscript.

Field-specific reporting

Please select the one below that is the best fit for your research. If you are not sure, read the appropriate sections before making your selection.

☐ Life sciences ☐ Behavioural & social sciences ☒ Ecological, evolutionary & environmental sciences

For a reference copy of the document with all sections, see nature.com/documents/nr-reporting-summary-flat.pdf

Ecological, evolutionary & environmental sciences study design

All studies must disclose on these points even when the disclosure is negative.

Study description	Analyses of five Treponemal pallidum genomes obtained from archaeological human tissues.
Research sample	Archaeological human tissues.
Sampling strategy	We report five ancient genomes.
Data collection	Data were collected by multiple coauthors through archaeological excavation, on-site sampling, in-lab sub-sampling, DNA sequencing, and DNA computational analysis.
Timing and spatial scale	Data are from archaeological human tissues from the precontact period in the Americas.
Data exclusions	No data were excluded.
Reproducibility	No attempts were made to repeat the retrieval of ancient DNA, as archaeological tissues are a non-renewable resource.
Randomization	N/A
Blinding	Not relevant because we did not work with living subjects.

Did the study involve field work? ☐ Yes ☒ No

Reporting for specific materials, systems and methods

We require information from authors about some types of materials, experimental systems and methods used in many studies. Here, indicate whether each material, system or method listed is relevant to your study. If you are not sure if a list item applies to your research, read the appropriate section before selecting a response.

Materials & experimental systems

n/a	Involved in the study	
☒	☐	Antibodies
☒	☐	Eukaryotic cell lines
☐	☒	Palaeontology and archaeology
☒	☐	Animals and other organisms
☒	☐	Clinical data
☒	☐	Dual use research of concern
☒	☐	Plants

Methods

n/a	Involved in the study	
☒	☐	ChIP-seq
☒	☐	Flow cytometry
☒	☐	MRI-based neuroimaging

Palaeontology and Archaeology

Specimen provenance

1. Chile, Consejo de Monumentos Nacionales de Chile Doc. N° 063 dated January 24, 2003

2. Mexico, Council of Archaeology, the Department of Archaeological Salvage/Department of Physical Anthropology, and the National Institute of Anthropology and History, Estudio molecular de posibles casos de treponematosis y otras patologías antes del periodo de contacto: el caso de la Cuenca de México en los periodos Clásico y Postclásico; official notice number: 401.1S.3-2019/1201; official export permit number: 401-3-32 AA-76

3. Peru, Peruvian Ministry of Culture, Project permit RD000013-2017/DGM/VMPCIC/MC and export permit RV167-2017-VMPCIC-MC

4. Argentina, authorization from the Agencia Córdoba Cultura and the Instituto Nacional de Antropología y Pensamiento Latinoamericano (INAPL) N° DI-2021-87-APN-INAPL#MC dated December 9th, 2021, and N° DI-2022-38-APN-INAPL#MC dated May 19th, 2022.

5. Mexico, Archaeological Council of the National Institute of Anthropology and History (Official Dispatch Numbers: 401-1S.3-2021/1414 and 401.1S.3-2024/72; export: 401-3-440 AA-01-2022)

Specimen deposition	N/A
Dating methods	Calibrated Radiocarbon dates, Curt-Engelhorn-Centre for Archaeometry (Mannheim, Germany)

☒ Tick this box to confirm that the raw and calibrated dates are available in the paper or in Supplementary Information.

Ethics oversight	No ethical guidance required for this study

Note that full information on the approval of the study protocol must also be provided in the manuscript.
